# Causal Relationship Between Sleep Traits and Risk of Systemic Lupus Erythematosus: A Two-Sample Mendelian Randomization Study

**DOI:** 10.3389/fimmu.2022.918749

**Published:** 2022-06-17

**Authors:** Ni Sang, Rui-Chen Gao, Meng-Yao Zhang, Zhen-Zhen Wu, Zhen-Gang Wu, Guo-Cui Wu

**Affiliations:** School of Nursing, Anhui Medical University, Hefei, China

**Keywords:** systemic lupus erythematosus, sleep traits, mendelian randomization study, causal relationship, autoimmune disease

## Abstract

A correlation between sleep and systemic lupus erythematosus (SLE) has been observed in a number of prior investigations. However, little is known regarding the potential causative relationship between them. In this study, we selected genetic instruments for sleep traits from pooled data from published genome-wide association studies (GWAS). Independent genetic variants associated with six sleep-related traits (chronotype, sleep duration, short sleep duration, long sleep duration, insomnia, and daytime sleepiness) were selected as instrumental variables. A two-sample Mendelian randomization (TSMR) study was first conducted to assess the causal relationship between sleep traits and SLE (7219 cases versus 15,991 controls). The reverse MR analysis was then used to infer the causal relationship between SLE and sleep traits. Inverse variance weighted (IVW), MR Egger, Weighted median, and Weighted mode were applied to perform the primary MR analysis. MR Egger regression and the Mendelian randomization pleiotropy residual sum and outlier (MR-PRESSO) test were used to detect horizontal pleiotropy, and Cochran’s *Q* was used to detect heterogeneity. In studies of the effect of sleep traits on SLE risk, the IVW method demonstrated no causal relationship between chronotype, sleep duration, short sleep duration, long sleep duration, insomnia, daytime sleepiness and SLE risk. The remaining three methods agreed with the results of IVW. In studies of the effect of SLE on the risk of sleep traits, neither IVW, MR Egger, Weighted median, nor Weighted mode methods provided evidence of a causal relationship between SLE and the risk of sleep traits. Overall, our study found no evidence of a bidirectional causal relationship between genetically predicted sleep traits and SLE.

## Introduction

Systemic lupus erythematosus (SLE) is an autoimmune disease that characterized by the involvement of multiple organs such as skin, joints, kidneys, lungs, central nervous system, and hematopoietic system with many complications (1). Globally, the estimated prevalence of SLE in adults ranges from 30 to 150 per 100, 000, with an incidence ranging from 2.2 to 23.1 per 100,000 per year (2). Until now, the etiology of SLE is complex and unclear (3). Therefore, exploring the pathogenesis of SLE is helpful for formulating prevention and treatment strategies.

The prevalence of sleep disorders in SLE patients has been reported to be at a high level, ranging from 55% to 85% (4). However, they could not conclude whether sleep disturbance increases the risk of SLE. Several studies have shown that SLE patients have poorer sleep quality as compared to the general population (5, 6). This may lead to lower quality of life, more fatigue, more stress, and emotional discomfort among SLE patients (7). In the mouse model has suggested that sleep deprivation is considered to be a risk factor for the development of SLE (8). As a result, sleep may be causally connected to SLE and explained through a variety of mechanisms. Studies have demonstrated an association between sleep deprivation and gut microbiota (9). Sleep duration can also affect the functional correlation of intestinal microbiota and its metabolic integrity (10). In addition, a recent Mendelian randomization (MR) analysis showed that intestinal microbial components had a causal impact on the risk of SLE (11). Therefore, sleep may have an impact on SLE pathogenesis through gut microbes. In addition, there are a few studies (12, 13) demonstrating that sleep disorders can increase the risk of autoimmune diseases, such as alopecia areata, rheumatoid arthritis (RA), ankylosing spondylitis (AS), and Sjogren’s syndrome (SS). Two retrospective cohort studies have shown a causal relationship between obstructive sleep apnea (OSA) and RA, that is, OSA increases the risk of RA, and RA also leads to an increased risk of OSA development (14, 15). In addition, a variety of cytokines are associated with the activity of SLE, such as type I interferon, IL-6, IL-10, IL-15, IL-18, and tumor necrosis factor (TNF) (16). IL-6 and TNF are closely related to sleep (17). This suggests that sleep may promote the occurrence and development of SLE. In adition, sleep is bi-directionally associated with the immune system, and sleep can modulate the immune system (18). The pathogenesis of SLE is involved in abnormal autoimmune reactions (19). As a result, sleep may have a role in the immunological pathogenesis of autoimmune illnesses, but whether there is a causative association between sleep and SLE remains unknown.

Mendelian randomization (MR) is a statistical approach that was used to assess the causal relationship between exposure factors and disease outcomes (20). In MR analysis, genetic variations are used as instrument variables (IVs), and genetic variation is relatively less affected by measurement error or bias. Therefore, MR is widely used to explore causal associations. Two-sample MR (TSMR), which gets exposure and outcome effect sizes from two independent datasets, is mostly used to connect analytical GWAS data with disease outcomes but lacks intermediate phenotypic data in very large datasets (21).

Therefore, in the present study, we collected published sleep-related traits and SLE data from large genetic studies to explore whether there is a bidirectional causal relationship between sleep and SLE by bidirectional TSMR analysis.

## Materials and Methods

### Data Sources

Data sources for sleep-related traits and SLE have been compiled and made publicly available online. (**Supplementary Table S1**). Previously published single-nucleotide polymorphisms (SNPs) associated with each sleep phenotype (exposure) were selected as IVs. In reverse MR analysis, sleep-related traits used summary level data from UK Biobank, which were already publicly available(http://www.kp4cd.org/dataset_downloads/sleep). Participants in our source of data for MR analysis were primarily of European ancestry. Since this study was based on published data, no ethical approval or informed consent was required.

#### Chronotype

Chronotype is defined by an individual’s tendency for earlier or later sleep timing, also known as circadian preference. Genetic association estimates for chronotype were retrieved from a GWAS among 403,195 individuals of European ancestry in the UK Biobank (22).

#### Sleep Duration

Genetic association data for sleep duration was obtained by published GWAS associations of 446,118 adults of European ancestry from the UK Biobank. Sleep duration was analyzed as a continuous variable, yielding two category variables. Sleep duration < 7 hours was defined as short sleep duration (N = 106,192), while sleep duration ≥ 9 hours was defined as long sleep duration (N = 34,184), and 7 hours ≤ sleep duration < 9 hours was defined as normal sleep duration (N = 305,742). At the same time, extreme responses with sleep duration of less than 3 hours or more than 18 hours were excluded (23).

#### Insomnia

Insomnia is a frequent sleep difficulty that can lead to daytime impairment and also occurs with adequate sleep opportunities (24). The genetic association of insomnia was obtained by genome-wide association analysis of a total of 13,31,010 individuals from the UK Biobank (N = 386,533) and 23 and Me (N = 944,477) (25).

#### Daytime Sleepiness

The genetic association for daytime sleepiness was obtained from a GWAS analysis of 452,071 individuals in the UK Biobank (26).

#### SLE

Genetic variants in SLE were extracted from a large GWAS study of UKB that included 7,219 cases of European ancestry and 15,991 controls of European ancestry (27).

### Selection of Instrumental Variables

In this study, the criteria for a genetic variation to become an instrumental variable was as follows (28): (1) SNPs are strongly associated with sleep traits; (2)SNPs are independent of confounders affecting sleep traits and SLE; (3) SNPs are not directly associated with SLE, and they can only exert effects on SLE through the sleep pathway. Therefore, we used the following steps to select the best IVs associated with sleep traits.

At the beginning, we extracted SNPs that demonstrated a strong association with sleep traits from already published data, with *P* < 5×10^-8^ as the primary screening condition. To ensure that the instruments used for exposure were independent, we excluded SNPs that were in linkage disequilibrium (LD) (*r^2^* < 0.001, clumping window = 10,000kb). We then extracted the instrumental variables of the sleep traits identified above in the SLE GWAS. For specific SNPs that were absent in the SLE GWAS, proxy SNPs were sought by 1000 Genomes European reference population (*r^2^* > 0.8). Palindromic SNPs were excluded. Afterwards, we reconciled the exposure data and the outcome data, which means that the effect of the SNP on the exposure and the effect on the outcome each correspond to the same allele. We also calculated the *F* statistic to eliminate the bias caused by weak instrumental variables in the results. The *F* statistic is calculated as *F* = *R* ^2^ (*n*-*k*-1)/[*k* (1-*R^2^*)]. *R^2^* reflects the degree to which the instrumental variable explains the exposure.

### MR Analysis

In the current study, MR computational models were utilized to determine if there was a bidirectional causal relationship between sleep traits and SLE risk using inverse-variance weighted (IVW), MR Egger, Weighted median, and Weighted mode. We adopted IVW as the main method for MR analysis. Point estimates obtained from IVW MR are equivalent to a weighted linear regression of SNP-outcome associations for SNP-exposure associations, regardless of intercept (29). When using the IVW method, it is necessary to ensure that the SNPs are not pleiotropy, otherwise, the results can be very biased. The MR-Egger method can be used to detect violations of instrumental variable assumptions but can be biased and inflate type I error (30). In contrast to IVW, the MR-Egger method takes into account the presence of an intercept term. The Weighted median method can prevent invalid tools, and it can also provide consistent estimates of causal effects if 50% of the information is analyzed from genetic variation in invalid IV (31). Weighted mode methods are less capable of detecting causal effects, but also have fewer biases (32).

### Sensitivity Analysis

In the current study, Cochran’s *Q* test was used to detect heterogeneity. The inter-instrument *Q*-test is mainly used to explore heterogeneity due to multiplicity or other reasons (33). MR-Egger regression test was used to detect pleiotropy. If the intercept term is not 0, then horizontal pleiotropy is demonstrated (34). The Mendelian Randomization Pleiotropy Residual Sum and Outlier (MR-PRESSO) test was used to detect and correct for horizontal pleiotropy by removing outliers (35). More rigorously, we used the leave-one-out method for sensitivity analysis to further validate the robustness of the results. Given the presence of potential pleiotropy, we retrieved potential secondary phenotypes for each SNP used as IVs from PhenoScanner (http://www.phenoscanner.medschl.cam.ac.uk/), excluded SNPs that may control for confounding traits, and then performed sensitivity analysis again.

### Statistical Analysis

TSMR analysis was performed using the’TwoSampleMR’package. MR-PRESSO test used the’MRPRESSO’package. Statistical analysis was performed using R 3.6.3. False discovery rate method (FDR) was applied to correct the data due to the problem of multiple comparisons. Differences were considered statistically significant only if the *q-*value of FDR was less than 0.05.

## Results

### Instrumental Variables Selection

**Supplementary Table S1** showed the data sources. Initially, we extracted 152 SNPs for chronotype, 78 for sleep duration, 27 for short sleep duration, 8 for long sleep duration, 228 for insomnia, and 37 for daytime sleepiness, respectively (**Supplementary Tables S2–S7**). The *F*-statistic of IVs was greater than 10, where the *F* value for chronotype, short sleep duration, long sleep duration, insomnia, and daytime sleepiness were 59.25, 46.25, 42.90, 45.45, 51.73, and 47.13, respectively. This indicated that there are no weak instrumental variables. The variances explained by these IVs were chronotype 1.33%, sleep duration 0.57%, short sleep duration 0.22%, long sleep duration 0.08%, insomnia 0.48%, and daytime sleepiness 0.31%. To analyze the effect of SLE (exposure) on the risk of sleep traits (outcomes), we incorporated 43 SNPs (*P* < 5 × 10^-8^) with LD removed as IVs for SLE (**Supplementary Table S8**). The genetic strength was sufficient with an *F*-statistic of 114.77 for both and 16.19% of the variance explained. **Supplementary Table S9** showed the details of the instrument variables, including Beta, SE, and *P* value.

### Chronotype and SLE

**Figure 1:**
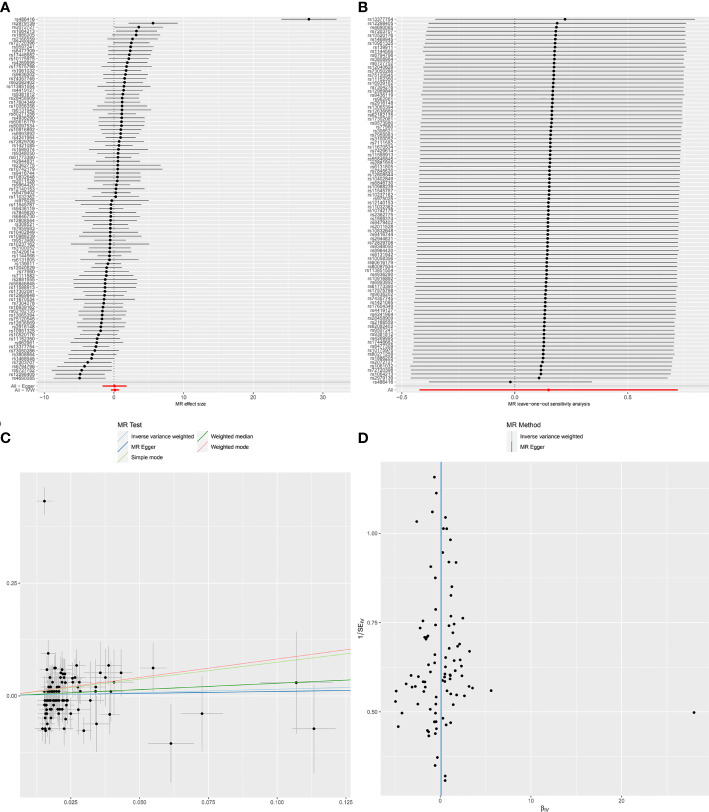
Forest plot **(A)**, sensitivity analysis **(B)**, scatter plot **(C)** and funnel plot **(D)** of the causal effect of Chronotype on SLE risk.

Initially, we extracted 152 genome-wide significant (*P* < 5 × 10 ^− 8^) SNPs previously demonstrated to be associated with chronotype, due to LD with other variants, 37 SNPs were eliminated. Next, when extracting the information of IVs in the outcome, 9 SNPs were excluded because there was no corresponding outcome. When harmonizing exposure data and outcome data, 14 SNPs were excluded because they were palindromic SNPs. Finally, a total of 92 SNPs were included for MR analysis. The results of IVW model suggested that chronotype was not associated with SLE risk (*OR* = 1.20, 95% *CI*, 0.68-2.12, *q-*value = 0.747) (**Table 1** and **Figure 1**). The Weighted median MR estimations revealed that chronotype (*OR*=1.51, 95%*CI*=0.94-2.43, *q-*value = 0.274) was unrelated to the risk of SLE. In addition, the results of MR-Egger (*OR* = 1.04, 95% *CI* = 0.19-5.71, *q-*value = 0.963) and Weighted mode (*OR* = 2.15, 95% *CI* = 0.82-5.68, *q-*value = 0.124) were consistent with Weighted mode.

**Table 1:** MR analysis for the causality of sleep traits with the risk of SLE.

Exposure/Outcome	Nsnp	Methods	*OR* (95%*CI*)	*SE*	*P* value	q-value	Horizontal pleiotropy							Heterogeneity			
							MR-Egger regression			MR-PRESSO				Cochran’s Q	P value	P* value	*F*
							Egger intercept	*SE*	P value	Global test P value	Outliers	β* (95% *CI*)	q* -value				
Chronotype/SLE	92	IVW	1.20 (0.68-2.12)	0.29	0.526	0.747	3.41E-03	0.02	0.861	<0.001	rs486416, rs13377754	0.09 (-0.27-0.44)	0.847	317.11	<0.001	0.026	59.25
		MR Egger	1.04 (0.19-5.71)	0.87	0.963	0.963						0.77 (0.27-1.82)	0.608				
		Weighted median	1.51 (0.94-2.43)	0.24	0.087	0.274						0.48 (0.02-0.95)	0.168				
		Weighted mode	2.15 (0.82-5.68)	0.49	0.124	0.124						0.78 (-0.14-1.70)	0.400				
Sleep duration/SLE	55	IVW	1.01 (0.99-1.03)	0.01	0.399	0.747	7.42E-05	0.02	0.997	<0.001	rs1991556, rs34556183	0.01 (-0.01-0.03)	0.338	117.10	<0.001	0.021	46.25
		MR Egger	1.01 (0.95-1.07)	0.03	0.752	0.963						0.02 (-0.03-0.07)	0.731				
		Weighted median	0.99 (0.96-1.01)	0.01	0.255	0.382						-0.01 (-0.04-0.01)	0.360				
		Weighted mode	0.99 (0.96-1.02)	0.02	0.510	0.510						-0.01 (-0.05-0.02)	0.473				
Short sleep duration/SLE	21	IVW	1.01 (0.96-1.06)	0.03	0.712	0.747	0.10	0.04	0.045	0.003	–	–	–	45.87	<0.001	–	42.90
		MR Egger	0.80 (0.65-0.99)	0.11	0.059	0.354						–	–				
		Weighted median	1.03 (0.97-1.08)	0.03	0.356	0.427						–	–				
		Weighted mode	1.03 (0.95-1.13)	0.04	0.487	0.487						–	–				
Long sleep duration/SLE	6	IVW	0.91 (0.83-0.99)	0.05	0.037	0.222	0.01	0.05	0.051	0.030	rs17688916	-0.10 (-0.19–0.005)	0.148	1.04	0.960	0.960	45.45
		MR Egger	0.89 (0.68-1.16)	0.14	0.441	0.882						-0.12 (-0.38-0.15)	0.731				
		Weighted median	0.92 (0.82-1.02)	0.05	0.111	0.274						-0.09 (-0.20-0.02)	0.242				
		Weighted mode	0.94 (0.81-1.08)	0.07	0.409	0.409						-0.07 (-0.22-0.08)	0.473				
Insomnia/SLE	125	IVW	1.02 (0.88-1.19)	0.08	0.747	0.747	0.01	0.01	0.275	0.031	–	–	–	156.09	0.027	–	51.73
		MR Egger	0.76 (0.44-1.32)	0.28	0.335	0.882						–	–				
		Weighted median	1.17 (0.95-1.45)	0.11	0.137	0.274						–	–				
		Weighted mode	1.41 (0.84-2.37)	0.26	0.192	0.192						–	–				
Daytime sleepiness/SLE	30	IVW	0.66 (0.05-7.95)	1.27	0.742	0.747	-0.01	0.04	0.899	<0.001	rs6923811	0.36 (-1.60-2.32)	0.994	73.16	<0.001	0.742	47.13
		MR Egger	1.39 (1.10e-05-1.76e+05)	5.99	0.956	0.963						-0.80 (-9.91-8.32)	0.814				
		Weighted median	1,63 (0.14-18.37)	1.24	0.694	0.694						1.12 (-1.28-3.51)	0.718				
		Weighted mode	0.15 (0.002-9.93)	2.18	0.389	0.389						-1.16 (-5.34-3.03)	0.473				

*The result of recalculation after removing outliers.

MR-PRESSO, MR-Pleiotropy Residual Sum and Outlier method. OR, odds ratio; CI, confidence interval; IVW, inverse-variance weighted.

q-value, P-value corrected for False Discovery Rate.

MR-Egger regression test (intercept *P*=0.861) did not support any evidence for directional pleiotropy. The symmetry of the funnel plot also indicated the same result (**Figure 1D**). However, MR-PRESSO detected rs486416 and rs13377754 as outliers, revealing that the IVs of chronotype and outcome had a substantial horizontal pleiotropy (*P* < 0.001). Results did not change substantially after removal of outliers (IVW, *β* *=0.09, 95%*CI* = -0.27-0.44, q*-value = 0.847). Heterogeneity test results showed heterogeneity between SNP effect estimates. After removing the outliers detected in MR-PRESSO, there was still marked heterogeneity among SNPs.

**Figure 2:**
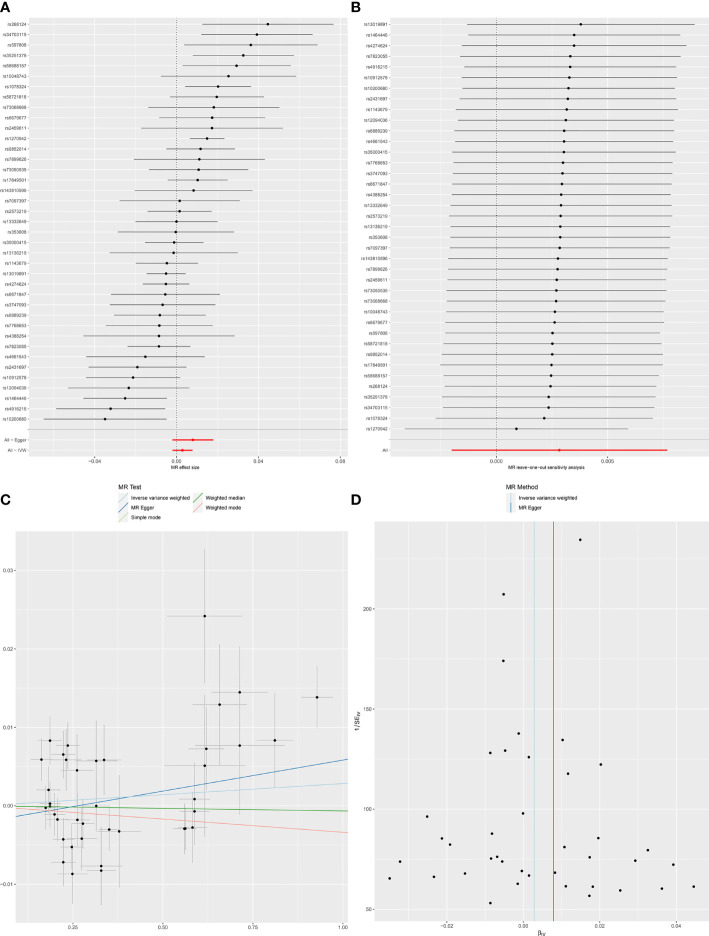
Forest plot **(A)**, sensitivity analysis **(B)**, scatter plot **(C)** and funnel plot **(D)** of the causal effect of SLE on Chronotype risk.

When SLE was included as an exposure and chronotype as an outcome, the IVW model results indicated that SLE was not associated with the risk of chronotype (*OR* = 1.00, 95% *CI*, 0.99-1,01, *q-*value = 0.756). These results remained consistent when using MR-Egger, Weighted median, and Weighted mode. Heterogeneity between individual SNP estimates was detected by Cochran’s *Q* test(*Q* = 92.50, *P* =1.93E-06). MR-Egger regression did not detect horizontal pleiotropy, however, MR-PRESSO analysis detected rs 1270942 as an outlier. After removal of this outlier, the calculation was repeated, and it was found that the results did not change and the heterogeneity also remained (**Table 2** and **Figure 2**). The results in the above two directions indicated that, on the one hand, there was no causal relationship between chronotype and SLE risk, and on the other hand, there was also no causal relationship between SLE and chronotype risk.

**Table 2:** MR analysis of the causal relationship between SLE and the risk of sleep traits.

Exposure/Outcome	Nsnp	Methods	*OR*(95%*CI*)	*SE*	*P* value	*q*-value	Horizontal pleiotropy							Heterogeneity			
							MR-Egger regression			MR-PRESSO				Cochran’s *Q*	*P* value	P* value	*F*
							Egger intercept	*SE*	*P* value	Global test *P* value	Outliers	β* (95% CI)	q* -value				
SLE/Chronotype	39	IVW	1.00(0.99-1.01)	2.47E-03	0.252	0.756	-0.002	0.002	0.260	<0.001	rs1270942	0.001(-0.004-0.006)	0.726	92.50	1.93E-06	2.07E-05	114.77
		MR Egger	1.01(0.99-1.02)	5.08E-03	0.128	0.384						0.003(-0.008-0.014)	0.615				
		Weighted median	1.00(0.99-1.00)	2.80E-03	0.815	0.815						-0.004(-0.010-0.001)	0.213				
		Weighted mode	1.00(0.99-1.01)	4.83E-03	0.489	0.684						-0.004(-0.01-0.003)	0.335				
SLE/Sleep duration	39	IVW	1.00(0.99-1.00)	1.87E-03	0.517	0.796	5.61E-04	0.001	0.690	<0.001	–	–		74.16	4.027E-04	–	114.77
		MR Egger	1.00(0.99-1.01)	3.89E-03	0.967	0.967						–					
		Weighted median	1.00(0.99-1.00)	2.38E-03	0.805	0.815						–					
		Weighted mode	1.00(0.99-1.00)	4.21E-03	0.383	0.684						–					
SLE/Short sleep duration	39	IVW	1.00(0.99-1.00)	7.52E-04	0.645	0.796	-2.83E-04	5.61E-04	0.618	0.005	rs3747093	0.001(-0.001-0.002)	0.726	67.07	0.002	0.014	114.77
		MR Egger	1.00(0.99-1.00)	1.57E-03	0.512	0.614						0.001(-0.002-0.004)	0.615				
		Weighted median	1.00(0.99-1.00)	9.45E-04	0.140	0.420						0.001(0.000-0.003)	0.213				
		Weighted mode	1.00(0.99-1.00)	1.26E-03	0.244	0.684						0.002(0.000-0.004)	0.335				
SLE/Long sleep duration	39	IVW	1.00(0.99-1.00)	4.34E-04	0.051	0.306	7.87E-06	3.25E-04	0.981	0.402	–	–		38.97	0.426	–	114.77
		MR Egger	1.00(0.99-1.00)	9.07E-04	0.367	0.550						–					
		Weighted median	1.00(0.99-1.00)	6.29E-04	0.289	0.578						–					
		Weighted mode	1.00(0.99-1.00)	7.94E-04	0.570	0.684						–					
SLE/Insomnia	39	IVW	1.00(0.99-1.00)	1.20E-03	0.736	0.796	9.55E-04	8.83E-04	0.287	<0.001	rs6671847	-0.001(-0.003-0.001)	0.726	81.88	4.69E-05	0.001	114.77
		MR Egger	1.00(0.99-1.00)	2.47E-03	0.274	0.548						-0.002(-0.006-0.003)	0.615				
		Weighted median	1.00(0.99-1.00)	1.32E-03	0.651	0.815						-0.001(-0.003-0.002)	0.619				
		Weighted mode	1.00(0.99-1.00)	1.64E-03	0.750	0.750						-0.001(-0.004-0.003)	0.739				
SLE/Daytime sleepiness	39	IVW	1.00(0.99-1.00)	8.22E-04	0.796	0.796	9.73E-04	5.94E-04	0.110	0.002	rs1143679, rs6679677	0.000(-0.001-0.002)	0.726	69.59	0.001	0.004	114.77
		MR Egger	1.00(0.99-1.00)	1.66E-03	0.127	0.384						0.001(-0.002-0.004)	0.615				
		Weighted median	1.00(0.99-1.00)	1.01E-03	0.105	0.420						0.001(-0.001-0.003)	0.213				
		Weighted mode	1.00(0.99-1.00)	1.20E-03	0.066	0.396						0.002(-0.001-0.004)	0.335				

*The result of recalculation after removing outliers.

MR-PRESSO, MR-Pleiotropy Residual Sum and Outlier method. OR, odds ratio; CI, confidence interval; IVW, inverse-variance weighted.

q-value, P-value corrected for False Discovery Rate.

### Sleep Duration and SLE

We excluded 15 SNPs for sleep duration, 2 SNPs for short sleep duration, and 1 SNP for long sleep duration after clumping for the selected SNPs. Due to no corresponding outcomes in sleep duration, we eliminated rs17732997 and rs1607227 from the sleep duration and short sleep duration instrument sets, respectively. And seven SNPs (rs10421649, rs11643715, rs12791153, rs2079070, rs269054, rs915416, rs994064) for sleep duration, three SNPs (rs2186122, rs60882754, rs9367621) for short sleep duration and one SNP (rs17688916) for long sleep duration were removed for being palindromic. Therefore, 55 SNPs (including 7 proxy SNPs) for sleep duration, 21 SNPs (including 1 proxy SNP) for short sleep duration, and 6 SNPs (including 2 proxy SNPs) for long sleep duration were finally incorporated in the MR analysis. According to the results of two-sample MR analysis, neither sleep duration (IVW, *OR* = 1.01,95%*CI* = 0.99-1.03, *q-*value = 0.747) nor short sleep duration (IVW, *OR* = 1.01,95%*CI* = 0.96-1.06, *q-*value = 0.747) were shown to be causally linked to SLE (**Table 1** and **Supplementary Figures S1, S2**). Similarly, long sleep duration (IVW, *OR* = 0.91, 95%*CI* = 0.83-0.99, *q*-value = 0.222) did not have a causal relationship with SLE risk (**Supplementary Figure S3**).

The horizontal pleiotropy in sleep duration was not found using the MR-Egger regression test (Egger intercept =7.42E-05, *P* = 0.997). Rs1991556 and rs34556183 in the sleep duration were removed as outliers in MR-PRESSO global tests, but the results did not change significantly. In short sleep duration, both the MR-Egger regression and the MR-PRESSO test demonstrated horizontal pleiotropy, although no outliers were found. Both sleep duration and short sleep duration were detected for the presence of heterogeneity. In the analysis of long sleep duration, no significant heterogeneity was seen in the Cochrane *Q* statistic (*P* = 0.960), and MR-Egger regression results showed no horizontal pleiotropy (*P* = 0.051). However, MR-PRESSO revealed considerable level of horizontal pleiotropy (*P* = 0.030) and detected rs17688916 as a pleiotropic SNP.

Setting SLE to exposure, based on IVW analysis, revealed no evidence of a causal relationship between SLE and sleep duration (*OR* = 1.00, 95%*CI* = 0.99-1.00, *q*-value = 0.796), short sleep duration (*OR* = 1.00, 95%*CI* = 0.99-1.00, *q*-value = 0.796) as well as long sleep duration (*OR* = 1.00, 95%*CI* = 0.99-1.00, *q*-value = 0.306). When Cochran’s *Q* was used to test heterogeneity, heterogeneity was detected in the analysis of SLE with sleep duration (*Q* = 74.16, *P* = 4.027E-04) and in the analysis of SLE and short sleep duration (*Q* = 67.07, *P* = 0.002). In all of the analyses, the MR-Egger regression approach failed to detect the presence of horizontal pleiotropy (**Table 2** and **Supplementary Figures S6–S8**).

### Insomnia and SLE

In our MR analysis, we selected 228 SNPs that are strongly associated with insomnia. Among the genetic variants, 73 SNPs were ruled out after the clumping process, 7 SNPs had no corresponding outcome, and 23 SNPs were excluded because they were palindromic SNPs. Finally, MR analysis was performed on 125 SNPs. With the IVW analysis, there was no evidence of a causal relationship between genetically determined insomnia and SLE (*OR* =1.02, 95%*CI* = 0.88-1.19, *q-*value = 0.747), and the other three methods were consistent with this result (**Table 1**). The MR-Egger regression test indicated no horizontal pleiotropy (intercept *P* = 0.275) when the MR model was restricted to the remaining 125 instruments with 7 proxy SNPs. According to the MR-PRESSO test, the results were the opposite, but there were no outliers. The Cochrane *Q* test indicated strong heterogeneity (*Q*=156.09, *P* = 0.027), which was consistent with the funnel plot results (**Supplementary Figure S4**). The results of IVW method revealed no evidence of a causal relationship between genetically determined insomnia and SLE (*OR* = 1.02, 95%*CI* = 0.88 - 1.19, *P* = 0.747), and the other methods were consistent with this finding (**Table 1**).

In the analysis of the effect of SLE on insomnia risk, there was no evidence of an effect of SLE on insomnia risk based on the IVW method (*OR* = 1.00, 95%*CI* = 0.99-1.00, *q-*value = 0.796) (**Table 2**). This finding is consistent in the other three MR estimates. In the MR-Egger regression analysis, it was shown that there was no pleiotropy of effect was shown between SLE and insomia (intercept *P* = 0.287). But the MR-PRESSO test showed a pleiotropy between SLE and insomnia and detected rs 6671847 as an outlier. After removing this SNP (rs6671847) the results still showed no causal relationship between SLE and insomnia risk (β* = -0.001, 95%*CI* = -0.003-0.001, q*-value = 0.726). Whether or not outliers were excluded, significant heterogeneity was observed between SLE and insomnia (**Table 2** and **Supplementary Figure S9**).

### Daytime Sleepiness and SLE

In the study of the effect of daytime sleepiness on SLE risk, 37 SNPs were selected as instruments. After removing SNPs with LD with other variables, SNPs without corresponding outcomes, palindromic SNPs, as well as SNPs with incompatible alleles, only a total of 30 SNPs remained for MR analysis. In none of the MR estimations, there was any indication of a causal association between daytime sleepiness and SLE. (IVW, *OR* = 0.66, 95%*CI =*0.05-7.95, *q-*value = 0.747) (**Table 1**). Horizontal pleiotropy was not detected using MR-Egger intercept testing (intercept *P* = 0.899). There was obvious heterogeneity for daytime sleepiness (*Q* = 73.16, *P* < 0.001) (**Supplementary Figure S5**), but when the outlier SNP (rs6923811) was trimmed, the heterogeneity disappeared.

In the study in the reverse direction (SLE on the risk of daytime sleepiness), the *OR* (95%*CI*) of daytime sleepiness calculated using IVW, MR Egger, Weighted median and Weighted mode analysis were 1.00 (0.99-1.00, *q*-value = 0.796), 1.00 (0.99-1.00, *q*-value = 0.384), 1.00 (0.99-1.00, *q*-value = 0.420) and 1.00 (0.99-1.00, *q*-value = 0.396), respectively, and no genetic effect of SLE on the risk of daytime sleepiness was found. MR-Egger regression analysis did not indicate that IVs had horizontal pleiotropy (intercept *P =* 0.110). Cochran’s *Q* test reported the presence of heterogeneity (*Q* = 69.59, *P* = 0.001) (**Table 2** and **Supplementary Figure S10**).

### Potential Pleiotropy Searched in PhenoScanner

In total, 329 SNPs were used for the main MR analysis in the study of the effect of sleep traits on SLE risk. We searched these SNPs in turn in the PhenoScanner database and found five main traits that may undergo potential pleiotropy: “alcohol intake frequency”, “average weekly beer plus cider intake”, “current tobacco smoking”, “ever smoked” and “past smoked smoking” (**Supplementary Table S10**). After removing these involved SNPs, the sensitivity analysis was re-conducted and the results were found to be consistent with previous ones (**Supplementary Table S11** and **Supplementary Figures S11–S15**). The results of studies on the direction of the effect of SLE on the risk of sleep-related traits can be found in Supplementary materials (**Supplementary Tables S10, S11**) (**Supplementary Figures S16–S21**).

## Discussion

A bidirectional hypothesized causal relationship between sleep traits and SLE was explored in this bidirectional TSMR study. According to our findings, there was no evidence of a causal relationship between chronotype, sleep duration, short sleep duration, long sleep duration, insomnia, daytime sleepiness, and the risk of SLE. Similarly, our findings did not support a causal relationship between genetic susceptibility to SLE and sleep traits.

This study yielded conflicting results with previous observational studies reporting an association between sleep and SLE. On the one hand, it has been revealed that sleep of less than 7 hours per night was associated with the transition to SLE in family members with SLE (36). A nationwide population-based cohort study 144,396 participants demonstrated that sleep disorders increase the risk of SLE (37). Another cohort study of 84,996 participants indicated that insomnia is a risk factor of SLE (13). On the other hand, poor sleep quality and sleep disturbances have been widely reported in SLE patients (4, 38). This was also confirmed by a previous meta-analysis of our research group (39). At the same time, a case-control study found that women with SLE had decreasing melatonin levels and were inversely proportional to the activity of the disease (40), while melatonin was thought to be effective in improving sleep quality (41). Therefore, sleep quality in SLE patients may be related to disease activity. Taking together, these pieces of evidence support that sleep is responsible for the development and progression of SLE and also support that SLE can affect sleep.

However, our study did not provide evidence that sleep traits play a role in the increased risk of SLE. We considered the interpretation of the results in terms of psychological factors. Previous studies have confirmed a bidirectional relationship between sleep disturbance and depression (42), that is, sleep disorders can lead to the development of depression. In addition, the results of a 20-year cohort study showed that depression may increase the risk of SLE (43). This suggested that sleep disturbance may affect SLE through depression. In SLE patients, however, there was a strong relationship between sleep and fatigue, pain, depression, and anxiety (4, 44). Therefore, there may be some interaction between these factors in the mechanism of the association between sleep and SLE. This implies that sleep may not be directly associated with an increased risk of SLE. Therefore, sleep may act indirectly on SLE through factors such as depression. In addition, a cohort study in Taiwan found that OSA was not associated with an increased risk of SLE (14). Therefore, while most studies demonstrated that sleep disorders were common in SLE populations, sleep disorders may simply be one of the clinical symptoms of SLE and there was no causal relationship between them.

We also found no evidence of a causal relationship between SLE and sleep traits. Poor sleep quality in SLE patients may result from a combination of factors rather than from the disease itself. Studies have reported that depressed mood, lack of exercise as well as the use of prednisone drugs can make SLE patients sleep poorly (45). Prednisone, on the other hand, was a drug for long-term maintenance therapy in SLE patients (46). As a result, the usage of medications after the disease may be causing the generation of sleep problems in SLE patients. In addition, the prevalence of depression and anxiety was relatively prevalent in SLE patients (47). And people with depression and anxiety disorders will have more insomnia compared to normal people (48). Therefore, poor sleep quality in SLE patients may be associated with depression and anxiety.

The SNPs used for analysis were manually searched in the PhenoScanner database, and five traits that may develop potential pleiotropy, “alcohol intake frequency”, “averageweekly beer plus cider intake”, “current tobacco smoking”, “ever smoked” and “past smoked smoking”, were detected. Because the two characteristics of smoking and alcohol consumption have already been shown to be common predictors of sleep and SLE in previous studies (49–52) and they are not intermediate variables in the causation of sleep and SLE. As a result, in this study, smoking and alcohol consumption were employed as confounders for exposure outcomes. Then, after controlling for this potential pleiotropy, the results were found not to be altered, possibly due to the small number of SNPs with potential pleiotropy that had less impact on the results.

This study had the following strengths. First, to the best of our knowledge, this study was the first MR analysis of sleep traits with SLE and explored the causal relationship between a range of sleep-related traits and SLE, and to conduct a study of bidirectional causal relationship, significantly extending the relevant studies. Second, in contrast to previous observational studies that found sleep to be associated with SLE, MR design studies were not vulnerable to confounding factors. Third, we took a number of steps to meet the core assumptions of MR. Specifically, the IVs for sleep characteristics were derived from large-scale GWASs, which provided strong and reliable association genome-wide associated SNPs and avoided potential weak instrumental bias. In addition, the MR-PRESSO method was used to detect and correct for deviations arising from horizontal pleiotropy. Finally, the study used a large sample size and SNPs from GWASs, which gave it enough statistical validity to estimate causality. These metrics bolster the credibility of the findings.

However, our study also has some limitations. First, because the participants in the dataset we used were exclusively of European ancestry. Therefore, our study may have some participants overlap, while overlapping samples in the two-sample MR analysis may lead to an overestimation of the results. At the same time, whether our results can be extrapolated to other ethnic populations also requires further investigation. Second, our study could not verify whether there was dose–response relationships between sleep traits and SLE. Third, the sleep in the present study data we used were obtained from a self-reported questionnaire rather than objectively measured, which the potential bias may arise. Finally, our study did not conduct further subgroup analysis.

Overall, our study of bidirectional TSMR had neither evidence to support a protective or deleterious effect of genetically predicted sleep traits on SLE nor evidence to support an effect of genetically determined SLE on sleep-related traits. As a result, further large-scale or longitudinal studies are required to investigate the causal relationship between sleep and SLE. In addition, the latest data from large-scale genetic studies can also be used for further studies. At the same time, this study also emphasizes the necessity of further studying the mechanism of the relationship between sleep and SLE.

## Data Availability Statement

The original contributions presented in the study are included in the article/**Supplementary Material**. Further inquiries can be directed to the corresponding author.

## Author Contributions

G-CW conceived and presented the idea. NS and R-CG processed data and manuscript writing. M-YZ, Z-ZW and Z-GW participated in the acquisition and interpretation of data. All authors contributed to the article and approved the submitted version.

## Funding

This study was supported by the Research Fund of Anhui Institute of translational medicine (2021zhyx-C28).

## Conflict of Interest

The authors declare that the research was conducted in the absence of any commercial or financial relationships that could be construed as a potential conflict of interest.

## Publisher’s Note

All claims expressed in this article are solely those of the authors and do not necessarily represent those of their affiliated organizations, or those of the publisher, the editors and the reviewers. Any product that may be evaluated in this article, or claim that may be made by its manufacturer, is not guaranteed or endorsed by the publisher.
