# Supplementary material for: Causal Relationship Between Sleep Traits and Risk of Systemic Lupus Erythematosus: A Two-Sample Mendelian Randomization Study
Source: Front Immunol. 2022 Jun 17;13:918749. doi: 10.3389/fimmu.2022.918749 (PMC9248809; doi:10.3389/fimmu.2022.918749)
Supplement: Supplementary file 1 [file DataSheet_1.docx]

**Supplementary materials**

**Supplementary Table 1.** Summary of genome-wide association studies (GWAS) datasets in our study.

| **Phenotype** | **Type of trait** | **Author, published year** | **Consortium** | **Sample size** | **No. of cases (Binary trait)** | **PMID** |
| --- | --- | --- | --- | --- | --- | --- |
| Chronotype | Binary | Jones SE et al, 2019 | UKB | 403,195 | 252,287 | 30696823 |
| Sleep duration | Continuous | Dashti HS et al, 2019 | UKB | 446,118 | NA | 30846698 |
| Short sleep duration | Binary | Dashti HS et al, 2019 | UKB | 411,934 | 106,192 | 30846698 |
| Long sleep duration | Binary | Dashti HS et al, 2019 | UKB | 339,926 | 34,184 | 30846698 |
| Insomnia | Binary | Jansen PR et al, 2019 | 23andMe and UKB | 1,331,010 | 288,557 | 30804565 |
| Daytime sleepiness | Ordinal | Wang H et al, 2019 | UKB | 452,071 | NA | 31409809 |
| Systemic Lupus Erythematosus | Binary | Bentham J et al, 2015 | UKB | 23,210 | 7219 | 26502338 |

**Supplementary Table 2.** The summary information for instrumental variables of chronotype.

| **Sort** | **Phenotype** | **SNP** | **effect_allele** | **other_allele** | **beta** | **se** | **pval** |
| --- | --- | --- | --- | --- | --- | --- | --- |
| 1 | Chronotype | rs1144566 | T | C | 0.113367 | 0.00783578 | 5.80E-47 |
| 2 | Chronotype | rs2653349 | A | G | 0.0387249 | 0.00327448 | 5.20E-32 |
| 3 | Chronotype | rs80271258 | T | C | -0.0549164 | 0.00476547 | 2.60E-30 |
| 4 | Chronotype | rs12040629 | A | G | 0.0391561 | 0.00364815 | 6.70E-27 |
| 5 | Chronotype | rs13377754 | T | C | 0.0297291 | 0.00275916 | 6.90E-27 |
| 6 | Chronotype | rs28458909 | T | C | -0.0432803 | 0.00407749 | 2.60E-26 |
| 7 | Chronotype | rs1843888 | A | G | 0.0288388 | 0.00270621 | 3.20E-26 |
| 8 | Chronotype | rs4729854 | A | T | -0.0287186 | 0.00275036 | 3.30E-25 |
| 9 | Chronotype | rs61773390 | T | G | 0.0340792 | 0.00337553 | 1.20E-23 |
| 10 | Chronotype | rs1421085 | T | C | -0.0275621 | 0.00274075 | 1.50E-23 |
| 11 | Chronotype | rs9573980 | A | G | 0.0727085 | 0.00745314 | 5.70E-22 |
| 12 | Chronotype | rs12927162 | A | G | 0.0286498 | 0.00300067 | 6.80E-22 |
| 13 | Chronotype | rs9964420 | A | C | -0.0284801 | 0.00293643 | 8.60E-22 |
| 14 | Chronotype | rs2550298 | T | C | -0.0239989 | 0.0027767 | 3.60E-18 |
| 15 | Chronotype | rs9436119 | A | G | 0.0234899 | 0.00273835 | 4.00E-18 |
| 16 | Chronotype | rs72720396 | A | G | -0.0271261 | 0.00318521 | 8.40E-18 |
| 17 | Chronotype | rs10520176 | T | C | 0.0231498 | 0.00268932 | 1.40E-17 |
| 18 | Chronotype | rs139911 | T | C | -0.0233915 | 0.00273231 | 2.30E-17 |
| 19 | Chronotype | rs11032362 | A | G | 0.0395538 | 0.00467579 | 4.80E-17 |
| 20 | Chronotype | rs1061032 | T | G | 0.0387677 | 0.00477391 | 2.00E-16 |
| 21 | Chronotype | rs9479402 | T | C | -0.106941 | 0.0131937 | 3.20E-16 |
| 22 | Chronotype | rs12969848 | T | C | 0.0218632 | 0.00270301 | 1.30E-15 |
| 23 | Chronotype | rs67988891 | C | G | -0.0232401 | 0.00288939 | 3.70E-15 |
| 24 | Chronotype | rs9381812 | A | G | -0.023054 | 0.00294635 | 4.60E-15 |
| 25 | Chronotype | rs6967481 | T | C | 0.0206433 | 0.00269925 | 6.90E-15 |
| 26 | Chronotype | rs4241964 | T | G | -0.020735 | 0.00270449 | 1.10E-14 |
| 27 | Chronotype | rs4419127 | A | G | 0.0218058 | 0.00285599 | 1.30E-14 |
| 28 | Chronotype | rs62082402 | T | G | 0.0256161 | 0.00342345 | 1.60E-14 |
| 29 | Chronotype | rs11545787 | A | G | -0.0238098 | 0.0031377 | 4.90E-14 |
| 30 | Chronotype | rs10951325 | T | C | 0.0205969 | 0.00280238 | 7.90E-14 |
| 31 | Chronotype | rs6993892 | T | C | -0.0205162 | 0.00275662 | 8.40E-14 |
| 32 | Chronotype | rs75120545 | T | C | 0.0613917 | 0.00828587 | 1.10E-13 |
| 33 | Chronotype | rs12140153 | T | G | -0.0340109 | 0.00468803 | 1.60E-13 |
| 34 | Chronotype | rs17604349 | A | G | -0.0257422 | 0.00351443 | 1.70E-13 |
| 35 | Chronotype | rs28380327 | A | T | 0.0205097 | 0.00277698 | 2.90E-13 |
| 36 | Chronotype | rs308521 | T | C | 0.0199718 | 0.00275427 | 3.70E-13 |
| 37 | Chronotype | rs2737245 | T | G | 0.0215069 | 0.00300138 | 4.50E-13 |
| 38 | Chronotype | rs10916892 | T | C | -0.0199965 | 0.00278123 | 6.10E-13 |
| 39 | Chronotype | rs812925 | C | G | -0.0205641 | 0.00280786 | 6.90E-13 |
| 40 | Chronotype | rs7959983 | T | C | -0.0195258 | 0.00272849 | 9.50E-13 |
| 41 | Chronotype | rs13011556 | C | G | -0.0227424 | 0.00316075 | 1.10E-12 |
| 42 | Chronotype | rs10254050 | C | G | -0.0246317 | 0.00344351 | 1.20E-12 |
| 43 | Chronotype | rs1064213 | A | G | 0.019141 | 0.00268602 | 1.30E-12 |
| 44 | Chronotype | rs8044054 | T | C | 0.019218 | 0.00275723 | 1.50E-12 |
| 45 | Chronotype | rs17448682 | T | C | 0.0221528 | 0.00318247 | 2.20E-12 |
| 46 | Chronotype | rs16939162 | A | G | 0.0249887 | 0.00358356 | 2.20E-12 |
| 47 | Chronotype | rs1468945 | A | G | -0.0225859 | 0.00326992 | 4.20E-12 |
| 48 | Chronotype | rs6846730 | T | C | -0.0220017 | 0.00317897 | 5.60E-12 |
| 49 | Chronotype | rs12636669 | T | C | 0.0343448 | 0.00497499 | 5.80E-12 |
| 50 | Chronotype | rs4269995 | T | C | -0.021271 | 0.00309061 | 5.90E-12 |
| 51 | Chronotype | rs62553781 | T | C | -0.0504004 | 0.00734656 | 1.20E-11 |
| 52 | Chronotype | rs1013987 | T | C | -0.0182666 | 0.00274466 | 1.50E-11 |
| 53 | Chronotype | rs10988239 | T | C | -0.0186334 | 0.00273304 | 1.60E-11 |
| 54 | Chronotype | rs7304278 | A | G | -0.020255 | 0.00302264 | 2.20E-11 |
| 55 | Chronotype | rs2362775 | T | C | -0.0180186 | 0.0027019 | 2.80E-11 |
| 56 | Chronotype | rs12808544 | A | C | -0.0207525 | 0.00314754 | 3.00E-11 |
| 57 | Chronotype | rs17575798 | A | G | -0.0225553 | 0.00339377 | 4.20E-11 |
| 58 | Chronotype | rs77960 | A | G | 0.0187567 | 0.00286325 | 4.20E-11 |
| 59 | Chronotype | rs72796401 | A | T | 0.0219581 | 0.00343008 | 5.90E-11 |
| 60 | Chronotype | rs247929 | C | G | 0.0177077 | 0.0026901 | 6.20E-11 |
| 61 | Chronotype | rs621421 | T | C | -0.0177395 | 0.00277678 | 7.50E-11 |
| 62 | Chronotype | rs184033703 | A | G | 0.0377937 | 0.00576165 | 8.10E-11 |
| 63 | Chronotype | rs74357745 | A | G | 0.026546 | 0.00412156 | 8.70E-11 |
| 64 | Chronotype | rs6131942 | A | G | -0.0175437 | 0.00273235 | 9.40E-11 |
| 65 | Chronotype | rs60616179 | A | G | 0.0375809 | 0.00594669 | 1.10E-10 |
| 66 | Chronotype | rs975025 | T | C | -0.0321062 | 0.00502525 | 1.30E-10 |
| 67 | Chronotype | rs10175975 | T | C | 0.0228441 | 0.00351376 | 1.80E-10 |
| 68 | Chronotype | rs9817910 | A | G | -0.0170371 | 0.00270846 | 3.10E-10 |
| 69 | Chronotype | rs114848860 | A | T | -0.0555837 | 0.00872612 | 3.60E-10 |
| 70 | Chronotype | rs62182135 | A | C | -0.0179439 | 0.00284795 | 3.70E-10 |
| 71 | Chronotype | rs4936290 | A | C | -0.0177058 | 0.00282752 | 4.60E-10 |
| 72 | Chronotype | rs1886205 | A | C | 0.0194352 | 0.00316104 | 4.90E-10 |
| 73 | Chronotype | rs7845620 | A | C | -0.0224873 | 0.00363166 | 5.20E-10 |
| 74 | Chronotype | rs7111582 | A | G | -0.0277534 | 0.00442599 | 5.20E-10 |
| 75 | Chronotype | rs10917513 | T | C | -0.0179024 | 0.00282417 | 6.70E-10 |
| 76 | Chronotype | rs3850174 | A | T | -0.0194471 | 0.00309938 | 6.80E-10 |
| 77 | Chronotype | rs3100052 | A | G | 0.0170795 | 0.00276058 | 6.90E-10 |
| 78 | Chronotype | rs2979139 | A | G | -0.0168003 | 0.00269315 | 7.40E-10 |
| 79 | Chronotype | rs9348050 | T | C | 0.016638 | 0.00269048 | 7.50E-10 |
| 80 | Chronotype | rs9597241 | A | C | 0.0213884 | 0.00345609 | 7.50E-10 |
| 81 | Chronotype | rs9636202 | A | G | -0.0183734 | 0.00305534 | 7.50E-10 |
| 82 | Chronotype | rs7203707 | A | C | -0.0164726 | 0.00269903 | 7.60E-10 |
| 83 | Chronotype | rs6477309 | T | C | 0.0174399 | 0.00286276 | 8.00E-10 |
| 84 | Chronotype | rs149611468 | T | C | 0.0776374 | 0.0125983 | 8.50E-10 |
| 85 | Chronotype | rs2072727 | T | C | 0.016352 | 0.00271514 | 8.50E-10 |
| 86 | Chronotype | rs7429614 | T | G | 0.0164171 | 0.00272259 | 9.20E-10 |
| 87 | Chronotype | rs80097534 | T | G | -0.0281025 | 0.00455453 | 9.30E-10 |
| 88 | Chronotype | rs113851554 | T | G | -0.0357353 | 0.00598996 | 1.00E-09 |
| 89 | Chronotype | rs4672458 | T | C | -0.0162709 | 0.00268601 | 1.10E-09 |
| 90 | Chronotype | rs10818834 | T | C | 0.0188673 | 0.00304915 | 1.10E-09 |
| 91 | Chronotype | rs9416744 | A | C | 0.0189843 | 0.00308075 | 1.30E-09 |
| 92 | Chronotype | rs11152350 | A | C | -0.0163037 | 0.00269517 | 1.30E-09 |
| 93 | Chronotype | rs3857599 | A | C | 0.0218522 | 0.00363894 | 1.40E-09 |
| 94 | Chronotype | rs359248 | T | G | -0.0161518 | 0.00270985 | 1.50E-09 |
| 95 | Chronotype | rs10742179 | A | G | 0.0182967 | 0.00306465 | 1.50E-09 |
| 96 | Chronotype | rs12380242 | T | C | -0.015796 | 0.0026864 | 1.60E-09 |
| 97 | Chronotype | rs11677484 | T | G | 0.0185564 | 0.00309256 | 1.70E-09 |
| 98 | Chronotype | rs12470914 | A | T | 0.0261187 | 0.00443933 | 2.20E-09 |
| 99 | Chronotype | rs55846845 | A | G | -0.016 | 0.00269305 | 2.40E-09 |
| 100 | Chronotype | rs7299922 | A | G | 0.016149 | 0.00279944 | 2.50E-09 |
| 101 | Chronotype | rs13065394 | T | G | -0.017504 | 0.00296404 | 3.00E-09 |
| 102 | Chronotype | rs115073088 | A | G | -0.0524798 | 0.00893134 | 3.10E-09 |
| 103 | Chronotype | rs10832648 | A | C | -0.019805 | 0.00338271 | 3.20E-09 |
| 104 | Chronotype | rs10237162 | T | C | 0.0176177 | 0.00301354 | 3.50E-09 |
| 105 | Chronotype | rs2916148 | A | G | 0.0162149 | 0.0027342 | 3.50E-09 |
| 106 | Chronotype | rs7701529 | A | T | -0.0188448 | 0.00317247 | 3.60E-09 |
| 107 | Chronotype | rs78580841 | T | C | 0.0309392 | 0.0053556 | 3.70E-09 |
| 108 | Chronotype | rs2706762 | T | C | -0.0219218 | 0.00375432 | 4.00E-09 |
| 109 | Chronotype | rs11165655 | A | G | -0.0157164 | 0.00268854 | 4.10E-09 |
| 110 | Chronotype | rs7735794 | A | G | 0.0201147 | 0.00340433 | 4.70E-09 |
| 111 | Chronotype | rs72829706 | A | G | 0.0407112 | 0.00693598 | 5.10E-09 |
| 112 | Chronotype | rs6794796 | A | G | 0.0172176 | 0.00296536 | 5.30E-09 |
| 113 | Chronotype | rs2011528 | T | C | -0.0206881 | 0.00359054 | 5.30E-09 |
| 114 | Chronotype | rs35524253 | A | G | 0.015862 | 0.00281259 | 5.80E-09 |
| 115 | Chronotype | rs10058356 | T | C | -0.0169817 | 0.00293612 | 6.80E-09 |
| 116 | Chronotype | rs17302081 | T | C | 0.0156573 | 0.00270471 | 6.90E-09 |
| 117 | Chronotype | rs848552 | C | G | -0.0153219 | 0.00268579 | 7.00E-09 |
| 118 | Chronotype | rs9347926 | A | T | 0.0157068 | 0.00269893 | 7.30E-09 |
| 119 | Chronotype | rs59986227 | C | G | -0.0178401 | 0.00310217 | 8.30E-09 |
| 120 | Chronotype | rs11670534 | T | C | -0.0209755 | 0.00362802 | 8.50E-09 |
| 121 | Chronotype | rs11611435 | T | C | 0.0157403 | 0.00270297 | 8.60E-09 |
| 122 | Chronotype | rs72841368 | A | T | -0.0197592 | 0.00344327 | 8.70E-09 |
| 123 | Chronotype | rs10402849 | T | C | 0.0192609 | 0.00335946 | 8.90E-09 |
| 124 | Chronotype | rs2166559 | T | C | -0.0218336 | 0.00387088 | 9.10E-09 |
| 125 | Chronotype | rs2944831 | A | G | 0.0172414 | 0.00295094 | 1.10E-08 |
| 126 | Chronotype | rs11641239 | T | C | 0.0172486 | 0.00296753 | 1.10E-08 |
| 127 | Chronotype | rs9394154 | C | G | -0.0155027 | 0.00271526 | 1.30E-08 |
| 128 | Chronotype | rs6727752 | A | G | 0.0156947 | 0.00277464 | 1.40E-08 |
| 129 | Chronotype | rs7943634 | T | C | -0.016455 | 0.00291003 | 1.40E-08 |
| 130 | Chronotype | rs11588913 | A | G | -0.0154525 | 0.00273856 | 1.50E-08 |
| 131 | Chronotype | rs1599374 | A | G | 0.0153807 | 0.00270337 | 1.50E-08 |
| 132 | Chronotype | rs36055559 | A | G | -0.0210912 | 0.00372199 | 1.50E-08 |
| 133 | Chronotype | rs6131805 | T | G | 0.0156349 | 0.00279935 | 1.50E-08 |
| 134 | Chronotype | rs1931814 | A | G | 0.0153877 | 0.00268976 | 1.60E-08 |
| 135 | Chronotype | rs1800828 | C | G | 0.0176235 | 0.00308462 | 1.70E-08 |
| 136 | Chronotype | rs2580160 | A | G | 0.0153499 | 0.00273342 | 1.70E-08 |
| 137 | Chronotype | rs9611597 | A | T | 0.0208731 | 0.00369486 | 1.80E-08 |
| 138 | Chronotype | rs71523448 | C | G | -0.0280253 | 0.00505523 | 2.00E-08 |
| 139 | Chronotype | rs962961 | T | C | -0.0159069 | 0.00286872 | 2.00E-08 |
| 140 | Chronotype | rs4550782 | T | G | 0.015797 | 0.00285144 | 2.20E-08 |
| 141 | Chronotype | rs486416 | A | G | -0.0154284 | 0.00277852 | 2.30E-08 |
| 142 | Chronotype | rs72966564 | T | C | -0.0174371 | 0.00312692 | 2.50E-08 |
| 143 | Chronotype | rs73050286 | T | C | 0.0178095 | 0.00326167 | 2.60E-08 |
| 144 | Chronotype | rs12871550 | A | G | 0.0159966 | 0.00287516 | 2.60E-08 |
| 145 | Chronotype | rs3808964 | T | G | 0.0157096 | 0.00279601 | 2.70E-08 |
| 146 | Chronotype | rs12298405 | T | C | -0.0158538 | 0.00286448 | 2.70E-08 |
| 147 | Chronotype | rs7298532 | T | C | 0.0164304 | 0.00298282 | 2.70E-08 |
| 148 | Chronotype | rs12206814 | C | G | 0.015175 | 0.00273257 | 2.80E-08 |
| 149 | Chronotype | rs7248205 | T | C | 0.0148995 | 0.00275446 | 3.80E-08 |
| 150 | Chronotype | rs4690085 | A | G | -0.0147386 | 0.00269058 | 3.90E-08 |
| 151 | Chronotype | rs295268 | T | C | -0.0165883 | 0.00307939 | 4.10E-08 |
| 152 | Chronotype | rs2881955 | T | C | 0.0161216 | 0.00300229 | 4.30E-08 |

SNP, single nucleotide polymorphism; se, standard error

**Supplementary Table 3.** The summary information for instrumental variables of sleep duration.

| **Sort** | **Phenotype** | **SNP** | **effect_allele** | **other_allele** | **beta** | **se** | **eaf** | **pval** |
| --- | --- | --- | --- | --- | --- | --- | --- | --- |
| 1 | Sleep duration | rs7556815 | A | G | 0.219 | 2.443 | 0.164 | 1.30E-49 |
| 2 | Sleep duration | rs75539574 | C | A | 0.086 | 2.175 | 0.244 | 6.90E-19 |
| 3 | Sleep duration | rs12607679 | T | C | 0.738 | 1.208 | 0.156 | 8.30E-15 |
| 4 | Sleep duration | rs915416 | C | G | 0.29 | 1.156 | 0.15 | 9.90E-15 |
| 5 | Sleep duration | rs9940646 | C | G | 0.578 | 1.017 | 0.137 | 1.20E-13 |
| 6 | Sleep duration | rs13109404 | T | G | 0.928 | 1.872 | 0.264 | 1.40E-12 |
| 7 | Sleep duration | rs8050478 | G | A | 0.5 | 0.96 | 0.136 | 1.70E-12 |
| 8 | Sleep duration | rs62362521 | G | A | 0.334 | 1.017 | 0.144 | 2.20E-12 |
| 9 | Sleep duration | rs13088093 | G | T | 0.336 | 0.976 | 0.144 | 7.00E-12 |
| 10 | Sleep duration | rs2079070 | C | G | 0.265 | 1.053 | 0.154 | 7.50E-12 |
| 11 | Sleep duration | rs34556183 | A | G | 0.72 | 1.015 | 0.151 | 2.30E-11 |
| 12 | Sleep duration | rs3095508 | C | A | 0.594 | 0.921 | 0.138 | 3.10E-11 |
| 13 | Sleep duration | rs34731055 | T | C | 0.181 | 1.168 | 0.177 | 3.70E-11 |
| 14 | Sleep duration | rs73219758 | G | A | 0.708 | 0.984 | 0.15 | 5.60E-11 |
| 15 | Sleep duration | rs10973207 | T | G | 0.158 | 1.226 | 0.187 | 6.00E-11 |
| 16 | Sleep duration | rs2139261 | G | C | 0.749 | 1.122 | 0.174 | 8.50E-11 |
| 17 | Sleep duration | rs4592416 | G | A | 0.464 | 0.881 | 0.136 | 9.30E-11 |
| 18 | Sleep duration | rs365663 | A | G | 0.546 | 0.878 | 0.137 | 1.00E-10 |
| 19 | Sleep duration | rs1517572 | C | A | 0.581 | 0.879 | 0.138 | 1.50E-10 |
| 20 | Sleep duration | rs7915425 | T | C | 0.175 | 1.144 | 0.179 | 2.00E-10 |
| 21 | Sleep duration | rs330088 | C | T | 0.547 | 0.868 | 0.137 | 2.70E-10 |
| 22 | Sleep duration | rs8038326 | A | G | 0.727 | 0.955 | 0.152 | 2.80E-10 |
| 23 | Sleep duration | rs460692 | C | T | 0.137 | 1.263 | 0.2 | 3.60E-10 |
| 24 | Sleep duration | rs9382445 | T | C | 0.623 | 0.872 | 0.14 | 4.80E-10 |
| 25 | Sleep duration | rs4767550 | G | A | 0.414 | 0.858 | 0.139 | 6.30E-10 |
| 26 | Sleep duration | rs11885663 | T | C | 0.248 | 0.973 | 0.157 | 8.60E-10 |
| 27 | Sleep duration | rs1991556 | G | A | 0.774 | 0.994 | 0.163 | 1.00E-09 |
| 28 | Sleep duration | rs1057703 | G | T | 0.147 | 1.164 | 0.192 | 1.10E-09 |
| 29 | Sleep duration | rs4128364 | C | T | 0.339 | 0.876 | 0.143 | 1.40E-09 |
| 30 | Sleep duration | rs10483350 | G | A | 0.195 | 1.042 | 0.172 | 1.50E-09 |
| 31 | Sleep duration | rs61796569 | T | C | 0.27 | 0.927 | 0.154 | 1.50E-09 |
| 32 | Sleep duration | rs7115226 | A | C | 0.074 | 1.594 | 0.261 | 1.70E-09 |
| 33 | Sleep duration | rs269054 | A | T | 0.422 | 0.819 | 0.138 | 2.10E-09 |
| 34 | Sleep duration | rs112230981 | A | G | 0.95 | 1.892 | 0.314 | 2.20E-09 |
| 35 | Sleep duration | rs11602180 | C | T | 0.837 | 1.095 | 0.184 | 2.30E-09 |
| 36 | Sleep duration | rs2192528 | A | G | 0.48 | 0.802 | 0.136 | 2.70E-09 |
| 37 | Sleep duration | rs12246842 | A | G | 0.46 | 0.804 | 0.136 | 3.90E-09 |
| 38 | Sleep duration | rs205024 | T | C | 0.384 | 0.83 | 0.14 | 3.90E-09 |
| 39 | Sleep duration | rs12567114 | A | G | 0.276 | 0.89 | 0.152 | 4.30E-09 |
| 40 | Sleep duration | rs7616632 | T | G | 0.522 | 0.792 | 0.136 | 4.30E-09 |
| 41 | Sleep duration | rs6575005 | T | C | 0.758 | 0.934 | 0.159 | 4.40E-09 |
| 42 | Sleep duration | rs1776776 | T | C | 0.874 | 1.198 | 0.205 | 4.90E-09 |
| 43 | Sleep duration | rs11621908 | C | T | 0.917 | 1.446 | 0.25 | 5.60E-09 |
| 44 | Sleep duration | rs10421649 | A | T | 0.557 | 0.798 | 0.138 | 6.90E-09 |
| 45 | Sleep duration | rs2072727 | T | C | 0.436 | 0.795 | 0.137 | 7.90E-09 |
| 46 | Sleep duration | rs113113059 | T | C | 0.78 | 0.968 | 0.164 | 8.40E-09 |
| 47 | Sleep duration | rs151014368 | A | G | 0.206 | 0.966 | 0.169 | 9.10E-09 |
| 48 | Sleep duration | rs374153 | C | T | 0.158 | 1.057 | 0.186 | 9.10E-09 |
| 49 | Sleep duration | rs62120041 | T | C | 0.934 | 1.567 | 0.274 | 9.60E-09 |
| 50 | Sleep duration | rs7503199 | C | T | 0.734 | 0.885 | 0.154 | 1.00E-08 |
| 51 | Sleep duration | rs17732997 | C | G | 0.569 | 0.776 | 0.137 | 1.20E-08 |
| 52 | Sleep duration | rs1939455 | G | T | 0.879 | 1.226 | 0.214 | 1.20E-08 |
| 53 | Sleep duration | rs7951019 | G | T | 0.032 | 2.213 | 0.391 | 1.20E-08 |
| 54 | Sleep duration | rs17427571 | A | G | 0.684 | 0.83 | 0.146 | 1.30E-08 |
| 55 | Sleep duration | rs61985058 | T | C | 0.143 | 1.116 | 0.194 | 1.30E-08 |
| 56 | Sleep duration | rs7806045 | T | C | 0.755 | 0.887 | 0.158 | 1.40E-08 |
| 57 | Sleep duration | rs35531607 | C | T | 0.474 | 0.77 | 0.136 | 1.50E-08 |
| 58 | Sleep duration | rs7644809 | T | C | 0.422 | 0.784 | 0.138 | 1.60E-08 |
| 59 | Sleep duration | rs9345234 | C | A | 0.578 | 0.781 | 0.138 | 1.80E-08 |
| 60 | Sleep duration | rs12791153 | T | A | 0.081 | 1.413 | 0.253 | 1.90E-08 |
| 61 | Sleep duration | rs1263056 | A | G | 0.519 | 0.768 | 0.137 | 2.00E-08 |
| 62 | Sleep duration | rs55658675 | C | T | 0.645 | 0.788 | 0.142 | 2.00E-08 |
| 63 | Sleep duration | rs11567976 | T | C | 0.571 | 0.768 | 0.137 | 2.10E-08 |
| 64 | Sleep duration | rs180769 | T | C | 0.425 | 0.763 | 0.138 | 2.30E-08 |
| 65 | Sleep duration | rs1553132 | G | A | 0.258 | 0.87 | 0.155 | 2.50E-08 |
| 66 | Sleep duration | rs9903973 | C | T | 0.467 | 0.766 | 0.136 | 2.60E-08 |
| 67 | Sleep duration | rs11614986 | A | G | 0.821 | 0.983 | 0.177 | 2.70E-08 |
| 68 | Sleep duration | rs2231265 | G | A | 0.772 | 0.897 | 0.162 | 2.70E-08 |
| 69 | Sleep duration | rs174560 | C | T | 0.314 | 0.815 | 0.146 | 2.80E-08 |
| 70 | Sleep duration | rs10173260 | C | T | 0.606 | 0.77 | 0.139 | 2.90E-08 |
| 71 | Sleep duration | rs72804080 | G | A | 0.15 | 1.068 | 0.192 | 2.90E-08 |
| 72 | Sleep duration | rs12611523 | A | G | 0.545 | 0.758 | 0.137 | 3.10E-08 |
| 73 | Sleep duration | rs11643715 | G | C | 0.291 | 0.834 | 0.15 | 3.20E-08 |
| 74 | Sleep duration | rs4538155 | T | C | 0.647 | 0.779 | 0.142 | 3.60E-08 |
| 75 | Sleep duration | rs34354917 | C | A | 0.71 | 0.825 | 0.15 | 3.90E-08 |
| 76 | Sleep duration | rs80193650 | G | A | 0.162 | 1.01 | 0.184 | 4.10E-08 |
| 77 | Sleep duration | rs10761674 | C | T | 0.477 | 0.74 | 0.136 | 4.20E-08 |
| 78 | Sleep duration | rs11190970 | G | A | 0.799 | 0.923 | 0.169 | 4.60E-08 |

**Supplementary Table 4.** The summary information for instrumental variables of short sleep duration.

| **Sort** | **Phenotype** | **SNP** | **effect_allele** | **other_allele** | **beta** | **se** | **eaf** | **pval** |
| --- | --- | --- | --- | --- | --- | --- | --- | --- |
| 1 | Short sleep duration | rs11763750 | G | A | 0.433 | 0.074 | 0.814 | 5.10E-09 |
| 2 | Short sleep duration | rs1229762 | T | C | 0.434 | 0.061 | 0.665 | 1.10E-12 |
| 3 | Short sleep duration | rs12518468 | C | T | 0.353 | 0.061 | 0.328 | 8.50E-09 |
| 4 | Short sleep duration | rs12567114 | G | A | 0.379 | 0.065 | 0.725 | 4.10E-09 |
| 5 | Short sleep duration | rs12661667 | T | C | 0.361 | 0.065 | 0.263 | 2.80E-08 |
| 6 | Short sleep duration | rs12963463 | C | T | 0.427 | 0.064 | 0.299 | 1.90E-11 |
| 7 | Short sleep duration | rs13107325 | T | C | 0.796 | 0.11 | 0.075 | 2.50E-13 |
| 8 | Short sleep duration | rs1380703 | G | A | 0.406 | 0.06 | 0.384 | 1.60E-11 |
| 9 | Short sleep duration | rs142180737 | C | T | 1.856 | 0.319 | 0.009 | 4.40E-09 |
| 10 | Short sleep duration | rs1607227 | G | T | 0.382 | 0.063 | 0.705 | 1.50E-09 |
| 11 | Short sleep duration | rs17005118 | A | G | 0.389 | 0.065 | 0.265 | 2.50E-09 |
| 12 | Short sleep duration | rs17388803 | C | A | 0.59 | 0.095 | 0.106 | 6.50E-10 |
| 13 | Short sleep duration | rs2014830 | C | T | 0.347 | 0.063 | 0.698 | 2.70E-08 |
| 14 | Short sleep duration | rs205024 | C | T | 0.331 | 0.059 | 0.617 | 2.70E-08 |
| 15 | Short sleep duration | rs2186122 | T | A | 0.34 | 0.058 | 0.562 | 4.80E-09 |
| 16 | Short sleep duration | rs2820313 | G | A | 0.36 | 0.061 | 0.341 | 2.30E-09 |
| 17 | Short sleep duration | rs2863957 | C | A | 0.611 | 0.07 | 0.782 | 2.60E-18 |
| 18 | Short sleep duration | rs3776864 | A | C | 0.343 | 0.061 | 0.667 | 1.70E-08 |
| 19 | Short sleep duration | rs4585442 | G | A | 0.381 | 0.062 | 0.311 | 8.10E-10 |
| 20 | Short sleep duration | rs5757675 | G | T | 0.387 | 0.066 | 0.26 | 2.70E-09 |
| 21 | Short sleep duration | rs59779556 | T | G | 0.329 | 0.058 | 0.554 | 2.00E-08 |
| 22 | Short sleep duration | rs60882754 | A | T | 0.678 | 0.12 | 0.939 | 1.80E-08 |
| 23 | Short sleep duration | rs7524118 | C | T | 0.346 | 0.063 | 0.708 | 4.90E-08 |
| 24 | Short sleep duration | rs75539574 | A | C | 0.669 | 0.104 | 0.915 | 8.40E-11 |
| 25 | Short sleep duration | rs7939345 | T | G | 0.39 | 0.071 | 0.208 | 4.00E-08 |
| 26 | Short sleep duration | rs9321171 | C | T | 0.321 | 0.058 | 0.54 | 4.20E-08 |
| 27 | Short sleep duration | rs9367621 | T | A | 0.327 | 0.058 | 0.431 | 1.60E-08 |

**Supplementary Table 5.** The summary information for instrumental variables of long sleep duration.

| **Sort** | **Phenotype** | **SNP** | **effect_allele** | **other_allele** | **beta** | **se** | **eaf** | **pval** |
| --- | --- | --- | --- | --- | --- | --- | --- | --- |
| 1 | Long sleep duration | rs6737318 | G | A | 0.383 | 0.053 | 0.222 | 3.40E-13 |
| 2 | Long sleep duration | rs75458655 | T | C | 1.004 | 0.145 | 0.023 | 5.40E-12 |
| 3 | Long sleep duration | rs17688916 | T | A | 0.373 | 0.055 | 0.204 | 1.10E-11 |
| 4 | Long sleep duration | rs17817288 | A | G | 0.251 | 0.044 | 0.482 | 8.90E-09 |
| 5 | Long sleep duration | rs549961083 | T | C | 3.549 | 0.618 | 0.001 | 9.60E-09 |
| 6 | Long sleep duration | rs3751046 | G | A | 0.346 | 0.062 | 0.147 | 2.00E-08 |
| 7 | Long sleep duration | rs12145723 | C | T | 0.294 | 0.055 | 0.197 | 2.10E-08 |
| 8 | Long sleep duration | rs10899257 | A | G | 0.338 | 0.062 | 0.144 | 4.60E-08 |

**Supplementary Table 6.** The summary information for instrumental variables of insomnia.

| **Sort** | **Phenotype** | **SNP** | **effect_allele** | **other_allele** | **beta** | **se** | **eaf** | **pval** |
| --- | --- | --- | --- | --- | --- | --- | --- | --- |
| 1 | Insomnia | rs113851554 | T | G | 0.206 | 0.014 | 0.051 | 1.56E-51 |
| 2 | Insomnia | rs9527083 | G | A | 0.076 | 0.006 | 0.33 | 1.61E-32 |
| 3 | Insomnia | rs10947428 | C | T | 0.068 | 0.007 | 0.214 | 9.06E-21 |
| 4 | Insomnia | rs6119267 | G | C | 0.06 | 0.006 | 0.311 | 2.32E-20 |
| 5 | Insomnia | rs62158170 | A | G | 0.066 | 0.007 | 0.786 | 1.20E-19 |
| 6 | Insomnia | rs9394502 | C | T | 0.054 | 0.006 | 0.666 | 7.76E-18 |
| 7 | Insomnia | rs55972276 | A | C | 0.073 | 0.009 | 0.137 | 4.19E-17 |
| 8 | Insomnia | rs2431108 | C | T | 0.053 | 0.006 | 0.328 | 7.83E-17 |
| 9 | Insomnia | rs7566062 | T | C | 0.059 | 0.007 | 0.225 | 1.37E-16 |
| 10 | Insomnia | rs8180817 | G | C | 0.049 | 0.006 | 0.57 | 1.83E-16 |
| 11 | Insomnia | rs118166957 | T | C | 0.068 | 0.008 | 0.159 | 1.95E-16 |
| 12 | Insomnia | rs13135092 | G | A | 0.089 | 0.011 | 0.083 | 2.53E-16 |
| 13 | Insomnia | rs35322724 | A | C | 0.049 | 0.006 | 0.577 | 3.75E-16 |
| 14 | Insomnia | rs4702 | G | A | 0.048 | 0.006 | 0.444 | 6.78E-16 |
| 15 | Insomnia | rs16903122 | T | C | 0.055 | 0.007 | 0.249 | 9.04E-16 |
| 16 | Insomnia | rs77641763 | T | C | 0.071 | 0.009 | 0.122 | 6.53E-15 |
| 17 | Insomnia | rs1927902 | T | C | 0.053 | 0.007 | 0.254 | 1.15E-14 |
| 18 | Insomnia | rs62264767 | A | C | 0.065 | 0.008 | 0.853 | 1.63E-14 |
| 19 | Insomnia | rs1620977 | A | G | 0.052 | 0.007 | 0.27 | 2.27E-14 |
| 20 | Insomnia | rs1015438 | A | G | 0.058 | 0.008 | 0.188 | 2.51E-14 |
| 21 | Insomnia | rs17643634 | C | T | 0.06 | 0.008 | 0.835 | 1.34E-13 |
| 22 | Insomnia | rs28582096 | G | A | 0.054 | 0.007 | 0.795 | 1.74E-13 |
| 23 | Insomnia | rs694786 | C | T | 0.044 | 0.006 | 0.54 | 1.97E-13 |
| 24 | Insomnia | rs2815757 | T | C | 0.055 | 0.008 | 0.809 | 2.24E-13 |
| 25 | Insomnia | rs78206187 | G | A | 0.094 | 0.013 | 0.056 | 2.96E-13 |
| 26 | Insomnia | rs12912299 | C | T | 0.043 | 0.006 | 0.511 | 4.42E-13 |
| 27 | Insomnia | rs2903385 | A | G | 0.043 | 0.006 | 0.484 | 4.53E-13 |
| 28 | Insomnia | rs7214267 | G | A | 0.044 | 0.006 | 0.419 | 5.09E-13 |
| 29 | Insomnia | rs314281 | C | T | 0.043 | 0.006 | 0.547 | 6.03E-13 |
| 30 | Insomnia | rs11605348 | G | A | 0.045 | 0.006 | 0.651 | 7.01E-13 |
| 31 | Insomnia | rs4981170 | G | A | 0.054 | 0.008 | 0.806 | 7.33E-13 |
| 32 | Insomnia | rs670501 | T | C | 0.053 | 0.007 | 0.213 | 7.40E-13 |
| 33 | Insomnia | rs11126082 | G | C | 0.043 | 0.006 | 0.56 | 8.26E-13 |
| 34 | Insomnia | rs9931543 | T | C | 0.048 | 0.007 | 0.736 | 1.11E-12 |
| 35 | Insomnia | rs524859 | G | A | 0.044 | 0.006 | 0.64 | 1.48E-12 |
| 36 | Insomnia | rs72657797 | C | T | 0.056 | 0.008 | 0.824 | 1.52E-12 |
| 37 | Insomnia | rs60565673 | G | T | 0.043 | 0.006 | 0.379 | 1.59E-12 |
| 38 | Insomnia | rs10761240 | G | A | 0.043 | 0.006 | 0.604 | 2.12E-12 |
| 39 | Insomnia | rs66674044 | T | A | 0.06 | 0.009 | 0.143 | 2.18E-12 |
| 40 | Insomnia | rs12666306 | A | G | 0.042 | 0.006 | 0.502 | 2.24E-12 |
| 41 | Insomnia | rs10800992 | T | C | 0.042 | 0.006 | 0.443 | 3.84E-12 |
| 42 | Insomnia | rs1031654 | C | A | 0.051 | 0.007 | 0.2 | 3.88E-12 |
| 43 | Insomnia | rs10947690 | G | A | 0.047 | 0.007 | 0.259 | 4.04E-12 |
| 44 | Insomnia | rs9889282 | C | A | 0.042 | 0.006 | 0.387 | 4.70E-12 |
| 45 | Insomnia | rs56133505 | A | G | 0.041 | 0.006 | 0.537 | 5.59E-12 |
| 46 | Insomnia | rs7044885 | G | C | 0.041 | 0.006 | 0.558 | 5.67E-12 |
| 47 | Insomnia | rs3774751 | G | T | 0.041 | 0.006 | 0.538 | 7.32E-12 |
| 48 | Insomnia | rs61921611 | C | T | 0.044 | 0.006 | 0.308 | 7.84E-12 |
| 49 | Insomnia | rs715338 | A | G | 0.041 | 0.006 | 0.578 | 7.85E-12 |
| 50 | Insomnia | rs830716 | C | G | 0.045 | 0.007 | 0.713 | 8.68E-12 |
| 51 | Insomnia | rs13010288 | G | T | 0.06 | 0.009 | 0.867 | 9.26E-12 |
| 52 | Insomnia | rs12983032 | G | A | 0.043 | 0.006 | 0.657 | 1.07E-11 |
| 53 | Insomnia | rs8180457 | C | T | 0.056 | 0.008 | 0.843 | 1.12E-11 |
| 54 | Insomnia | rs951807 | C | T | 0.041 | 0.006 | 0.59 | 1.19E-11 |
| 55 | Insomnia | rs908668 | T | C | 0.05 | 0.007 | 0.208 | 1.41E-11 |
| 56 | Insomnia | rs79693059 | G | C | 0.073 | 0.011 | 0.084 | 1.61E-11 |
| 57 | Insomnia | rs10865954 | T | C | 0.042 | 0.006 | 0.334 | 1.92E-11 |
| 58 | Insomnia | rs9373590 | A | T | 0.04 | 0.006 | 0.508 | 2.18E-11 |
| 59 | Insomnia | rs12991815 | C | G | 0.04 | 0.006 | 0.424 | 3.02E-11 |
| 60 | Insomnia | rs61765555 | C | T | 0.045 | 0.007 | 0.745 | 4.00E-11 |
| 61 | Insomnia | rs12310246 | A | G | 0.045 | 0.007 | 0.249 | 4.74E-11 |
| 62 | Insomnia | rs55772859 | A | C | 0.042 | 0.006 | 0.311 | 4.82E-11 |
| 63 | Insomnia | rs6808140 | T | C | 0.039 | 0.006 | 0.505 | 5.35E-11 |
| 64 | Insomnia | rs67501351 | C | G | 0.045 | 0.007 | 0.745 | 5.36E-11 |
| 65 | Insomnia | rs2286729 | A | G | 0.07 | 0.011 | 0.086 | 5.37E-11 |
| 66 | Insomnia | rs11803128 | G | A | 0.041 | 0.006 | 0.346 | 6.85E-11 |
| 67 | Insomnia | rs28611339 | T | G | 0.058 | 0.009 | 0.128 | 8.46E-11 |
| 68 | Insomnia | rs10502966 | G | A | 0.039 | 0.006 | 0.418 | 8.54E-11 |
| 69 | Insomnia | rs35110063 | A | G | 0.039 | 0.006 | 0.427 | 8.82E-11 |
| 70 | Insomnia | rs2792990 | C | G | 0.054 | 0.008 | 0.855 | 1.15E-10 |
| 71 | Insomnia | rs6888135 | A | C | 0.038 | 0.006 | 0.497 | 1.21E-10 |
| 72 | Insomnia | rs6562066 | T | C | 0.039 | 0.006 | 0.369 | 1.38E-10 |
| 73 | Insomnia | rs6967168 | G | T | 0.044 | 0.007 | 0.246 | 1.39E-10 |
| 74 | Insomnia | rs4643373 | T | C | 0.041 | 0.007 | 0.701 | 1.58E-10 |
| 75 | Insomnia | rs12187443 | T | C | 0.04 | 0.006 | 0.668 | 1.64E-10 |
| 76 | Insomnia | rs1861412 | A | G | 0.038 | 0.006 | 0.434 | 1.67E-10 |
| 77 | Insomnia | rs2389631 | C | A | 0.04 | 0.006 | 0.333 | 2.03E-10 |
| 78 | Insomnia | rs116466468 | T | C | 0.044 | 0.007 | 0.759 | 2.11E-10 |
| 79 | Insomnia | rs1064939 | A | T | 0.13 | 0.02 | 0.978 | 2.16E-10 |
| 80 | Insomnia | rs4238755 | C | A | 0.043 | 0.007 | 0.736 | 2.30E-10 |
| 81 | Insomnia | rs17223714 | A | G | 0.046 | 0.007 | 0.789 | 2.44E-10 |
| 82 | Insomnia | rs1038093 | T | C | 0.039 | 0.006 | 0.628 | 2.47E-10 |
| 83 | Insomnia | rs224029 | C | T | 0.039 | 0.006 | 0.601 | 2.51E-10 |
| 84 | Insomnia | rs3902952 | T | C | 0.048 | 0.008 | 0.188 | 2.55E-10 |
| 85 | Insomnia | rs56097173 | T | C | 0.04 | 0.006 | 0.681 | 2.69E-10 |
| 86 | Insomnia | rs2089358 | C | T | 0.041 | 0.007 | 0.296 | 2.75E-10 |
| 87 | Insomnia | rs8076183 | C | T | 0.038 | 0.006 | 0.552 | 2.75E-10 |
| 88 | Insomnia | rs671985 | G | A | 0.038 | 0.006 | 0.548 | 2.79E-10 |
| 89 | Insomnia | rs12251016 | T | A | 0.039 | 0.006 | 0.344 | 3.89E-10 |
| 90 | Insomnia | rs4592425 | T | G | 0.04 | 0.006 | 0.697 | 4.31E-10 |
| 91 | Insomnia | rs12790660 | C | T | 0.04 | 0.006 | 0.316 | 4.49E-10 |
| 92 | Insomnia | rs152555 | G | A | 0.052 | 0.008 | 0.146 | 4.83E-10 |
| 93 | Insomnia | rs823247 | C | T | 0.037 | 0.006 | 0.521 | 5.25E-10 |
| 94 | Insomnia | rs73671843 | G | A | 0.056 | 0.009 | 0.874 | 5.49E-10 |
| 95 | Insomnia | rs17005118 | A | G | 0.042 | 0.007 | 0.264 | 6.13E-10 |
| 96 | Insomnia | rs1064213 | G | A | 0.037 | 0.006 | 0.521 | 6.41E-10 |
| 97 | Insomnia | rs4767645 | G | T | 0.037 | 0.006 | 0.539 | 6.47E-10 |
| 98 | Insomnia | rs6019663 | T | C | 0.04 | 0.007 | 0.293 | 6.47E-10 |
| 99 | Insomnia | rs984306 | C | T | 0.043 | 0.007 | 0.245 | 7.94E-10 |
| 100 | Insomnia | rs4502882 | C | T | 0.039 | 0.006 | 0.342 | 7.96E-10 |
| 101 | Insomnia | rs1530938 | A | G | 0.036 | 0.006 | 0.442 | 8.82E-10 |
| 102 | Insomnia | rs8181889 | G | A | 0.038 | 0.006 | 0.601 | 8.90E-10 |
| 103 | Insomnia | rs1264419 | C | G | 0.036 | 0.006 | 0.513 | 8.91E-10 |
| 104 | Insomnia | rs3184470 | G | A | 0.038 | 0.006 | 0.649 | 9.73E-10 |
| 105 | Insomnia | rs1147852 | A | G | 0.039 | 0.006 | 0.31 | 9.94E-10 |
| 106 | Insomnia | rs72899452 | T | C | 0.074 | 0.012 | 0.065 | 1.00E-09 |
| 107 | Insomnia | rs6756610 | C | G | 0.037 | 0.006 | 0.629 | 1.14E-09 |
| 108 | Insomnia | rs62068188 | T | C | 0.049 | 0.008 | 0.834 | 1.18E-09 |
| 109 | Insomnia | rs6702604 | G | A | 0.037 | 0.006 | 0.416 | 1.30E-09 |
| 110 | Insomnia | rs2388840 | G | A | 0.037 | 0.006 | 0.424 | 1.37E-09 |
| 111 | Insomnia | rs9540729 | A | T | 0.036 | 0.006 | 0.479 | 1.40E-09 |
| 112 | Insomnia | rs324017 | A | C | 0.039 | 0.007 | 0.294 | 1.61E-09 |
| 113 | Insomnia | rs12520974 | C | T | 0.036 | 0.006 | 0.515 | 1.69E-09 |
| 114 | Insomnia | rs4790076 | T | C | 0.048 | 0.008 | 0.174 | 1.76E-09 |
| 115 | Insomnia | rs62429521 | A | C | 0.051 | 0.008 | 0.146 | 1.78E-09 |
| 116 | Insomnia | rs11090039 | A | G | 0.039 | 0.007 | 0.287 | 1.82E-09 |
| 117 | Insomnia | rs6734957 | G | T | 0.042 | 0.007 | 0.761 | 1.82E-09 |
| 118 | Insomnia | rs6465151 | T | C | 0.056 | 0.009 | 0.113 | 1.90E-09 |
| 119 | Insomnia | rs2221119 | C | G | 0.036 | 0.006 | 0.443 | 2.00E-09 |
| 120 | Insomnia | rs12605642 | T | G | 0.035 | 0.006 | 0.486 | 2.13E-09 |
| 121 | Insomnia | rs11149313 | A | G | 0.04 | 0.007 | 0.73 | 2.38E-09 |
| 122 | Insomnia | rs2598293 | T | C | 0.035 | 0.006 | 0.476 | 2.48E-09 |
| 123 | Insomnia | rs7615602 | G | C | 0.04 | 0.007 | 0.729 | 2.59E-09 |
| 124 | Insomnia | rs4709655 | C | T | 0.054 | 0.009 | 0.881 | 3.09E-09 |
| 125 | Insomnia | rs11679943 | A | G | 0.037 | 0.006 | 0.347 | 3.16E-09 |
| 126 | Insomnia | rs214934 | T | A | 0.038 | 0.006 | 0.688 | 3.16E-09 |
| 127 | Insomnia | rs34214423 | A | C | 0.045 | 0.008 | 0.809 | 3.18E-09 |
| 128 | Insomnia | rs71575448 | A | G | 0.051 | 0.009 | 0.86 | 3.38E-09 |
| 129 | Insomnia | rs492858 | C | T | 0.066 | 0.011 | 0.924 | 3.46E-09 |
| 130 | Insomnia | rs72773790 | T | C | 0.037 | 0.006 | 0.673 | 3.71E-09 |
| 131 | Insomnia | rs7040224 | A | G | 0.037 | 0.006 | 0.316 | 4.24E-09 |
| 132 | Insomnia | rs34967082 | A | G | 0.035 | 0.006 | 0.414 | 4.34E-09 |
| 133 | Insomnia | rs45453598 | A | T | 0.047 | 0.008 | 0.169 | 4.42E-09 |
| 134 | Insomnia | rs35539975 | A | G | 0.042 | 0.007 | 0.779 | 4.49E-09 |
| 135 | Insomnia | rs62194948 | C | G | 0.039 | 0.007 | 0.275 | 4.64E-09 |
| 136 | Insomnia | rs6589988 | G | A | 0.038 | 0.006 | 0.324 | 4.70E-09 |
| 137 | Insomnia | rs37445 | G | A | 0.036 | 0.006 | 0.61 | 4.88E-09 |
| 138 | Insomnia | rs10758593 | G | A | 0.036 | 0.006 | 0.601 | 4.90E-09 |
| 139 | Insomnia | rs12454003 | G | C | 0.035 | 0.006 | 0.518 | 4.94E-09 |
| 140 | Insomnia | rs4664299 | C | T | 0.041 | 0.007 | 0.765 | 4.95E-09 |
| 141 | Insomnia | rs11588755 | G | A | 0.035 | 0.006 | 0.478 | 5.14E-09 |
| 142 | Insomnia | rs7402939 | C | T | 0.036 | 0.006 | 0.624 | 5.19E-09 |
| 143 | Insomnia | rs7625896 | A | G | 0.036 | 0.006 | 0.655 | 5.28E-09 |
| 144 | Insomnia | rs871994 | A | C | 0.035 | 0.006 | 0.435 | 5.50E-09 |
| 145 | Insomnia | rs9316619 | T | C | 0.046 | 0.008 | 0.825 | 5.50E-09 |
| 146 | Insomnia | rs1289939 | C | T | 0.041 | 0.007 | 0.768 | 6.00E-09 |
| 147 | Insomnia | rs1536053 | C | T | 0.038 | 0.006 | 0.684 | 6.04E-09 |
| 148 | Insomnia | rs4788203 | G | A | 0.035 | 0.006 | 0.567 | 6.32E-09 |
| 149 | Insomnia | rs7475916 | G | C | 0.037 | 0.006 | 0.647 | 6.70E-09 |
| 150 | Insomnia | rs701394 | G | A | 0.036 | 0.006 | 0.362 | 6.83E-09 |
| 151 | Insomnia | rs2838787 | G | A | 0.036 | 0.006 | 0.608 | 7.65E-09 |
| 152 | Insomnia | rs874168 | T | C | 0.034 | 0.006 | 0.525 | 7.95E-09 |
| 153 | Insomnia | rs10944696 | G | A | 0.038 | 0.007 | 0.702 | 7.99E-09 |
| 154 | Insomnia | rs10898940 | A | C | 0.034 | 0.006 | 0.517 | 8.09E-09 |
| 155 | Insomnia | rs12030482 | A | T | 0.041 | 0.007 | 0.22 | 8.16E-09 |
| 156 | Insomnia | rs4090240 | C | T | 0.039 | 0.007 | 0.723 | 8.46E-09 |
| 157 | Insomnia | rs1167132 | T | C | 0.035 | 0.006 | 0.392 | 8.73E-09 |
| 158 | Insomnia | rs17367725 | C | T | 0.036 | 0.006 | 0.649 | 9.29E-09 |
| 159 | Insomnia | rs176644 | T | G | 0.035 | 0.006 | 0.404 | 9.49E-09 |
| 160 | Insomnia | rs728017 | G | A | 0.035 | 0.006 | 0.614 | 9.51E-09 |
| 161 | Insomnia | rs2030672 | C | G | 0.034 | 0.006 | 0.559 | 1.10E-08 |
| 162 | Insomnia | rs6457796 | C | T | 0.039 | 0.007 | 0.269 | 1.12E-08 |
| 163 | Insomnia | rs7992992 | A | G | 0.051 | 0.009 | 0.129 | 1.15E-08 |
| 164 | Insomnia | rs11119409 | C | T | 0.035 | 0.006 | 0.413 | 1.19E-08 |
| 165 | Insomnia | rs11650304 | C | G | 0.067 | 0.012 | 0.931 | 1.23E-08 |
| 166 | Insomnia | rs4858708 | T | A | 0.034 | 0.006 | 0.47 | 1.23E-08 |
| 167 | Insomnia | rs5877 | T | C | 0.036 | 0.006 | 0.669 | 1.23E-08 |
| 168 | Insomnia | rs72820274 | A | G | 0.034 | 0.006 | 0.417 | 1.28E-08 |
| 169 | Insomnia | rs11756035 | C | G | 0.051 | 0.009 | 0.128 | 1.29E-08 |
| 170 | Insomnia | rs1731951 | T | A | 0.035 | 0.006 | 0.557 | 1.36E-08 |
| 171 | Insomnia | rs62301574 | G | C | 0.042 | 0.007 | 0.2 | 1.37E-08 |
| 172 | Insomnia | rs73163783 | C | T | 0.038 | 0.007 | 0.277 | 1.39E-08 |
| 173 | Insomnia | rs7571486 | G | A | 0.039 | 0.007 | 0.749 | 1.40E-08 |
| 174 | Insomnia | rs138678612 | G | A | 0.117 | 0.02 | 0.022 | 1.41E-08 |
| 175 | Insomnia | rs10825503 | T | G | 0.033 | 0.006 | 0.487 | 1.43E-08 |
| 176 | Insomnia | rs6606731 | A | T | 0.043 | 0.008 | 0.192 | 1.51E-08 |
| 177 | Insomnia | rs521484 | G | A | 0.04 | 0.007 | 0.233 | 1.53E-08 |
| 178 | Insomnia | rs4588900 | A | G | 0.033 | 0.006 | 0.516 | 1.57E-08 |
| 179 | Insomnia | rs75452188 | A | G | 0.052 | 0.009 | 0.878 | 1.58E-08 |
| 180 | Insomnia | rs17083297 | C | A | 0.044 | 0.008 | 0.823 | 1.60E-08 |
| 181 | Insomnia | rs2216427 | C | G | 0.035 | 0.006 | 0.653 | 1.60E-08 |
| 182 | Insomnia | rs10928256 | T | C | 0.034 | 0.006 | 0.419 | 1.61E-08 |
| 183 | Insomnia | rs910187 | G | A | 0.035 | 0.006 | 0.627 | 1.63E-08 |
| 184 | Insomnia | rs34490907 | C | G | 0.054 | 0.009 | 0.888 | 1.76E-08 |
| 185 | Insomnia | rs10756571 | T | C | 0.036 | 0.006 | 0.685 | 1.80E-08 |
| 186 | Insomnia | rs7168238 | C | G | 0.064 | 0.011 | 0.074 | 1.80E-08 |
| 187 | Insomnia | rs10955647 | T | G | 0.033 | 0.006 | 0.532 | 1.84E-08 |
| 188 | Insomnia | rs1519102 | G | C | 0.037 | 0.006 | 0.311 | 1.90E-08 |
| 189 | Insomnia | rs12924275 | T | C | 0.038 | 0.007 | 0.268 | 1.93E-08 |
| 190 | Insomnia | rs13138995 | A | G | 0.034 | 0.006 | 0.39 | 1.97E-08 |
| 191 | Insomnia | rs16990210 | C | T | 0.046 | 0.008 | 0.152 | 1.97E-08 |
| 192 | Insomnia | rs1937447 | G | C | 0.039 | 0.007 | 0.241 | 2.08E-08 |
| 193 | Insomnia | rs6978112 | T | C | 0.034 | 0.006 | 0.411 | 2.11E-08 |
| 194 | Insomnia | rs2364921 | C | T | 0.034 | 0.006 | 0.531 | 2.13E-08 |
| 195 | Insomnia | rs769449 | G | A | 0.046 | 0.008 | 0.846 | 2.13E-08 |
| 196 | Insomnia | rs1567084 | A | G | 0.033 | 0.006 | 0.498 | 2.14E-08 |
| 197 | Insomnia | rs17025198 | A | G | 0.041 | 0.007 | 0.204 | 2.19E-08 |
| 198 | Insomnia | rs6601080 | A | G | 0.035 | 0.006 | 0.676 | 2.21E-08 |
| 199 | Insomnia | rs638746 | A | G | 0.033 | 0.006 | 0.579 | 2.26E-08 |
| 200 | Insomnia | rs1580173 | A | G | 0.033 | 0.006 | 0.561 | 2.28E-08 |
| 201 | Insomnia | rs62213452 | T | G | 0.037 | 0.007 | 0.279 | 2.39E-08 |
| 202 | Insomnia | rs742760 | A | T | 0.043 | 0.008 | 0.816 | 2.48E-08 |
| 203 | Insomnia | rs11001276 | T | A | 0.038 | 0.007 | 0.26 | 2.52E-08 |
| 204 | Insomnia | rs76145129 | G | T | 0.05 | 0.009 | 0.876 | 2.73E-08 |
| 205 | Insomnia | rs17520265 | G | A | 0.091 | 0.016 | 0.966 | 2.87E-08 |
| 206 | Insomnia | rs647905 | T | C | 0.033 | 0.006 | 0.541 | 2.87E-08 |
| 207 | Insomnia | rs11722569 | T | C | 0.034 | 0.006 | 0.659 | 2.91E-08 |
| 208 | Insomnia | rs12917449 | C | A | 0.042 | 0.008 | 0.194 | 2.97E-08 |
| 209 | Insomnia | rs6597649 | T | C | 0.033 | 0.006 | 0.399 | 3.05E-08 |
| 210 | Insomnia | rs9563886 | C | T | 0.034 | 0.006 | 0.394 | 3.08E-08 |
| 211 | Insomnia | rs623025 | C | T | 0.038 | 0.007 | 0.745 | 3.16E-08 |
| 212 | Insomnia | rs28552587 | A | G | 0.033 | 0.006 | 0.564 | 3.30E-08 |
| 213 | Insomnia | rs238869 | C | T | 0.034 | 0.006 | 0.377 | 3.36E-08 |
| 214 | Insomnia | rs2737240 | A | G | 0.036 | 0.007 | 0.708 | 3.37E-08 |
| 215 | Insomnia | rs138014720 | A | T | 0.07 | 0.013 | 0.941 | 3.46E-08 |
| 216 | Insomnia | rs1553754 | G | T | 0.034 | 0.006 | 0.438 | 3.51E-08 |
| 217 | Insomnia | rs117630493 | G | C | 0.101 | 0.018 | 0.027 | 3.61E-08 |
| 218 | Insomnia | rs73079014 | C | T | 0.049 | 0.009 | 0.874 | 3.65E-08 |
| 219 | Insomnia | rs2867690 | T | C | 0.042 | 0.008 | 0.182 | 3.70E-08 |
| 220 | Insomnia | rs4699157 | C | T | 0.081 | 0.015 | 0.042 | 3.98E-08 |
| 221 | Insomnia | rs62383308 | G | A | 0.06 | 0.011 | 0.92 | 3.98E-08 |
| 222 | Insomnia | rs10947987 | C | T | 0.033 | 0.006 | 0.557 | 4.08E-08 |
| 223 | Insomnia | rs699844 | A | G | 0.06 | 0.011 | 0.92 | 4.11E-08 |
| 224 | Insomnia | rs75932578 | C | T | 0.04 | 0.007 | 0.784 | 4.15E-08 |
| 225 | Insomnia | rs6973090 | G | A | 0.038 | 0.007 | 0.75 | 4.31E-08 |
| 226 | Insomnia | rs9964420 | A | C | 0.035 | 0.007 | 0.301 | 4.54E-08 |
| 227 | Insomnia | rs6510033 | G | A | 0.037 | 0.007 | 0.275 | 4.66E-08 |
| 228 | Insomnia | rs4260410 | T | C | 0.034 | 0.006 | 0.332 | 4.87E-08 |

**Supplementary Table 7.** The summary information for instrumental variables of daytime sleepiness.

| **Sort** | **Phenotype** | **SNP** | **beta** | **se** | **effect_allele** | **other_allele** | **pval** |
| --- | --- | --- | --- | --- | --- | --- | --- |
| 1 | Daytime sleepiness | rs2787120 | 0.00778398 | 0.00137756 | A | G | 2.00E-08 |
| 2 | Daytime sleepiness | rs12140153 | 0.01658 | 0.00179639 | T | G | 2.80E-20 |
| 3 | Daytime sleepiness | rs17131124 | -0.0111768 | 0.00183679 | C | G | 1.70E-09 |
| 4 | Daytime sleepiness | rs57746981 | 0.00680998 | 0.00107584 | C | T | 2.20E-10 |
| 5 | Daytime sleepiness | rs825127 | 0.00591296 | 0.00103275 | T | G | 9.50E-09 |
| 6 | Daytime sleepiness | rs4665972 | 0.0066245 | 0.00105853 | T | C | 3.90E-10 |
| 7 | Daytime sleepiness | rs7598712 | 0.00579387 | 0.00104191 | G | T | 2.20E-08 |
| 8 | Daytime sleepiness | rs6741951 | 0.00681617 | 0.00114107 | A | G | 2.70E-09 |
| 9 | Daytime sleepiness | rs11123962 | -0.00804433 | 0.00103439 | T | G | 7.50E-15 |
| 10 | Daytime sleepiness | rs9712275 | -0.00588405 | 0.0010319 | C | T | 1.30E-08 |
| 11 | Daytime sleepiness | rs7607363 | -0.0060244 | 0.00103841 | A | G | 8.00E-09 |
| 12 | Daytime sleepiness | rs13010456 | 0.00774142 | 0.00105125 | A | G | 2.10E-13 |
| 13 | Daytime sleepiness | rs13097760 | -0.00598755 | 0.00107776 | A | C | 3.20E-08 |
| 14 | Daytime sleepiness | rs34478464 | -0.00858848 | 0.00130506 | C | T | 5.80E-11 |
| 15 | Daytime sleepiness | rs960986 | 0.00716034 | 0.00106799 | C | T | 1.50E-11 |
| 16 | Daytime sleepiness | rs843372 | 0.00816368 | 0.00122597 | C | T | 2.20E-11 |
| 17 | Daytime sleepiness | rs11942333 | -0.00605348 | 0.00110327 | G | A | 3.80E-08 |
| 18 | Daytime sleepiness | rs13135092 | -0.0103303 | 0.0018774 | A | G | 3.10E-08 |
| 19 | Daytime sleepiness | rs6897863 | 0.00645716 | 0.00104732 | A | C | 7.60E-10 |
| 20 | Daytime sleepiness | rs12153518 | 0.00670287 | 0.00103166 | A | C | 6.80E-11 |
| 21 | Daytime sleepiness | rs6923811 | 0.00677376 | 0.00110514 | T | C | 9.10E-10 |
| 22 | Daytime sleepiness | rs55960940 | 0.00762668 | 0.00135164 | T | C | 2.00E-08 |
| 23 | Daytime sleepiness | rs3122170 | 0.00950345 | 0.00122688 | C | A | 5.60E-15 |
| 24 | Daytime sleepiness | rs62519825 | -0.0094794 | 0.00162829 | T | C | 3.80E-09 |
| 25 | Daytime sleepiness | rs285793 | 0.00676457 | 0.00103539 | G | A | 7.90E-11 |
| 26 | Daytime sleepiness | rs7837226 | -0.00574201 | 0.00103269 | A | G | 2.00E-08 |
| 27 | Daytime sleepiness | rs55818482 | -0.00973113 | 0.00125942 | T | C | 1.40E-14 |
| 28 | Daytime sleepiness | rs1566362 | 0.00631993 | 0.0010687 | T | C | 3.80E-09 |
| 29 | Daytime sleepiness | rs7476897 | 0.00743007 | 0.00110192 | G | A | 2.70E-11 |
| 30 | Daytime sleepiness | rs4765939 | -0.00627707 | 0.00104601 | G | C | 2.00E-09 |
| 31 | Daytime sleepiness | rs1846644 | -0.0113546 | 0.00104777 | T | C | 2.50E-27 |
| 32 | Daytime sleepiness | rs8015449 | 0.00619154 | 0.00103462 | A | G | 1.90E-09 |
| 33 | Daytime sleepiness | rs17356118 | -0.00765559 | 0.00122087 | A | G | 2.60E-10 |
| 34 | Daytime sleepiness | rs886114 | 0.00604278 | 0.00107429 | C | T | 1.90E-08 |
| 35 | Daytime sleepiness | rs11078398 | 0.00766031 | 0.00124122 | G | A | 7.10E-10 |
| 36 | Daytime sleepiness | rs62066119 | 0.00831345 | 0.00121332 | C | T | 7.30E-12 |
| 37 | Daytime sleepiness | rs2048522 | 0.00581823 | 0.00105065 | A | T | 3.50E-08 |

**Supplementary Table 8.** The summary information for instrumental variables of SLE.

| **Sort** | **Phenotype** | **SNP** | **other_allele** | **effect_allele** | **pval** | **beta** | **se** |
| --- | --- | --- | --- | --- | --- | --- | --- |
| 1 | SLE | rs4661543 | G | T | 9.40E-11 | -0.274436846 | 0.042375456 |
| 2 | SLE | rs6679677 | C | A | 4.55E-13 | 0.336472237 | 0.04648538 |
| 3 | SLE | rs6671847 | G | A | 6.64E-12 | 0.198850859 | 0.028965081 |
| 4 | SLE | rs10912578 | G | A | 1.65E-15 | 0.246860078 | 0.030991799 |
| 5 | SLE | rs4916215 | T | C | 5.07E-11 | -0.223143551 | 0.033969323 |
| 6 | SLE | rs17849501 | C | T | 1.81E-59 | 0.810930216 | 0.049864233 |
| 7 | SLE | rs12094036 | T | C | 1.37E-08 | -0.328504067 | 0.057859483 |
| 8 | SLE | rs34703115 | T | C | 4.08E-09 | -0.616186139 | 0.104777606 |
| 9 | SLE | rs268124 | T | C | 8.60E-09 | -0.186329578 | 0.032370264 |
| 10 | SLE | rs13019891 | G | T | 1.65E-83 | -0.562118918 | 0.029033597 |
| 11 | SLE | rs2459611 | T | C | 7.62E-09 | -0.261364764 | 0.045245001 |
| 12 | SLE | rs4274624 | T | C | 9.73E-66 | 0.559615788 | 0.032679116 |
| 13 | SLE | rs10048743 | T | G | 2.04E-08 | 0.231111721 | 0.041205628 |
| 14 | SLE | rs10200680 | C | T | 4.96E-09 | -0.248461359 | 0.042483496 |
| 15 | SLE | rs2573219 | A | C | 1.13E-42 | 0.587786665 | 0.042929166 |
| 16 | SLE | rs9852014 | A | G | 2.26E-36 | 0.620576488 | 0.049272685 |
| 17 | SLE | rs1464446 | G | T | 2.79E-16 | -0.328504067 | 0.040149728 |
| 18 | SLE | rs13136219 | C | T | 3.50E-10 | -0.174353387 | 0.027786961 |
| 19 | SLE | rs4388254 | C | T | 3.71E-10 | 0.378436436 | 0.060397671 |
| 20 | SLE | rs1078324 | C | A | 7.11E-20 | -0.713349888 | 0.078166469 |
| 21 | SLE | rs6889239 | T | C | 2.19E-18 | 0.277631737 | 0.031739962 |
| 22 | SLE | rs2431697 | T | C | 2.60E-14 | -0.223143551 | 0.029296429 |
| 23 | SLE | rs150180633 | C | T | 2.66E-41 | 0.928219303 | 0.068957329 |
| 24 | SLE | rs1270942 | A | G | 1.45E-101 | 0.928219303 | 0.043382175 |
| 25 | SLE | rs143810596 | T | G | 4.41E-08 | -0.616186139 | 0.112573796 |
| 26 | SLE | rs9274357 | C | T | 1.28E-38 | 0.457424847 | 0.035196132 |
| 27 | SLE | rs7768653 | T | C | 3.11E-12 | 0.207014169 | 0.029689071 |
| 28 | SLE | rs58721818 | C | T | 3.38E-18 | 0.657520003 | 0.075594067 |
| 29 | SLE | rs35000415 | C | T | 1.86E-45 | 0.587786665 | 0.041538955 |
| 30 | SLE | rs2736332 | G | C | 4.83E-18 | 0.277631737 | 0.03206938 |
| 31 | SLE | rs7823055 | T | G | 1.64E-34 | 0.350656872 | 0.028620839 |
| 32 | SLE | rs7097397 | G | A | 8.60E-11 | -0.186329578 | 0.028711836 |
| 33 | SLE | rs7899626 | C | T | 4.19E-08 | 0.182321557 | 0.033253186 |
| 34 | SLE | rs58688157 | A | G | 2.97E-11 | -0.223143551 | 0.033564737 |
| 35 | SLE | rs353608 | G | A | 2.93E-11 | -0.186329578 | 0.028019771 |
| 36 | SLE | rs73050535 | C | T | 9.11E-09 | -0.713349888 | 0.124134163 |
| 37 | SLE | rs597808 | A | G | 3.51E-08 | -0.162518929 | 0.029473642 |
| 38 | SLE | rs1143679 | G | A | 5.03E-48 | 0.58221562 | 0.039986634 |
| 39 | SLE | rs13332649 | A | G | 5.43E-17 | -0.314710745 | 0.037568252 |
| 40 | SLE | rs143123127 | G | A | 2.23E-08 | 0.470003629 | 0.084034201 |
| 41 | SLE | rs35251378 | G | A | 3.61E-13 | -0.235722334 | 0.03242656 |
| 42 | SLE | rs73068668 | G | A | 4.40E-08 | -0.314710745 | 0.057490346 |
| 43 | SLE | rs3747093 | G | A | 2.88E-14 | 0.262364264 | 0.034505488 |

**Supplementary Table 9.** SNPs as instrumental variables in the study of the effect of sleep traits on SLE risk.

| **Exposure** | **SNP** | **Effect**  **allele** | **Other**  **allele** | **Exposure** | | | **Outcome** | | | **Proxy SNP** | **Target**  **effect allele** | **Target**  **other allele** |
| --- | --- | --- | --- | --- | --- | --- | --- | --- | --- | --- | --- | --- |
|  |  |  |  | **Beta** | **SE** | P value | **Beta** | **SE** | P value |  |  |  |
| Chronotype | rs10058356 | T | C | -0.017 | 0.003 | 6.80E-09 | -0.020 | 0.031 | 0.521 |  |  |  |
|  | rs10175975 | T | C | 0.023 | 0.004 | 1.80E-10 | 0.049 | 0.033 | 0.141 |  |  |  |
|  | rs10237162 | T | C | 0.018 | 0.003 | 3.50E-09 | -0.010 | 0.050 | 0.843 |  |  |  |
|  | rs10402849 | T | C | 0.019 | 0.003 | 8.90E-09 | -0.010 | 0.039 | 0.796 | rs4807315 | T | C |
|  | rs10520176 | T | C | 0.023 | 0.003 | 1.40E-17 | -0.051 | 0.032 | 0.104 |  |  |  |
|  | rs1061032 | T | G | 0.039 | 0.005 | 2.00E-16 | 0.068 | 0.042 | 0.109 |  |  |  |
|  | rs1064213 | A | G | 0.019 | 0.003 | 1.30E-12 | 0.062 | 0.029 | 0.030 |  |  |  |
|  | rs10742179 | A | G | 0.018 | 0.003 | 1.50E-09 | 0.010 | 0.059 | 0.867 | rs4347344 | A | G |
|  | rs10832648 | A | C | -0.020 | 0.003 | 3.20E-09 | -0.010 | 0.034 | 0.767 |  |  |  |
|  | rs10916892 | T | C | -0.020 | 0.003 | 6.10E-13 | -0.020 | 0.030 | 0.502 |  |  |  |
|  | rs10951325 | T | C | 0.021 | 0.003 | 7.90E-14 | -0.039 | 0.027 | 0.151 |  |  |  |
|  | rs10988239 | T | C | -0.019 | 0.003 | 1.60E-11 | 0.010 | 0.042 | 0.813 |  |  |  |
|  | rs11032362 | A | G | 0.040 | 0.005 | 4.80E-17 | 0.010 | 0.067 | 0.882 |  |  |  |
|  | rs11152350 | A | C | -0.016 | 0.003 | 1.30E-09 | 0.039 | 0.027 | 0.150 |  |  |  |
|  | rs113851554 | T | G | -0.036 | 0.006 | 1.00E-09 | -0.051 | 0.069 | 0.455 |  |  |  |
|  | rs1144566 | T | C | 0.113 | 0.008 | 5.80E-47 | -0.073 | 0.098 | 0.459 |  |  |  |
|  | rs11545787 | A | G | -0.024 | 0.003 | 4.90E-14 | 0.010 | 0.053 | 0.850 |  |  |  |
|  | rs11588913 | A | G | -0.015 | 0.003 | 1.50E-08 | 0.020 | 0.035 | 0.569 |  |  |  |
|  | rs11670534 | T | C | -0.021 | 0.004 | 8.50E-09 | 0.030 | 0.047 | 0.528 |  |  |  |
|  | rs12040629 | A | G | 0.039 | 0.004 | 6.70E-27 | -0.041 | 0.043 | 0.345 |  |  |  |
|  | rs12140153 | T | G | -0.034 | 0.005 | 1.60E-13 | -0.010 | 0.071 | 0.887 |  |  |  |
|  | rs12298405 | T | C | -0.016 | 0.003 | 2.70E-08 | 0.077 | 0.028 | 0.007 |  |  |  |
|  | rs12636669 | T | C | 0.034 | 0.005 | 5.80E-12 | -0.062 | 0.062 | 0.316 |  |  |  |
|  | rs12808544 | A | C | -0.021 | 0.003 | 3.00E-11 | 0.010 | 0.040 | 0.802 |  |  |  |
|  | rs12969848 | T | C | 0.022 | 0.003 | 1.30E-15 | -0.030 | 0.031 | 0.335 |  |  |  |
|  | rs13065394 | T | G | -0.018 | 0.003 | 3.00E-09 | 0.030 | 0.030 | 0.324 |  |  |  |
|  | rs13377754 | T | C | 0.030 | 0.003 | 6.90E-27 | -0.077 | 0.029 | 0.008 |  |  |  |
|  | rs139911 | T | C | -0.023 | 0.003 | 2.30E-17 | 0.020 | 0.022 | 0.360 |  |  |  |
|  | rs1421085 | T | C | -0.028 | 0.003 | 1.50E-23 | -0.020 | 0.027 | 0.467 |  |  |  |
|  | rs1468945 | A | G | -0.023 | 0.003 | 4.20E-12 | 0.073 | 0.038 | 0.053 |  |  |  |
|  | rs1599374 | A | G | 0.015 | 0.003 | 1.50E-08 | 0.010 | 0.033 | 0.762 | rs4414866 | G | A |
|  | rs16939162 | A | G | 0.025 | 0.004 | 2.20E-12 | -0.039 | 0.036 | 0.269 |  |  |  |
|  | rs17302081 | T | C | 0.016 | 0.003 | 6.90E-09 | -0.020 | 0.025 | 0.415 |  |  |  |
|  | rs17448682 | T | C | 0.022 | 0.003 | 2.20E-12 | 0.049 | 0.034 | 0.155 |  |  |  |
|  | rs17575798 | A | G | -0.023 | 0.003 | 4.20E-11 | -0.041 | 0.041 | 0.322 |  |  |  |
|  | rs17604349 | A | G | -0.026 | 0.004 | 1.70E-13 | -0.030 | 0.034 | 0.364 |  |  |  |
|  | rs1886205 | A | C | 0.019 | 0.003 | 4.90E-10 | 0.062 | 0.034 | 0.069 |  |  |  |
|  | rs2011528 | T | C | -0.021 | 0.004 | 5.30E-09 | -0.010 | 0.035 | 0.774 |  |  |  |
|  | rs2072727 | T | C | 0.016 | 0.003 | 8.50E-10 | 0.058 | 0.029 | 0.043 |  |  |  |
|  | rs2166559 | T | C | -0.022 | 0.004 | 9.10E-09 | -0.058 | 0.040 | 0.146 |  |  |  |
|  | rs2362775 | T | C | -0.018 | 0.003 | 2.80E-11 | -0.010 | 0.056 | 0.860 | rs13093492 | C | T |
|  | rs28458909 | T | C | -0.043 | 0.004 | 2.60E-26 | -0.051 | 0.052 | 0.328 |  |  |  |
|  | rs2881955 | T | C | 0.016 | 0.003 | 4.30E-08 | -0.020 | 0.037 | 0.588 |  |  |  |
|  | rs2916148 | A | G | 0.016 | 0.003 | 3.50E-09 | -0.030 | 0.028 | 0.272 |  |  |  |
|  | rs2944831 | A | G | 0.017 | 0.003 | 1.10E-08 | 0.010 | 0.028 | 0.718 |  |  |  |
|  | rs2979139 | A | G | -0.017 | 0.003 | 7.40E-10 | -0.094 | 0.030 | 0.002 |  |  |  |
|  | rs308521 | T | C | 0.020 | 0.003 | 3.70E-13 | -0.010 | 0.025 | 0.695 |  |  |  |
|  | rs3100052 | A | G | 0.017 | 0.003 | 6.90E-10 | -0.010 | 0.026 | 0.698 |  |  |  |
|  | rs3808964 | T | G | 0.016 | 0.003 | 2.70E-08 | -0.049 | 0.028 | 0.077 |  |  |  |
|  | rs4241964 | T | G | -0.021 | 0.003 | 1.10E-14 | -0.020 | 0.023 | 0.380 |  |  |  |
|  | rs4269995 | T | C | -0.021 | 0.003 | 5.90E-12 | -0.041 | 0.031 | 0.190 |  |  |  |
|  | rs4419127 | A | G | 0.022 | 0.003 | 1.30E-14 | 0.030 | 0.030 | 0.314 |  |  |  |
|  | rs4690085 | A | G | -0.015 | 0.003 | 3.90E-08 | 0.073 | 0.028 | 0.009 |  |  |  |
|  | rs486416 | A | G | -0.015 | 0.003 | 2.30E-08 | -0.432 | 0.031 | 0.000 |  |  |  |
|  | rs4936290 | A | C | -0.018 | 0.003 | 4.60E-10 | -0.020 | 0.029 | 0.501 |  |  |  |
|  | rs55846845 | A | G | -0.016 | 0.003 | 2.40E-09 | 0.020 | 0.037 | 0.585 |  |  |  |
|  | rs60616179 | A | G | 0.038 | 0.006 | 1.10E-10 | 0.041 | 0.063 | 0.520 |  |  |  |
|  | rs6131805 | T | G | 0.016 | 0.003 | 1.50E-08 | -0.010 | 0.033 | 0.762 |  |  |  |
|  | rs6131942 | A | G | -0.018 | 0.003 | 9.40E-11 | -0.020 | 0.037 | 0.589 |  |  |  |
|  | rs61773390 | T | G | 0.034 | 0.003 | 1.20E-23 | 0.020 | 0.033 | 0.544 |  |  |  |
|  | rs62082402 | T | G | 0.026 | 0.003 | 1.60E-14 | 0.039 | 0.034 | 0.253 |  |  |  |
|  | rs62182135 | A | C | -0.018 | 0.003 | 3.70E-10 | 0.030 | 0.032 | 0.348 |  |  |  |
|  | rs6477309 | T | C | 0.017 | 0.003 | 8.00E-10 | 0.041 | 0.028 | 0.141 |  |  |  |
|  | rs6727752 | A | G | 0.016 | 0.003 | 1.40E-08 | -0.073 | 0.034 | 0.034 |  |  |  |
|  | rs6794796 | A | G | 0.017 | 0.003 | 5.30E-09 | -0.073 | 0.035 | 0.036 |  |  |  |
|  | rs6846730 | T | C | -0.022 | 0.003 | 5.60E-12 | -0.010 | 0.075 | 0.894 | rs1058358 | T | C |
|  | rs6993892 | T | C | -0.021 | 0.003 | 8.40E-14 | -0.020 | 0.028 | 0.474 |  |  |  |
|  | rs7111582 | A | G | -0.028 | 0.004 | 5.20E-10 | 0.030 | 0.055 | 0.578 |  |  |  |
|  | rs7203707 | A | C | -0.016 | 0.003 | 7.60E-10 | 0.062 | 0.028 | 0.030 |  |  |  |
|  | rs72720396 | A | G | -0.027 | 0.003 | 8.40E-18 | -0.068 | 0.036 | 0.057 |  |  |  |
|  | rs72829706 | A | G | 0.041 | 0.007 | 5.10E-09 | 0.030 | 0.073 | 0.676 |  |  |  |
|  | rs7304278 | A | G | -0.020 | 0.003 | 2.20E-11 | 0.030 | 0.029 | 0.301 |  |  |  |
|  | rs73050286 | T | C | 0.018 | 0.003 | 2.60E-08 | -0.049 | 0.031 | 0.118 |  |  |  |
|  | rs7429614 | T | G | 0.016 | 0.003 | 9.20E-10 | 0.010 | 0.021 | 0.632 | rs4683992 | T | G |
|  | rs74357745 | A | G | 0.027 | 0.004 | 8.70E-11 | 0.041 | 0.041 | 0.322 |  |  |  |
|  | rs75120545 | T | C | 0.061 | 0.008 | 1.10E-13 | -0.105 | 0.087 | 0.224 |  |  |  |
|  | rs77960 | A | G | 0.019 | 0.003 | 4.20E-11 | -0.020 | 0.031 | 0.510 |  |  |  |
|  | rs7845620 | A | C | -0.022 | 0.004 | 5.20E-10 | 0.010 | 0.042 | 0.809 |  |  |  |
|  | rs7959983 | T | C | -0.020 | 0.003 | 9.50E-13 | -0.010 | 0.021 | 0.639 | rs7139279 | C | T |
|  | rs80097534 | T | G | -0.028 | 0.005 | 9.30E-10 | -0.030 | 0.047 | 0.513 |  |  |  |
|  | rs80271258 | T | C | -0.055 | 0.005 | 2.60E-30 | -0.062 | 0.056 | 0.269 |  |  |  |
|  | rs9348050 | T | C | 0.017 | 0.003 | 7.50E-10 | 0.010 | 0.026 | 0.697 |  |  |  |
|  | rs9381812 | A | G | -0.023 | 0.003 | 4.60E-15 | -0.030 | 0.027 | 0.276 |  |  |  |
|  | rs9416744 | A | C | 0.019 | 0.003 | 1.30E-09 | 0.010 | 0.031 | 0.751 |  |  |  |
|  | rs9436119 | A | G | 0.023 | 0.003 | 4.00E-18 | -0.010 | 0.021 | 0.634 |  |  |  |
|  | rs9479402 | T | C | -0.107 | 0.013 | 3.20E-16 | -0.030 | 0.113 | 0.794 |  |  |  |
|  | rs9573980 | A | G | 0.073 | 0.007 | 5.70E-22 | -0.039 | 0.083 | 0.637 |  |  |  |
|  | rs9597241 | A | C | 0.021 | 0.003 | 7.50E-10 | 0.051 | 0.036 | 0.150 |  |  |  |
|  | rs962961 | T | C | -0.016 | 0.003 | 2.00E-08 | 0.039 | 0.030 | 0.198 |  |  |  |
|  | rs9636202 | A | G | -0.018 | 0.003 | 7.50E-10 | -0.030 | 0.030 | 0.308 |  |  |  |
|  | rs975025 | T | C | -0.032 | 0.005 | 1.30E-10 | 0.010 | 0.086 | 0.908 |  |  |  |
|  | rs9964420 | A | C | -0.028 | 0.003 | 8.60E-22 | -0.010 | 0.028 | 0.721 |  |  |  |
| Sleep duration | rs10173260 | C | T | 0.606 | 0.770 | 2.90E-08 | -0.010 | 0.022 | 0.649 |  |  |  |
|  | rs10483350 | G | A | 0.195 | 1.042 | 1.50E-09 | -0.051 | 0.039 | 0.191 |  |  |  |
|  | rs1057703 | G | T | 0.147 | 1.164 | 1.10E-09 | -0.030 | 0.046 | 0.506 | rs116522584 | G | T |
|  | rs10761674 | C | T | 0.477 | 0.740 | 4.20E-08 | 0.039 | 0.026 | 0.134 |  |  |  |
|  | rs10973207 | T | G | 0.158 | 1.226 | 6.00E-11 | 0.039 | 0.041 | 0.338 |  |  |  |
|  | rs11190970 | G | A | 0.799 | 0.923 | 4.60E-08 | 0.030 | 0.043 | 0.480 |  |  |  |
|  | rs112230981 | A | G | 0.950 | 1.892 | 2.20E-09 | -0.030 | 0.096 | 0.759 |  |  |  |
|  | rs113113059 | T | C | 0.780 | 0.968 | 8.40E-09 | 0.073 | 0.038 | 0.057 |  |  |  |
|  | rs11567976 | T | C | 0.571 | 0.768 | 2.10E-08 | -0.020 | 0.026 | 0.447 |  |  |  |
|  | rs11602180 | C | T | 0.837 | 1.095 | 2.30E-09 | 0.010 | 0.028 | 0.719 |  |  |  |
|  | rs11621908 | C | T | 0.917 | 1.446 | 5.60E-09 | -0.020 | 0.051 | 0.696 |  |  |  |
|  | rs11885663 | T | C | 0.248 | 0.973 | 8.60E-10 | 0.030 | 0.028 | 0.298 |  |  |  |
|  | rs12246842 | A | G | 0.460 | 0.804 | 3.90E-09 | 0.020 | 0.029 | 0.488 |  |  |  |
|  | rs12567114 | A | G | 0.276 | 0.890 | 4.30E-09 | -0.041 | 0.030 | 0.176 |  |  |  |
|  | rs12607679 | T | C | 0.738 | 1.208 | 8.30E-15 | -0.020 | 0.028 | 0.473 | rs4374254 | C | T |
|  | rs13088093 | G | T | 0.336 | 0.976 | 7.00E-12 | 0.010 | 0.046 | 0.829 |  |  |  |
|  | rs13109404 | T | G | 0.928 | 1.872 | 1.40E-12 | 0.041 | 0.057 | 0.472 |  |  |  |
|  | rs151014368 | A | G | 0.206 | 0.966 | 9.10E-09 | 0.010 | 0.040 | 0.802 |  |  |  |
|  | rs1517572 | C | A | 0.581 | 0.879 | 1.50E-10 | 0.041 | 0.031 | 0.194 |  |  |  |
|  | rs1553132 | G | A | 0.258 | 0.870 | 2.50E-08 | 0.068 | 0.030 | 0.025 |  |  |  |
|  | rs17427571 | A | G | 0.684 | 0.830 | 1.30E-08 | -0.020 | 0.022 | 0.358 |  |  |  |
|  | rs174560 | C | T | 0.314 | 0.815 | 2.80E-08 | -0.083 | 0.032 | 0.010 |  |  |  |
|  | rs1776776 | T | C | 0.874 | 1.198 | 4.90E-09 | 0.062 | 0.047 | 0.190 |  |  |  |
|  | rs1939455 | G | T | 0.879 | 1.226 | 1.20E-08 | -0.039 | 0.041 | 0.336 |  |  |  |
|  | rs1991556 | G | A | 0.774 | 0.994 | 1.00E-09 | 0.105 | 0.033 | 0.001 |  |  |  |
|  | rs205024 | T | C | 0.384 | 0.830 | 3.90E-09 | -0.010 | 0.032 | 0.754 |  |  |  |
|  | rs2072727 | T | C | 0.436 | 0.795 | 7.90E-09 | 0.058 | 0.029 | 0.043 |  |  |  |
|  | rs2192528 | A | G | 0.480 | 0.802 | 2.70E-09 | -0.049 | 0.027 | 0.075 | rs10027920 | A | G |
|  | rs2231265 | G | A | 0.772 | 0.897 | 2.70E-08 | 0.041 | 0.038 | 0.288 |  |  |  |
|  | rs3095508 | C | A | 0.594 | 0.921 | 3.10E-11 | 0.020 | 0.027 | 0.456 |  |  |  |
|  | rs34354917 | C | A | 0.710 | 0.825 | 3.90E-08 | -0.010 | 0.024 | 0.674 |  |  |  |
|  | rs34556183 | A | G | 0.720 | 1.015 | 2.30E-11 | -0.182 | 0.033 | 0.000 |  |  |  |
|  | rs34731055 | T | C | 0.181 | 1.168 | 3.70E-11 | 0.020 | 0.040 | 0.622 |  |  |  |
|  | rs35531607 | C | T | 0.474 | 0.770 | 1.50E-08 | 0.010 | 0.047 | 0.832 |  |  |  |
|  | rs365663 | A | G | 0.546 | 0.878 | 1.00E-10 | -0.010 | 0.041 | 0.810 |  |  |  |
|  | rs374153 | C | T | 0.158 | 1.057 | 9.10E-09 | -0.010 | 0.074 | 0.893 | rs7570522 | C | T |
|  | rs4128364 | C | T | 0.339 | 0.876 | 1.40E-09 | -0.010 | 0.022 | 0.650 |  |  |  |
|  | rs4592416 | G | A | 0.464 | 0.881 | 9.30E-11 | 0.010 | 0.033 | 0.762 | rs4755213 | G | A |
|  | rs4767550 | G | A | 0.414 | 0.858 | 6.30E-10 | 0.039 | 0.026 | 0.135 |  |  |  |
|  | rs55658675 | C | T | 0.645 | 0.788 | 2.00E-08 | 0.073 | 0.034 | 0.031 |  |  |  |
|  | rs61796569 | T | C | 0.270 | 0.927 | 1.50E-09 | 0.030 | 0.036 | 0.410 |  |  |  |
|  | rs61985058 | T | C | 0.143 | 1.116 | 1.30E-08 | -0.062 | 0.044 | 0.163 |  |  |  |
|  | rs62120041 | T | C | 0.934 | 1.567 | 9.60E-09 | 0.030 | 0.059 | 0.603 |  |  |  |
|  | rs62362521 | G | A | 0.334 | 1.017 | 2.20E-12 | 0.073 | 0.033 | 0.026 |  |  |  |
|  | rs6575005 | T | C | 0.758 | 0.934 | 4.40E-09 | 0.073 | 0.034 | 0.034 |  |  |  |
|  | rs72804080 | G | A | 0.150 | 1.068 | 2.90E-08 | -0.051 | 0.043 | 0.229 |  |  |  |
|  | rs73219758 | G | A | 0.708 | 0.984 | 5.60E-11 | -0.010 | 0.056 | 0.859 | rs34805259 | A | G |
|  | rs75539574 | C | A | 0.086 | 2.175 | 6.90E-19 | 0.039 | 0.050 | 0.433 |  |  |  |
|  | rs7556815 | A | G | 0.219 | 2.443 | 1.30E-49 | -0.020 | 0.041 | 0.618 | rs62158211 | A | G |
|  | rs7644809 | T | C | 0.422 | 0.784 | 1.60E-08 | -0.030 | 0.030 | 0.307 |  |  |  |
|  | rs7806045 | T | C | 0.755 | 0.887 | 1.40E-08 | -0.020 | 0.034 | 0.563 |  |  |  |
|  | rs7915425 | T | C | 0.175 | 1.144 | 2.00E-10 | 0.049 | 0.033 | 0.144 |  |  |  |
|  | rs8038326 | A | G | 0.727 | 0.955 | 2.80E-10 | 0.094 | 0.033 | 0.004 |  |  |  |
|  | rs9345234 | C | A | 0.578 | 0.781 | 1.80E-08 | -0.039 | 0.031 | 0.201 |  |  |  |
|  | rs9382445 | T | C | 0.623 | 0.872 | 4.80E-10 | -0.020 | 0.026 | 0.441 |  |  |  |
| Short sleep duration | rs11763750 | G | A | 0.433 | 0.074 | 5.10E-09 | -0.030 | 0.034 | 0.386 |  |  |  |
|  | rs1229762 | T | C | 0.434 | 0.061 | 1.10E-12 | 0.010 | 0.029 | 0.726 |  |  |  |
|  | rs12518468 | C | T | 0.353 | 0.061 | 8.50E-09 | -0.010 | 0.041 | 0.806 |  |  |  |
|  | rs12567114 | G | A | 0.379 | 0.065 | 4.10E-09 | 0.041 | 0.030 | 0.176 |  |  |  |
|  | rs12661667 | T | C | 0.361 | 0.065 | 2.80E-08 | 0.077 | 0.033 | 0.019 |  |  |  |
|  | rs12963463 | C | T | 0.427 | 0.064 | 1.90E-11 | 0.039 | 0.033 | 0.241 |  |  |  |
|  | rs13107325 | T | C | 0.796 | 0.110 | 2.50E-13 | -0.105 | 0.050 | 0.034 |  |  |  |
|  | rs1380703 | G | A | 0.406 | 0.060 | 1.60E-11 | -0.073 | 0.035 | 0.035 |  |  |  |
|  | rs17005118 | A | G | 0.389 | 0.065 | 2.50E-09 | 0.010 | 0.022 | 0.644 |  |  |  |
|  | rs17388803 | C | A | 0.590 | 0.095 | 6.50E-10 | -0.105 | 0.042 | 0.012 |  |  |  |
|  | rs2014830 | C | T | 0.347 | 0.063 | 2.70E-08 | 0.083 | 0.032 | 0.010 |  |  |  |
|  | rs205024 | C | T | 0.331 | 0.059 | 2.70E-08 | 0.010 | 0.032 | 0.754 |  |  |  |
|  | rs2820313 | G | A | 0.360 | 0.061 | 2.30E-09 | -0.030 | 0.032 | 0.345 |  |  |  |
|  | rs2863957 | C | A | 0.611 | 0.070 | 2.60E-18 | 0.020 | 0.032 | 0.531 | rs4618068 | A | C |
|  | rs3776864 | A | C | 0.343 | 0.061 | 1.70E-08 | 0.094 | 0.035 | 0.007 |  |  |  |
|  | rs4585442 | G | A | 0.381 | 0.062 | 8.10E-10 | 0.020 | 0.037 | 0.588 |  |  |  |
|  | rs5757675 | G | T | 0.387 | 0.066 | 2.70E-09 | 0.039 | 0.025 | 0.120 |  |  |  |
|  | rs59779556 | T | G | 0.329 | 0.058 | 2.00E-08 | -0.020 | 0.025 | 0.421 |  |  |  |
|  | rs7524118 | C | T | 0.346 | 0.063 | 4.90E-08 | -0.010 | 0.030 | 0.740 |  |  |  |
|  | rs7939345 | T | G | 0.390 | 0.071 | 4.00E-08 | -0.041 | 0.034 | 0.230 |  |  |  |
|  | rs9321171 | C | T | 0.321 | 0.058 | 4.20E-08 | 0.030 | 0.032 | 0.335 |  |  |  |
| Long sleep duration | rs10899257 | A | G | 0.338 | 0.062 | 4.60E-08 | -0.062 | 0.043 | 0.148 |  |  |  |
|  | rs12145723 | C | T | 0.294 | 0.055 | 2.10E-08 | -0.041 | 0.038 | 0.277 |  |  |  |
|  | rs17817288 | A | G | 0.251 | 0.044 | 8.90E-09 | -0.010 | 0.046 | 0.828 |  |  |  |
|  | rs3751046 | G | A | 0.346 | 0.062 | 2.00E-08 | -0.020 | 0.036 | 0.570 | rs73606751 | G | A |
|  | rs6737318 | G | A | 0.383 | 0.053 | 3.40E-13 | -0.020 | 0.039 | 0.607 | rs56093896 | G | A |
|  | rs75458655 | T | C | 1.004 | 0.145 | 5.40E-12 | -0.117 | 0.096 | 0.225 |  |  |  |
| Insomnia | rs1015438 | A | G | 0.058 | 0.008 | 2.51E-14 | -0.020 | 0.032 | 0.528 |  |  |  |
|  | rs1031654 | C | A | 0.051 | 0.007 | 3.88E-12 | -0.105 | 0.039 | 0.007 |  |  |  |
|  | rs1038093 | T | C | 0.039 | 0.006 | 2.47E-10 | -0.010 | 0.054 | 0.855 | rs2127018 | C | T |
|  | rs10756571 | T | C | 0.036 | 0.006 | 1.80E-08 | -0.010 | 0.053 | 0.852 |  |  |  |
|  | rs10758593 | G | A | 0.036 | 0.006 | 4.90E-09 | -0.010 | 0.039 | 0.801 |  |  |  |
|  | rs10761240 | G | A | 0.043 | 0.006 | 2.12E-12 | 0.083 | 0.031 | 0.007 |  |  |  |
|  | rs10800992 | T | C | 0.042 | 0.006 | 3.84E-12 | 0.010 | 0.028 | 0.723 |  |  |  |
|  | rs10898940 | A | C | 0.034 | 0.006 | 8.09E-09 | 0.010 | 0.033 | 0.757 |  |  |  |
|  | rs10944696 | G | A | 0.038 | 0.007 | 7.99E-09 | 0.020 | 0.035 | 0.560 |  |  |  |
|  | rs10947428 | C | T | 0.068 | 0.007 | 9.06E-21 | 0.039 | 0.039 | 0.310 |  |  |  |
|  | rs10947690 | G | A | 0.047 | 0.007 | 4.04E-12 | -0.030 | 0.039 | 0.438 |  |  |  |
|  | rs10955647 | T | G | 0.033 | 0.006 | 1.84E-08 | -0.030 | 0.025 | 0.229 |  |  |  |
|  | rs11090039 | A | G | 0.039 | 0.007 | 1.82E-09 | 0.010 | 0.046 | 0.830 |  |  |  |
|  | rs11119409 | C | T | 0.035 | 0.006 | 1.19E-08 | -0.030 | 0.027 | 0.251 |  |  |  |
|  | rs11149313 | A | G | 0.040 | 0.007 | 2.38E-09 | -0.010 | 0.025 | 0.695 |  |  |  |
|  | rs113851554 | T | G | 0.206 | 0.014 | 1.56E-51 | -0.051 | 0.069 | 0.455 |  |  |  |
|  | rs1147852 | A | G | 0.039 | 0.006 | 9.94E-10 | -0.062 | 0.031 | 0.045 |  |  |  |
|  | rs11588755 | G | A | 0.035 | 0.006 | 5.14E-09 | -0.010 | 0.026 | 0.694 |  |  |  |
|  | rs11605348 | G | A | 0.045 | 0.006 | 7.01E-13 | 0.062 | 0.033 | 0.057 |  |  |  |
|  | rs116466468 | T | C | 0.044 | 0.007 | 2.11E-10 | 0.041 | 0.038 | 0.278 |  |  |  |
|  | rs1167132 | T | C | 0.035 | 0.006 | 8.73E-09 | 0.020 | 0.034 | 0.556 |  |  |  |
|  | rs11803128 | G | A | 0.041 | 0.006 | 6.85E-11 | 0.058 | 0.031 | 0.060 |  |  |  |
|  | rs118166957 | T | C | 0.068 | 0.008 | 1.95E-16 | -0.020 | 0.033 | 0.540 |  |  |  |
|  | rs12310246 | A | G | 0.045 | 0.007 | 4.74E-11 | -0.051 | 0.033 | 0.120 |  |  |  |
|  | rs12520974 | C | T | 0.036 | 0.006 | 1.69E-09 | 0.030 | 0.033 | 0.354 |  |  |  |
|  | rs12605642 | T | G | 0.035 | 0.006 | 2.13E-09 | -0.073 | 0.029 | 0.014 |  |  |  |
|  | rs12666306 | A | G | 0.042 | 0.006 | 2.24E-12 | -0.020 | 0.036 | 0.574 |  |  |  |
|  | rs1289939 | C | T | 0.041 | 0.007 | 6.00E-09 | 0.051 | 0.036 | 0.155 |  |  |  |
|  | rs12912299 | C | T | 0.043 | 0.006 | 4.42E-13 | 0.041 | 0.028 | 0.139 |  |  |  |
|  | rs13010288 | G | T | 0.060 | 0.009 | 9.26E-12 | 0.010 | 0.059 | 0.865 | rs2675025 | T | G |
|  | rs13135092 | G | A | 0.089 | 0.011 | 2.53E-16 | -0.105 | 0.056 | 0.062 |  |  |  |
|  | rs13138995 | A | G | 0.034 | 0.006 | 1.97E-08 | 0.010 | 0.021 | 0.635 |  |  |  |
|  | rs1530938 | A | G | 0.036 | 0.006 | 8.82E-10 | 0.039 | 0.026 | 0.137 |  |  |  |
|  | rs1567084 | A | G | 0.033 | 0.006 | 2.14E-08 | -0.010 | 0.027 | 0.711 |  |  |  |
|  | rs1580173 | A | G | 0.033 | 0.006 | 2.28E-08 | -0.058 | 0.028 | 0.038 |  |  |  |
|  | rs1620977 | A | G | 0.052 | 0.007 | 2.27E-14 | -0.010 | 0.051 | 0.843 |  |  |  |
|  | rs16903122 | T | C | 0.055 | 0.007 | 9.04E-16 | -0.073 | 0.038 | 0.053 |  |  |  |
|  | rs17005118 | A | G | 0.042 | 0.007 | 6.13E-10 | 0.010 | 0.022 | 0.644 |  |  |  |
|  | rs17025198 | A | G | 0.041 | 0.007 | 2.19E-08 | 0.049 | 0.032 | 0.128 |  |  |  |
|  | rs17083297 | C | A | 0.044 | 0.008 | 1.60E-08 | 0.030 | 0.034 | 0.376 |  |  |  |
|  | rs17223714 | A | G | 0.046 | 0.007 | 2.44E-10 | 0.010 | 0.048 | 0.836 |  |  |  |
|  | rs17367725 | C | T | 0.036 | 0.006 | 9.29E-09 | -0.010 | 0.023 | 0.666 |  |  |  |
|  | rs17520265 | G | A | 0.091 | 0.016 | 2.87E-08 | -0.140 | 0.086 | 0.106 |  |  |  |
|  | rs17643634 | C | T | 0.060 | 0.008 | 1.34E-13 | 0.030 | 0.051 | 0.550 |  |  |  |
|  | rs176644 | T | G | 0.035 | 0.006 | 9.49E-09 | -0.010 | 0.055 | 0.855 |  |  |  |
|  | rs1927902 | T | C | 0.053 | 0.007 | 1.15E-14 | 0.010 | 0.039 | 0.797 |  |  |  |
|  | rs2089358 | C | T | 0.041 | 0.007 | 2.75E-10 | -0.010 | 0.046 | 0.828 |  |  |  |
|  | rs224029 | C | T | 0.039 | 0.006 | 2.51E-10 | 0.030 | 0.027 | 0.256 |  |  |  |
|  | rs2286729 | A | G | 0.070 | 0.011 | 5.37E-11 | -0.051 | 0.064 | 0.422 |  |  |  |
|  | rs2364921 | C | T | 0.034 | 0.006 | 2.13E-08 | 0.010 | 0.025 | 0.689 |  |  |  |
|  | rs2389631 | C | A | 0.040 | 0.006 | 2.03E-10 | 0.020 | 0.036 | 0.584 |  |  |  |
|  | rs2431108 | C | T | 0.053 | 0.006 | 7.83E-17 | -0.020 | 0.027 | 0.455 |  |  |  |
|  | rs2598293 | T | C | 0.035 | 0.006 | 2.48E-09 | 0.058 | 0.029 | 0.044 |  |  |  |
|  | rs2838787 | G | A | 0.036 | 0.006 | 7.65E-09 | -0.020 | 0.030 | 0.512 |  |  |  |
|  | rs28552587 | A | G | 0.033 | 0.006 | 3.30E-08 | 0.041 | 0.032 | 0.204 |  |  |  |
|  | rs28582096 | G | A | 0.054 | 0.007 | 1.74E-13 | 0.030 | 0.043 | 0.477 |  |  |  |
|  | rs28611339 | T | G | 0.058 | 0.009 | 8.46E-11 | 0.030 | 0.041 | 0.476 |  |  |  |
|  | rs314281 | C | T | 0.043 | 0.006 | 6.03E-13 | 0.020 | 0.036 | 0.573 | rs314291 | T | C |
|  | rs3184470 | G | A | 0.038 | 0.006 | 9.73E-10 | -0.010 | 0.023 | 0.667 |  |  |  |
|  | rs324017 | A | C | 0.039 | 0.007 | 1.61E-09 | 0.030 | 0.030 | 0.325 |  |  |  |
|  | rs34967082 | A | G | 0.035 | 0.006 | 4.34E-09 | 0.049 | 0.031 | 0.117 |  |  |  |
|  | rs35110063 | A | G | 0.039 | 0.006 | 8.82E-11 | 0.010 | 0.021 | 0.639 |  |  |  |
|  | rs35322724 | A | C | 0.049 | 0.006 | 3.75E-16 | -0.049 | 0.029 | 0.094 |  |  |  |
|  | rs35539975 | A | G | 0.042 | 0.007 | 4.49E-09 | 0.030 | 0.031 | 0.329 |  |  |  |
|  | rs3774751 | G | T | 0.041 | 0.006 | 7.32E-12 | 0.051 | 0.029 | 0.082 |  |  |  |
|  | rs4502882 | C | T | 0.039 | 0.006 | 7.96E-10 | -0.041 | 0.030 | 0.174 |  |  |  |
|  | rs4588900 | A | G | 0.033 | 0.006 | 1.57E-08 | 0.020 | 0.034 | 0.551 |  |  |  |
|  | rs4592425 | T | G | 0.040 | 0.006 | 4.31E-10 | -0.030 | 0.028 | 0.291 |  |  |  |
|  | rs4664299 | C | T | 0.041 | 0.007 | 4.95E-09 | 0.041 | 0.033 | 0.218 |  |  |  |
|  | rs4702 | G | A | 0.048 | 0.006 | 6.78E-16 | 0.068 | 0.028 | 0.015 |  |  |  |
|  | rs4709655 | C | T | 0.054 | 0.009 | 3.09E-09 | 0.010 | 0.041 | 0.807 |  |  |  |
|  | rs4767645 | G | T | 0.037 | 0.006 | 6.47E-10 | -0.030 | 0.025 | 0.244 |  |  |  |
|  | rs4788203 | G | A | 0.035 | 0.006 | 6.32E-09 | 0.020 | 0.026 | 0.438 |  |  |  |
|  | rs492858 | C | T | 0.066 | 0.011 | 3.46E-09 | -0.049 | 0.054 | 0.366 |  |  |  |
|  | rs4981170 | G | A | 0.054 | 0.008 | 7.33E-13 | -0.030 | 0.041 | 0.465 |  |  |  |
|  | rs521484 | G | A | 0.040 | 0.007 | 1.53E-08 | 0.049 | 0.034 | 0.156 |  |  |  |
|  | rs524859 | G | A | 0.044 | 0.006 | 1.48E-12 | -0.020 | 0.037 | 0.590 |  |  |  |
|  | rs55772859 | A | C | 0.042 | 0.006 | 4.82E-11 | -0.073 | 0.029 | 0.014 |  |  |  |
|  | rs55972276 | A | C | 0.073 | 0.009 | 4.19E-17 | 0.010 | 0.067 | 0.883 | rs34201897 | A | C |
|  | rs6019663 | T | C | 0.040 | 0.007 | 6.47E-10 | -0.010 | 0.050 | 0.841 |  |  |  |
|  | rs60565673 | G | T | 0.043 | 0.006 | 1.59E-12 | -0.051 | 0.032 | 0.108 |  |  |  |
|  | rs61921611 | C | T | 0.044 | 0.006 | 7.84E-12 | -0.041 | 0.036 | 0.253 |  |  |  |
|  | rs62068188 | T | C | 0.049 | 0.008 | 1.18E-09 | 0.051 | 0.043 | 0.231 |  |  |  |
|  | rs62158170 | A | G | 0.066 | 0.007 | 1.20E-19 | 0.010 | 0.028 | 0.724 |  |  |  |
|  | rs62264767 | A | C | 0.065 | 0.008 | 1.63E-14 | 0.010 | 0.065 | 0.877 | rs6795060 | C | A |
|  | rs62383308 | G | A | 0.060 | 0.011 | 3.98E-08 | 0.020 | 0.052 | 0.698 |  |  |  |
|  | rs62429521 | A | C | 0.051 | 0.008 | 1.78E-09 | 0.010 | 0.059 | 0.867 |  |  |  |
|  | rs638746 | A | G | 0.033 | 0.006 | 2.26E-08 | 0.010 | 0.054 | 0.853 |  |  |  |
|  | rs6465151 | T | C | 0.056 | 0.009 | 1.90E-09 | 0.030 | 0.050 | 0.555 |  |  |  |
|  | rs647905 | T | C | 0.033 | 0.006 | 2.87E-08 | 0.010 | 0.020 | 0.624 |  |  |  |
|  | rs6510033 | G | A | 0.037 | 0.007 | 4.66E-08 | -0.051 | 0.034 | 0.134 |  |  |  |
|  | rs6562066 | T | C | 0.039 | 0.006 | 1.38E-10 | 0.020 | 0.028 | 0.473 |  |  |  |
|  | rs6589988 | G | A | 0.038 | 0.006 | 4.70E-09 | 0.010 | 0.033 | 0.765 |  |  |  |
|  | rs6601080 | A | G | 0.035 | 0.006 | 2.21E-08 | -0.010 | 0.031 | 0.746 |  |  |  |
|  | rs6702604 | G | A | 0.037 | 0.006 | 1.30E-09 | 0.010 | 0.034 | 0.771 |  |  |  |
|  | rs671985 | G | A | 0.038 | 0.006 | 2.79E-10 | 0.062 | 0.031 | 0.049 |  |  |  |
|  | rs6808140 | T | C | 0.039 | 0.006 | 5.35E-11 | 0.049 | 0.027 | 0.076 |  |  |  |
|  | rs6888135 | A | C | 0.038 | 0.006 | 1.21E-10 | 0.020 | 0.027 | 0.463 |  |  |  |
|  | rs694786 | C | T | 0.044 | 0.006 | 1.97E-13 | 0.010 | 0.054 | 0.851 |  |  |  |
|  | rs6967168 | G | T | 0.044 | 0.007 | 1.39E-10 | 0.020 | 0.044 | 0.656 |  |  |  |
|  | rs6973090 | G | A | 0.038 | 0.007 | 4.31E-08 | -0.010 | 0.039 | 0.800 | rs1047998 | A | G |
|  | rs699844 | A | G | 0.060 | 0.011 | 4.11E-08 | -0.049 | 0.056 | 0.385 | rs17095322 | G | A |
|  | rs701394 | G | A | 0.036 | 0.006 | 6.83E-09 | -0.030 | 0.026 | 0.242 |  |  |  |
|  | rs7040224 | A | G | 0.037 | 0.006 | 4.24E-09 | 0.068 | 0.031 | 0.027 |  |  |  |
|  | rs715338 | A | G | 0.041 | 0.006 | 7.85E-12 | -0.030 | 0.033 | 0.374 |  |  |  |
|  | rs7214267 | G | A | 0.044 | 0.006 | 5.09E-13 | -0.062 | 0.030 | 0.036 |  |  |  |
|  | rs72657797 | C | T | 0.056 | 0.008 | 1.52E-12 | -0.030 | 0.034 | 0.383 |  |  |  |
|  | rs728017 | G | A | 0.035 | 0.006 | 9.51E-09 | -0.030 | 0.029 | 0.314 |  |  |  |
|  | rs72820274 | A | G | 0.034 | 0.006 | 1.28E-08 | 0.010 | 0.024 | 0.684 |  |  |  |
|  | rs72899452 | T | C | 0.074 | 0.012 | 1.00E-09 | 0.020 | 0.056 | 0.725 |  |  |  |
|  | rs7402939 | C | T | 0.036 | 0.006 | 5.19E-09 | -0.020 | 0.040 | 0.617 |  |  |  |
|  | rs7571486 | G | A | 0.039 | 0.007 | 1.40E-08 | 0.020 | 0.040 | 0.614 |  |  |  |
|  | rs76145129 | G | T | 0.050 | 0.009 | 2.73E-08 | 0.062 | 0.046 | 0.182 |  |  |  |
|  | rs769449 | G | A | 0.046 | 0.008 | 2.13E-08 | -0.010 | 0.041 | 0.806 |  |  |  |
|  | rs77641763 | T | C | 0.071 | 0.009 | 6.53E-15 | -0.051 | 0.045 | 0.256 |  |  |  |
|  | rs8076183 | C | T | 0.038 | 0.006 | 2.75E-10 | -0.030 | 0.028 | 0.287 |  |  |  |
|  | rs8180457 | C | T | 0.056 | 0.008 | 1.12E-11 | 0.051 | 0.038 | 0.181 |  |  |  |
|  | rs823247 | C | T | 0.037 | 0.006 | 5.25E-10 | -0.020 | 0.033 | 0.550 |  |  |  |
|  | rs871994 | A | C | 0.035 | 0.006 | 5.50E-09 | 0.010 | 0.036 | 0.781 |  |  |  |
|  | rs908668 | T | C | 0.050 | 0.007 | 1.41E-11 | -0.051 | 0.035 | 0.148 |  |  |  |
|  | rs910187 | G | A | 0.035 | 0.006 | 1.63E-08 | 0.020 | 0.039 | 0.608 |  |  |  |
|  | rs9527083 | G | A | 0.076 | 0.006 | 1.61E-32 | 0.049 | 0.029 | 0.097 |  |  |  |
|  | rs9889282 | C | A | 0.042 | 0.006 | 4.70E-12 | 0.010 | 0.043 | 0.815 |  |  |  |
|  | rs9931543 | T | C | 0.048 | 0.007 | 1.11E-12 | 0.010 | 0.038 | 0.794 |  |  |  |
|  | rs9964420 | A | C | 0.035 | 0.007 | 4.54E-08 | -0.010 | 0.028 | 0.721 |  |  |  |
| Daytime sleepiness | rs11078398 | G | A | 0.008 | 0.001 | 7.10E-10 | -0.020 | 0.034 | 0.561 | rs11868035 | A | G |
|  | rs11123962 | T | G | -0.008 | 0.001 | 7.50E-15 | -0.020 | 0.027 | 0.462 | rs60705333 | G | T |
|  | rs11942333 | G | A | -0.006 | 0.001 | 3.80E-08 | 0.030 | 0.024 | 0.209 |  |  |  |
|  | rs12140153 | T | G | 0.017 | 0.002 | 2.80E-20 | -0.010 | 0.071 | 0.887 |  |  |  |
|  | rs13010456 | A | G | 0.008 | 0.001 | 2.10E-13 | 0.062 | 0.031 | 0.044 |  |  |  |
|  | rs13097760 | A | C | -0.006 | 0.001 | 3.20E-08 | 0.020 | 0.026 | 0.445 |  |  |  |
|  | rs13135092 | A | G | -0.010 | 0.002 | 3.10E-08 | 0.105 | 0.056 | 0.062 |  |  |  |
|  | rs1566362 | T | C | 0.006 | 0.001 | 3.80E-09 | -0.030 | 0.028 | 0.299 |  |  |  |
|  | rs17356118 | A | G | -0.008 | 0.001 | 2.60E-10 | -0.010 | 0.042 | 0.813 |  |  |  |
|  | rs1846644 | T | C | -0.011 | 0.001 | 2.50E-27 | -0.030 | 0.030 | 0.329 |  |  |  |
|  | rs2787120 | A | G | 0.008 | 0.001 | 2.00E-08 | 0.010 | 0.052 | 0.847 | rs1746662 | G | A |
|  | rs285793 | G | A | 0.007 | 0.001 | 7.90E-11 | 0.030 | 0.029 | 0.316 |  |  |  |
|  | rs3122170 | C | A | 0.010 | 0.001 | 5.60E-15 | -0.020 | 0.031 | 0.517 |  |  |  |
|  | rs4665972 | T | C | 0.007 | 0.001 | 3.90E-10 | 0.058 | 0.029 | 0.044 |  |  |  |
|  | rs55818482 | T | C | -0.010 | 0.001 | 1.40E-14 | 0.030 | 0.039 | 0.439 |  |  |  |
|  | rs55960940 | T | C | 0.008 | 0.001 | 2.00E-08 | 0.020 | 0.030 | 0.504 |  |  |  |
|  | rs62519825 | T | C | -0.009 | 0.002 | 3.80E-09 | -0.068 | 0.045 | 0.135 |  |  |  |
|  | rs6741951 | A | G | 0.007 | 0.001 | 2.70E-09 | -0.020 | 0.033 | 0.540 |  |  |  |
|  | rs6897863 | A | C | 0.006 | 0.001 | 7.60E-10 | 0.030 | 0.030 | 0.308 |  |  |  |
|  | rs6923811 | T | C | 0.007 | 0.001 | 9.10E-10 | -0.174 | 0.031 | 2.833E-08 |  |  |  |
|  | rs7476897 | G | A | 0.007 | 0.001 | 2.70E-11 | -0.068 | 0.031 | 0.029 |  |  |  |
|  | rs7598712 | G | T | 0.006 | 0.001 | 2.20E-08 | 0.010 | 0.027 | 0.706 |  |  |  |
|  | rs7607363 | A | G | -0.006 | 0.001 | 8.00E-09 | 0.010 | 0.033 | 0.762 | rs10174168 | G | A |
|  | rs7837226 | A | G | -0.006 | 0.001 | 2.00E-08 | 0.049 | 0.030 | 0.103 |  |  |  |
|  | rs8015449 | A | G | 0.006 | 0.001 | 1.90E-09 | 0.010 | 0.054 | 0.852 | rs8019553 | G | A |
|  | rs825127 | T | G | 0.006 | 0.001 | 9.50E-09 | 0.020 | 0.035 | 0.568 |  |  |  |
|  | rs843372 | C | T | 0.008 | 0.001 | 2.20E-11 | -0.020 | 0.030 | 0.498 |  |  |  |
|  | rs886114 | C | T | 0.006 | 0.001 | 1.90E-08 | 0.039 | 0.032 | 0.215 |  |  |  |
|  | rs960986 | C | T | 0.007 | 0.001 | 1.50E-11 | -0.030 | 0.034 | 0.383 |  |  |  |
|  | rs9712275 | C | T | -0.006 | 0.001 | 1.30E-08 | -0.094 | 0.032 | 0.003 |  |  |  |
|  |  |  |  |  |  |  |  |  |  |  |  |  |

**Supplementary Table 10.** Manually detected potential pleiotropy in the PhenoScanner database.

| **SNP** | **Potential pleiotropic trait** | **Effect allele** | **Other allele** | ***β*** | ***P* value** |
| --- | --- | --- | --- | --- | --- |
| Chronotype to SLE |  |  |  |  |  |
| rs10058356 | - | T | C |  |  |
| rs10175975 | - | T | C |  |  |
| rs10237162 | - | T | C |  |  |
| rs10402849 | - | T | C |  |  |
| rs10520176 | - | T | C |  |  |
| rs1061032 | - | T | G |  |  |
| rs1064213 | - | A | G |  |  |
| rs10742179 | - | A | G |  |  |
| rs10832648 | - | A | C |  |  |
| rs10916892 | - | T | C |  |  |
| rs10951325 | - | T | C |  |  |
| rs10988239 | - | T | C |  |  |
| rs11032362 | - | A | G |  |  |
| rs11152350 | - | A | C |  |  |
| rs113851554 | - | T | G |  |  |
| rs1144566 | - | T | C |  |  |
| rs11545787 | - | A | G |  |  |
| rs11588913 | - | A | G |  |  |
| rs11670534 | - | T | C |  |  |
| rs12040629 | - | A | G |  |  |
| rs12140153 | - | T | G |  |  |
| rs12298405 | - | T | C |  |  |
| rs12636669 | - | T | C |  |  |
| rs12808544 | - | A | C |  |  |
| rs12969848 | - | T | C |  |  |
| rs13065394 | - | T | G |  |  |
| rs13377754 | - | T | C |  |  |
| rs139911 | - | T | C |  |  |
| rs1421085 | Alcohol intake frequency | T | C | 0.0221 | 1.07E-09 |
| rs1468945 | - | A | G |  |  |
| rs1599374 | - | A | G |  |  |
| rs16939162 | - | A | G |  |  |
| rs17302081 | - | T | C |  |  |
| rs17448682 | - | T | C |  |  |
| rs17575798 | - | A | G |  |  |
| rs17604349 | - | A | G |  |  |
| rs1886205 | - | A | C |  |  |
| rs2011528 | - | T | C |  |  |
| rs2072727 | - | T | C |  |  |
| rs2166559 | - | T | C |  |  |
| rs2362775 | - | T | C |  |  |
| rs28458909 | - | T | C |  |  |
| rs2881955 | - | T | C |  |  |
| rs2916148 | - | A | G |  |  |
| rs2944831 | - | A | G |  |  |
| rs2979139 | - | A | G |  |  |
| rs308521 | - | T | C |  |  |
| rs3100052 | - | A | G |  |  |
| rs3808964 | - | T | G |  |  |
| rs4241964 | - | T | G |  |  |
| rs4269995 | - | T | C |  |  |
| rs4419127 | - | A | G |  |  |
| rs4690085 | - | A | G |  |  |
| rs486416 | - | A | G |  |  |
| rs4936290 | - | A | C |  |  |
| rs55846845 | - | A | G |  |  |
| rs60616179 | - | A | G |  |  |
| rs6131805 | - | T | G |  |  |
| rs6131942 | - | A | G |  |  |
| rs61773390 | - | T | G |  |  |
| rs62082402 | - | T | G |  |  |
| rs62182135 | - | A | C |  |  |
| rs6477309 | - | T | C |  |  |
| rs6727752 | - | A | G |  |  |
| rs6794796 | - | A | G |  |  |
| rs6846730 | - | T | C |  |  |
| rs6993892 | - | T | C |  |  |
| rs7111582 | - | A | G |  |  |
| rs7203707 | - | A | C |  |  |
| rs72720396 | Average weekly beer plus cider intake | A | G | -0.01307 | 1.13E-12 |
| rs72829706 | - | A | G |  |  |
| rs7304278 | - | A | G |  |  |
| rs73050286 | - | T | C |  |  |
| rs7429614 | - | T | G |  |  |
| rs74357745 | - | A | G |  |  |
| rs75120545 | - | T | C |  |  |
| rs77960 | - | A | G |  |  |
| rs7845620 | - | A | C |  |  |
| rs7959983 | - | T | C |  |  |
| rs80097534 | - | T | G |  |  |
| rs80271258 | - | T | C |  |  |
| rs9348050 | - | T | C |  |  |
| rs9381812 | - | A | G |  |  |
| rs9416744 | - | A | C |  |  |
| rs9436119 | - | A | G |  |  |
| rs9479402 | - | T | C |  |  |
| rs9573980 | - | A | G |  |  |
| rs9597241 | - | A | C |  |  |
| rs962961 | - | T | C |  |  |
| rs9636202 | - | A | G |  |  |
| rs975025 | - | T | C |  |  |
| rs9964420 | - | A | C |  |  |
| Sleep duration to SLE |  |  |  |  |  |
| rs10173260 | - | C | T |  |  |
| rs10483350 | - | G | A |  |  |
| rs1057703 | - | G | T |  |  |
| rs10761674 | - | C | T |  |  |
| rs10973207 | - | T | G |  |  |
| rs11190970 | - | G | A |  |  |
| rs112230981 | - | A | G |  |  |
| rs113113059 | - | T | C |  |  |
| rs11567976 | - | T | C |  |  |
| rs11602180 | - | C | T |  |  |
| rs11621908 | - | C | T |  |  |
| rs11885663 | - | T | C |  |  |
| rs12246842 | - | A | G |  |  |
| rs12567114 | - | A | G |  |  |
| rs12607679 | - | T | C |  |  |
| rs13088093 | - | G | T |  |  |
| rs13109404 | - | T | G |  |  |
| rs151014368 | - | A | G |  |  |
| rs1517572 | - | C | A |  |  |
| rs1553132 | - | G | A |  |  |
| rs17427571 | - | A | G |  |  |
| rs174560 | - | C | T |  |  |
| rs1776776 | - | T | C |  |  |
| rs1939455 | - | G | T |  |  |
| rs1991556 | Alcohol intake frequency | G | A | 0.02591 | 1.19E-09 |
| rs205024 | - | T | C |  |  |
| rs2072727 | - | T | C |  |  |
| rs2192528 | - | A | G |  |  |
| rs2231265 | - | G | A |  |  |
| rs3095508 | - | C | A |  |  |
| rs34354917 | - | C | A |  |  |
| rs34556183 | - | A | G |  |  |
| rs34731055 | - | T | C |  |  |
| rs35531607 | - | C | T |  |  |
| rs365663 | - | A | G |  |  |
| rs374153 | - | C | T |  |  |
| rs4128364 | - | C | T |  |  |
| rs4592416 | - | G | A |  |  |
| rs4767550 | - | G | A |  |  |
| rs55658675 | - | C | T |  |  |
| rs61796569 | - | T | C |  |  |
| rs61985058 | - | T | C |  |  |
| rs62120041 | - | T | C |  |  |
| rs62362521 | - | G | A |  |  |
| rs6575005 | - | T | C |  |  |
| rs72804080 | - | G | A |  |  |
| rs73219758 | - | G | A |  |  |
| rs75539574 | - | C | A |  |  |
| rs7556815 | - | A | G |  |  |
| rs7644809 | - | T | C |  |  |
| rs7806045 | - | T | C |  |  |
| rs7915425 | - | T | C |  |  |
| rs8038326 | - | A | G |  |  |
| rs9345234 | - | C | A |  |  |
| rs9382445 | - | T | C |  |  |
| Short sleep duration to SLE |  |  |  |  |  |
| rs11763750 | - | G | A |  |  |
| rs1229762 | - | T | C |  |  |
| rs12518468 | - | C | T |  |  |
| rs12567114 | - | G | A |  |  |
| rs12661667 | - | T | C |  |  |
| rs12963463 | - | C | T |  |  |
| rs13107325 | Alcohol intake frequency | T | C | 0.05053 | 7.88E-14 |
| rs1380703 | - | G | A |  |  |
| rs17005118 | - | A | G |  |  |
| rs17388803 | - | C | A |  |  |
| rs2014830 | - | C | T |  |  |
| rs205024 | - | C | T |  |  |
| rs2820313 | - | G | A |  |  |
| rs2863957 | - | C | A |  |  |
| rs3776864 | - | A | C |  |  |
| rs4585442 | - | G | A |  |  |
| rs5757675 | - | G | T |  |  |
| rs59779556 | - | T | G |  |  |
| rs7524118 | - | C | T |  |  |
| rs7939345 | - | T | G |  |  |
| rs9321171 | - | C | T |  |  |
| Long sleep duration to SLE |  |  |  |  |  |
| rs10899257 | - | A | G |  |  |
| rs12145723 | - | C | T |  |  |
| rs17817288 | - | A | G |  |  |
| rs3751046 | - | G | A |  |  |
| rs6737318 | - | G | A |  |  |
| rs75458655 | - | T | C |  |  |
| Insomnia to SLE |  |  |  |  |  |
| rs1015438 | - | A | G |  |  |
| rs1031654 | - | C | A |  |  |
| rs1038093 | - | T | C |  |  |
| rs10756571 | - | T | C |  |  |
| rs10758593 | - | G | A |  |  |
| rs10761240 | - | G | A |  |  |
| rs10800992 | - | T | C |  |  |
| rs10898940 | - | A | C |  |  |
| rs10944696 | - | G | A |  |  |
| rs10947428 | - | C | T |  |  |
| rs10947690 | - | G | A |  |  |
| rs10955647 | - | T | G |  |  |
| rs11090039 | - | A | G |  |  |
| rs11119409 | - | C | T |  |  |
| rs11149313 | - | A | G |  |  |
| rs113851554 | - | T | G |  |  |
| rs1147852 | - | A | G |  |  |
| rs11588755 | - | G | A |  |  |
| rs11605348 | Alcohol intake frequency | G | A | 0.02178 | 5.36E-09 |
| rs116466468 | - | T | C |  |  |
| rs1167132 | - | T | C |  |  |
| rs11803128 | - | G | A |  |  |
| rs118166957 | - | T | C |  |  |
| rs12310246 | - | A | G |  |  |
| rs12520974 | - | C | T |  |  |
| rs12605642 | - | T | G |  |  |
| rs12666306 | Current tobacco smoking | A | G | 0.008542 | 1.23E-10 |
| rs1289939 | - | C | T |  |  |
| rs12912299 | - | C | T |  |  |
| rs13010288 | - | G | T |  |  |
| rs13135092 | Alcohol intake frequency | G | A | -0.04975 | 1.48E-14 |
| rs13138995 | - | A | G |  |  |
| rs1530938 | - | A | G |  |  |
| rs1567084 | - | A | G |  |  |
| rs1580173 | - | A | G |  |  |
| rs1620977 | - | A | G |  |  |
| rs16903122 | - | T | C |  |  |
| rs17005118 | - | A | G |  |  |
| rs17025198 | - | A | G |  |  |
| rs17083297 | - | C | A |  |  |
| rs17223714 | - | A | G |  |  |
| rs17367725 | - | C | T |  |  |
| rs17520265 | - | G | A |  |  |
| rs17643634 | - | C | T |  |  |
| rs176644 | Ever smoked | T | G | 0.007223 | 2.84E-09 |
| rs1927902 | - | T | C |  |  |
| rs2089358 | - | C | T |  |  |
| rs224029 | - | C | T |  |  |
| rs2286729 | - | A | G |  |  |
| rs2364921 | - | C | T |  |  |
| rs2389631 | - | C | A |  |  |
| rs2431108 | - | C | T |  |  |
| rs2598293 | - | T | C |  |  |
| rs2838787 | - | G | A |  |  |
| rs28552587 | - | A | G |  |  |
| rs28582096 | - | G | A |  |  |
| rs28611339 | - | T | G |  |  |
| rs314281 | - | C | T |  |  |
| rs3184470 | - | G | A |  |  |
| rs324017 | - | A | C |  |  |
| rs34967082 | - | A | G |  |  |
| rs35110063 | - | A | G |  |  |
| rs35322724 | - | A | C |  |  |
| rs35539975 | - | A | G |  |  |
| rs3774751 | Average weekly beer plus cider intake | G | T | 0.01101 | 4.02E-08 |
| rs4502882 | - | C | T |  |  |
| rs4588900 | - | A | G |  |  |
| rs4592425 | - | T | G |  |  |
| rs4664299 | - | C | T |  |  |
| rs4702 | - | G | A |  |  |
| rs4709655 | - | C | T |  |  |
| rs4767645 | - | G | T |  |  |
| rs4788203 | - | G | A |  |  |
| rs492858 | - | C | T |  |  |
| rs4981170 | - | G | A |  |  |
| rs521484 | - | G | A |  |  |
| rs524859 | - | G | A |  |  |
| rs55772859 | - | A | C |  |  |
| rs55972276 | - | A | C |  |  |
| rs6019663 | - | T | C |  |  |
| rs60565673 | - | G | T |  |  |
| rs61921611 | - | C | T |  |  |
| rs62068188 | - | T | C |  |  |
| rs62158170 | - | A | G |  |  |
| rs62264767 | Past tobacco smoking | A | C | -0.02851 | 1.48E-10 |
| rs62383308 | - | G | A |  |  |
| rs62429521 | - | A | C |  |  |
| rs638746 | - | A | G |  |  |
| rs6465151 | - | T | C |  |  |
| rs647905 | - | T | C |  |  |
| rs6510033 | - | G | A |  |  |
| rs6562066 | - | T | C |  |  |
| rs6589988 | - | G | A |  |  |
| rs6601080 | - | A | G |  |  |
| rs6702604 | - | G | A |  |  |
| rs671985 | - | G | A |  |  |
| rs6808140 | - | T | C |  |  |
| rs6888135 | - | A | C |  |  |
| rs694786 | - | C | T |  |  |
| rs6967168 | - | G | T |  |  |
| rs6973090 | - | G | A |  |  |
| rs699844 | - | A | G |  |  |
| rs701394 | - | G | A |  |  |
| rs7040224 | - | A | G |  |  |
| rs715338 | - | A | G |  |  |
| rs7214267 | - | G | A |  |  |
| rs72657797 | - | C | T |  |  |
| rs728017 | - | G | A |  |  |
| rs72820274 | Past tobacco smoking | A | G | -0.01832 | 1.23E-08 |
| rs72899452 | - | T | C |  |  |
| rs7402939 | - | C | T |  |  |
| rs7571486 | - | G | A |  |  |
| rs76145129 | - | G | T |  |  |
| rs769449 | - | G | A |  |  |
| rs77641763 | - | T | C |  |  |
| rs8076183 | - | C | T |  |  |
| rs8180457 | - | C | T |  |  |
| rs823247 | - | C | T |  |  |
| rs871994 | - | A | C |  |  |
| rs908668 | - | T | C |  |  |
| rs910187 | - | G | A |  |  |
| rs9527083 | - | G | A |  |  |
| rs9889282 | - | C | A |  |  |
| rs9931543 | - | T | C |  |  |
| rs9964420 | - | A | C |  |  |
| Daytime sleepiness to SLE |  |  |  |  |  |
| rs11078398 | - | G | A |  |  |
| rs11123962 | Past tobacco smoking | T | G | -0.02346 | 1.68E-13 |
| rs11942333 | - | G | A |  |  |
| rs12140153 | - | T | G |  |  |
| rs13010456 | - | A | G |  |  |
| rs13097760 | - | A | C |  |  |
| rs13135092 | Alcohol intake frequency | A | G | -0.04975 | 1.48E-14 |
| rs1566362 | - | T | C |  |  |
| rs17356118 | - | A | G |  |  |
| rs1846644 | - | T | C |  |  |
| rs2787120 | - | A | G |  |  |
| rs285793 | - | G | A |  |  |
| rs3122170 | - | C | A |  |  |
| rs4665972 | Alcohol intake frequency | T | C | -0.04621 | 1.29E-36 |
| rs55818482 | - | T | C |  |  |
| rs55960940 | - | T | C |  |  |
| rs62519825 | - | T | C |  |  |
| rs6741951 | - | A | G |  |  |
| rs6897863 | - | A | C |  |  |
| rs6923811 | - | T | C |  |  |
| rs7476897 | - | G | A |  |  |
| rs7598712 | - | G | T |  |  |
| rs7607363 | - | A | G |  |  |
| rs7837226 | - | A | G |  |  |
| rs8015449 | - | A | G |  |  |
| rs825127 | - | T | G |  |  |
| rs843372 | - | C | T |  |  |
| rs886114 | - | C | T |  |  |
| rs960986 | Past tobacco smoking | C | T | 0.02104 | 1.47E-10 |
| rs9712275 | - | C | T |  |  |
| SLE |  |  |  |  |  |
| rs10048743 | - | G | T |  |  |
| rs10200680 | - | T | C |  |  |
| rs1078324 | - | A | C |  |  |
| rs10912578 | - | A | G |  |  |
| rs1143679 | - | A | G |  |  |
| rs12094036 | - | C | T |  |  |
| rs1270942 | - | G | A |  |  |
| rs13019891 | - | T | G |  |  |
| rs13136219 | - | T | C |  |  |
| rs13332649 | - | G | A |  |  |
| rs143810596 | - | G | T |  |  |
| rs1464446 | - | T | G |  |  |
| rs17849501 | - | T | C |  |  |
| rs2431697 | - | C | T |  |  |
| rs2459611 | - | C | T |  |  |
| rs2573219 | - | C | A |  |  |
| rs268124 | - | C | T |  |  |
| rs34703115 | - | C | T |  |  |
| rs35000415 | - | T | C |  |  |
| rs35251378 | - | A | G |  |  |
| rs353608 | - | A | G |  |  |
| rs3747093 | - | A | G |  |  |
| rs4274624 | - | C | T |  |  |
| rs4388254 | - | T | C |  |  |
| rs4661543 | - | T | G |  |  |
| rs4916215 | - | C | T |  |  |
| rs58688157 | - | G | A |  |  |
| rs58721818 | - | T | C |  |  |
| rs597808 | Smoking status: previous | G | A | -0.007525 | 1.13e-10 |
| rs6671847 | - | A | G |  |  |
| rs6679677 | - | A | C |  |  |
| rs6889239 | - | C | T |  |  |
| rs7097397 | - | A | G |  |  |
| rs73050535 | - | T | C |  |  |
| rs73068668 | - | A | G |  |  |
| rs7768653 | - | C | T |  |  |
| rs7823055 | - | G | T |  |  |
| rs7899626 | - | T | C |  |  |
| rs9852014 | - | G | A |  |  |

**Supplementary Table 11.** Heterogeneity test and pleiotropy test after removing SNPs with potential pleiotropy.

| **Exposure/Outcome** | **MR-IVW** | | | **MR-Egger** | | | **MR-Egger intercept** | | | **MR-PRESSO** | |
| --- | --- | --- | --- | --- | --- | --- | --- | --- | --- | --- | --- |
|  | **Q** | **Q_df** | **Q_pval** | **Q** | **Q_df** | **Q_pval** | **Intercept** | **SE** | ***P* val** | **Global test Pval** | **Distortion test Pval** |
| Chronotype/SLE | 313.848 | 89 | 7.93E-27 | 313.7801 | 88 | 4.27E-27 | 2.72E-03 | 1.97E-02 | 0.8903227 | <0.001 | 0.187 |
| Sleep duration/SLE | 104.345 | 53 | 3.30E-05 | 104.1406 | 52 | 2.41E-05 | 5.26E-03 | 1.65E-02 | 0.7507321 | <0.001 | 0.583 |
| Short sleep duration/SLE | 40.271 | 19 | 3.01E-03 | 36.44749 | 18 | 6.18E-03 | 7.83E-02 | 5.70E-02 | 0.1862773 | 0.002 | NA |
| Long sleep duration/SLE* |  |  |  |  |  |  |  |  |  |  |  |
| Insomnia/SLE | 145.487 | 117 | 3.82E-02 | 144.8171 | 116 | 3.61E-02 | 9.23E-03 | 1.26E-02 | 0.4654489 | 0.065 | NA |
| Daytime sleepiness/SLE | 64.092 | 25 | 2.79E-05 | 63.59708 | 24 | 1.95E-05 | -1.98E-02 | 4.59E-02 | 0.6695741 | <0.001 | 0.177 |
| SLE/Chronotype | 88.401 | 37 | 4.29E-06 | 82.67143 | 36 | 1.56E-05 | -2.91E-03 | 1.84E-03 | 0.1229551 | <0.001 | NA |
| SLE/Sleep duration | 73.858 | 37 | 3.00E-04 | 73.31078 | 36 | 2.37E-04 | 7.57E-04 | 1.46E-03 | 0.6073706 | <0.001 | NA |
| SLE/Short sleep duration | 65.595 | 37 | 2.59E-03 | 64.52917 | 36 | 2.43E-03 | -4.47E-04 | 5.80E-04 | 0.4457701 | 0.005 | NA |
| SLE/Long sleep duration | 38.90 | 37 | 0.3843569 | 38.89396 | 36 | 0.3407259 | -1.59E-05 | 3.41E-04 | 0.9630232 | 0.346 | NA |
| SLE/Insomnia | 81.506 | 37 | 3.43E-05 | 79.33306 | 36 | 4.22E-05 | 9.21E-04 | 9.28E-04 | 0.3273445 | <0.001 | 0.68 |
| SLE/Daytime sleepiness | 65.595 | 37 | 2.59E-03 | 64.52917 | 36 | 2.43E-03 | -4.47E-04 | 5.80E-04 | 0.4457701 | 0.005 | NA |
| NA, no outliers were detected. | | | | | | | | | | | |

*No SNPs with potential pleiotropy were identified.

**Supplementary Figure S1 Forest plot (A), sensitivity analysis (B), scatter plot (C) and funnel plot (D) of the causal effect of Sleep duration on SLE risk。**

**
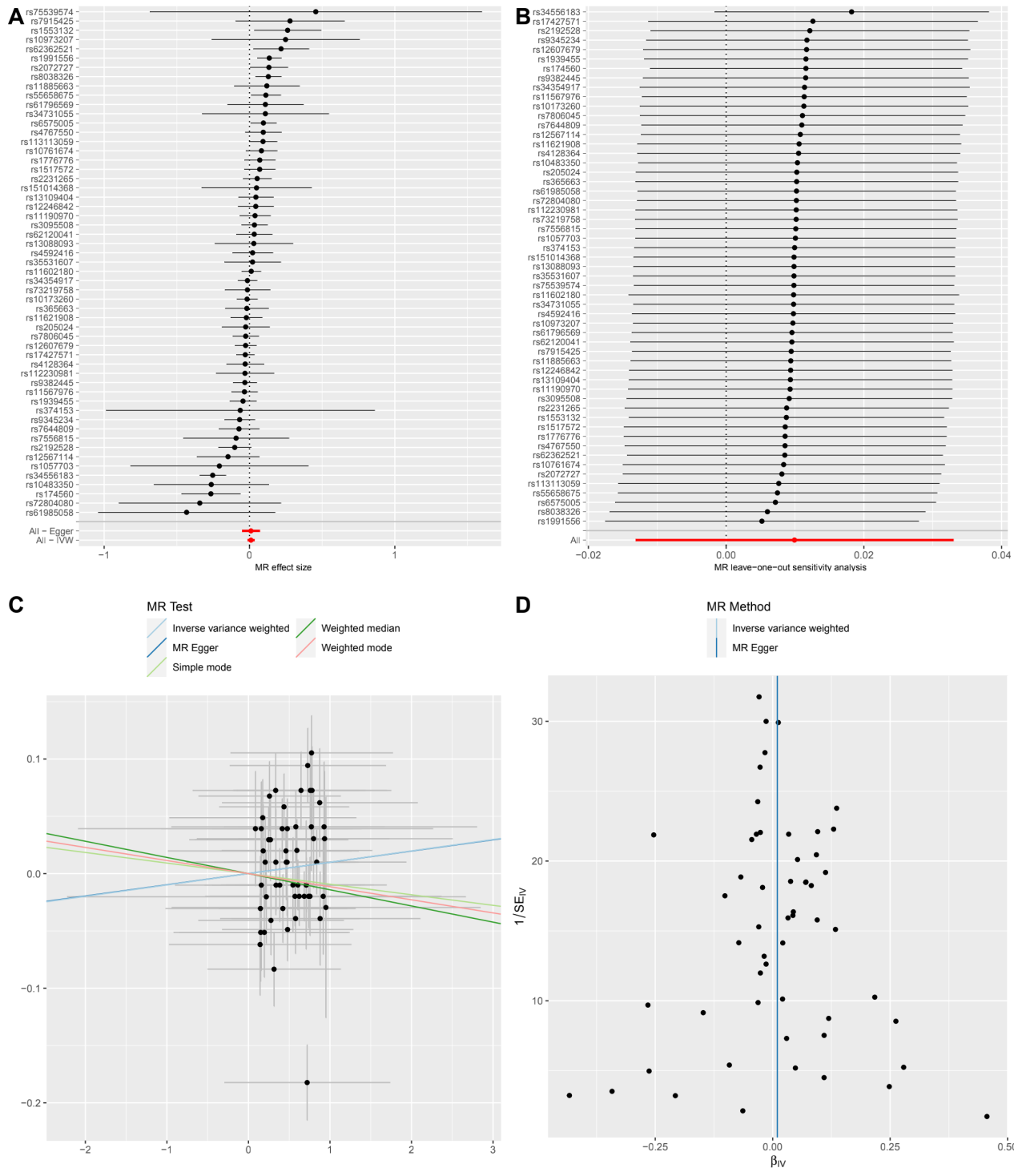
**

**Supplementary Figure S2 Forest plot (A), sensitivity analysis (B), scatter plot (C) and funnel plot (D) of the causal effect of Short sleep duration on SLE risk。**

**
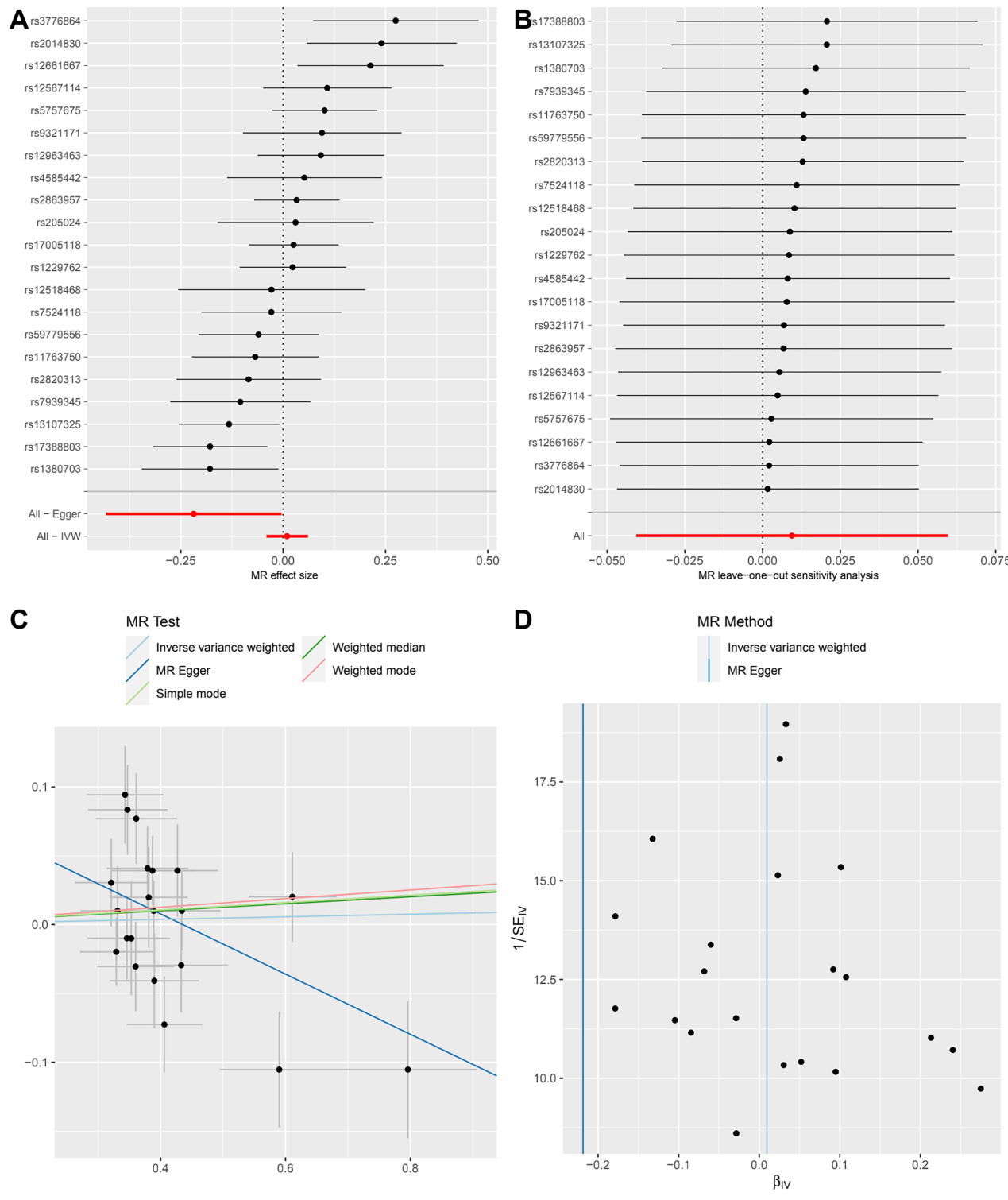
**

**Supplementary Figure S3 Forest plot (A), sensitivity analysis (B), scatter plot (C) and funnel plot (D) of the causal effect of Long sleep duration on SLE risk。**

**
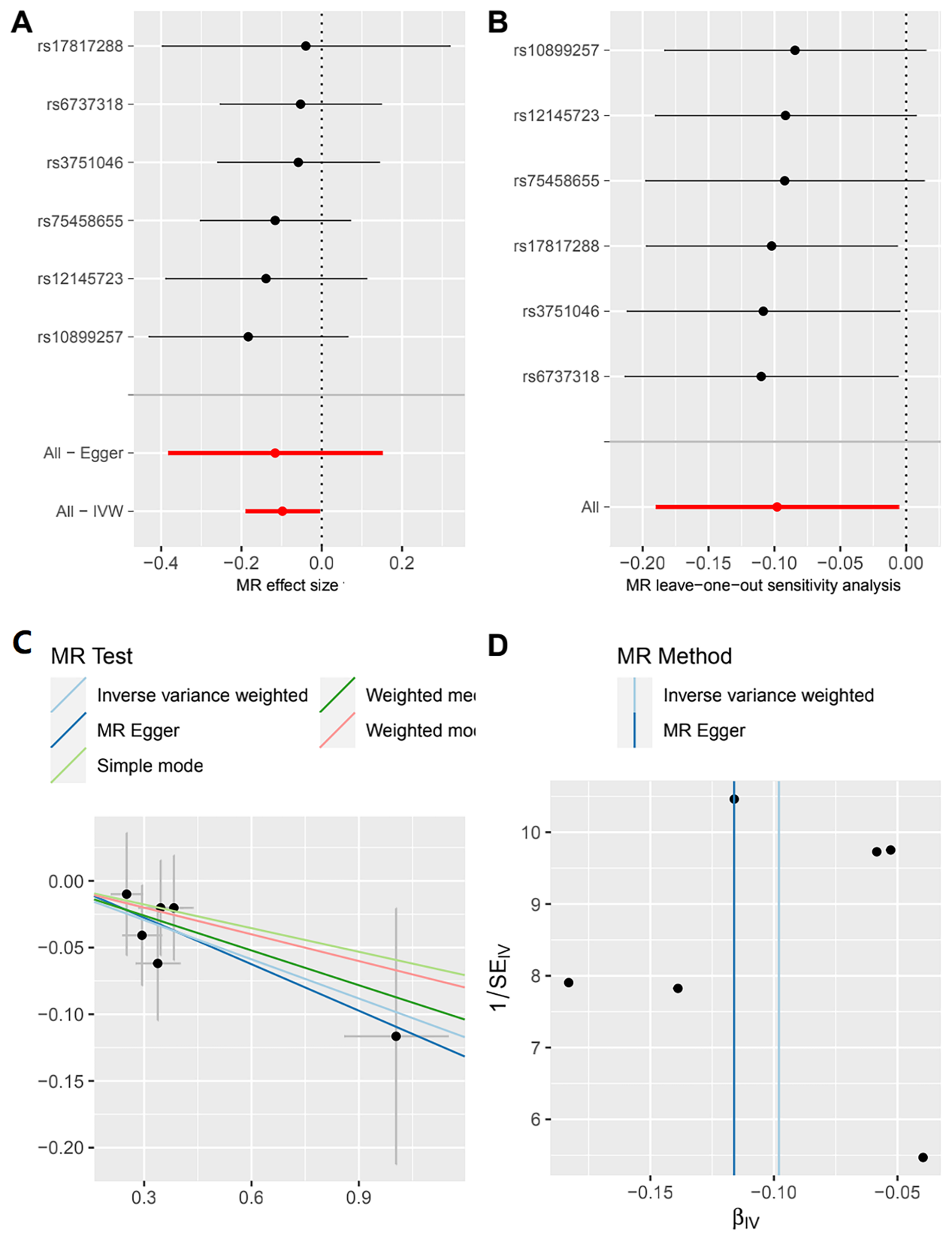
**

**Supplementary Figure S4 Forest plot (A), sensitivity analysis (B), scatter plot (C) and funnel plot (D) of the causal effect of Insomnia on SLE risk。**

**
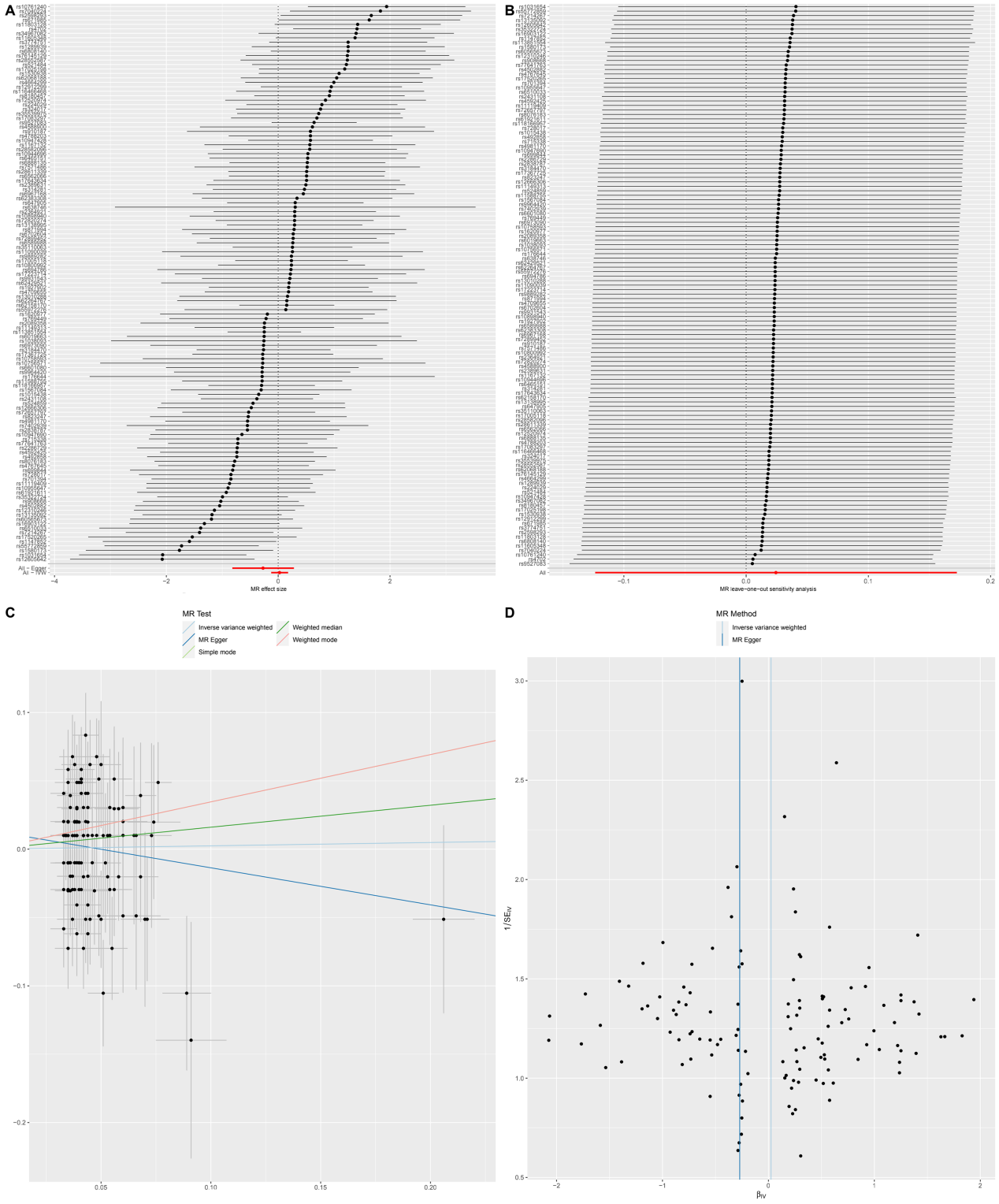
**

**Supplementary Figure S5 Forest plot (A), sensitivity analysis (B), scatter plot (C) and funnel plot (D) of the causal effect of Daytime sleepiness on SLE risk。**

**
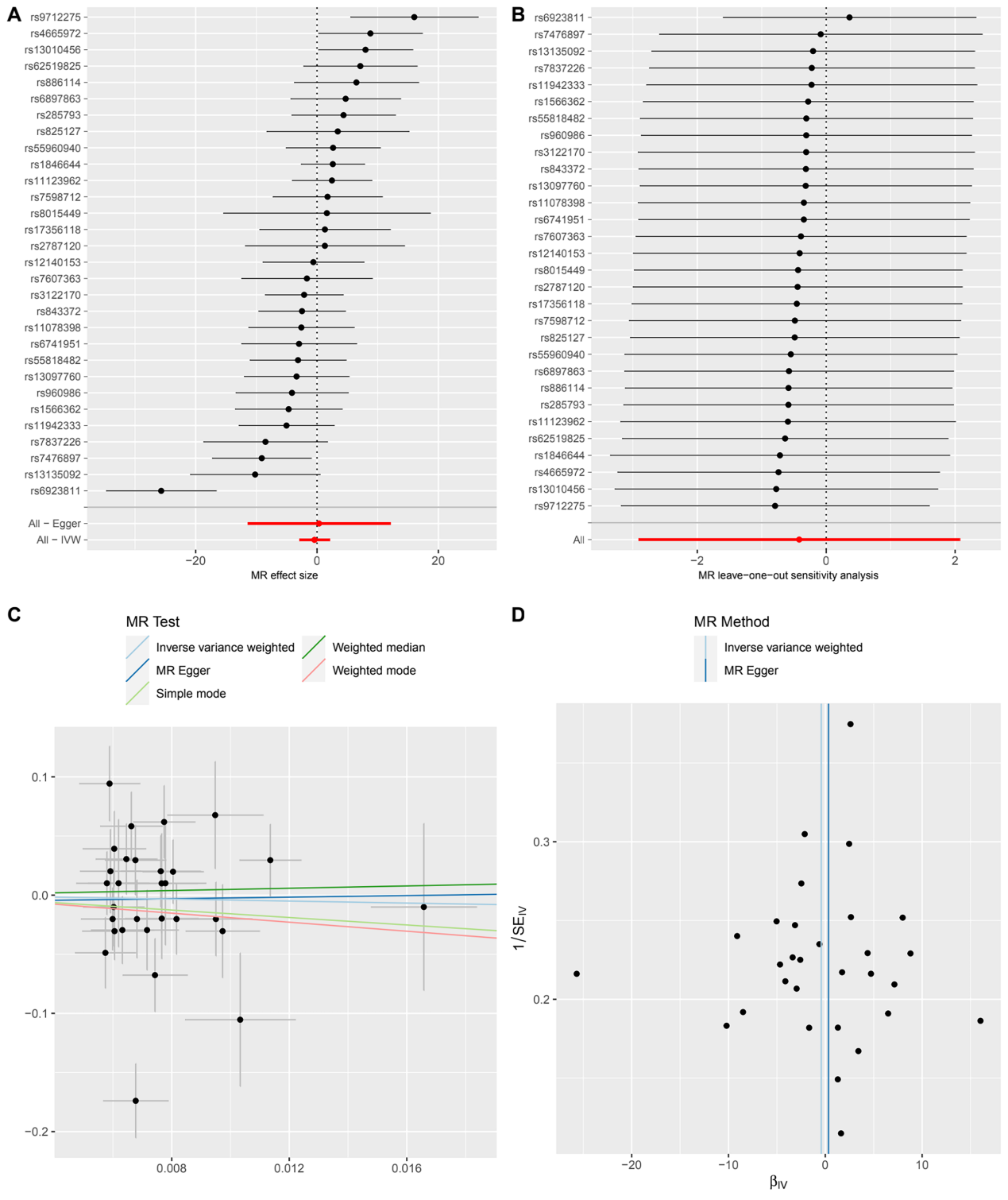
**

**Supplementary Figure S6 Forest plot (A), sensitivity analysis (B), scatter plot (C) and funnel plot (D) of the causal effect of SLE on Sleep duration risk。**

**
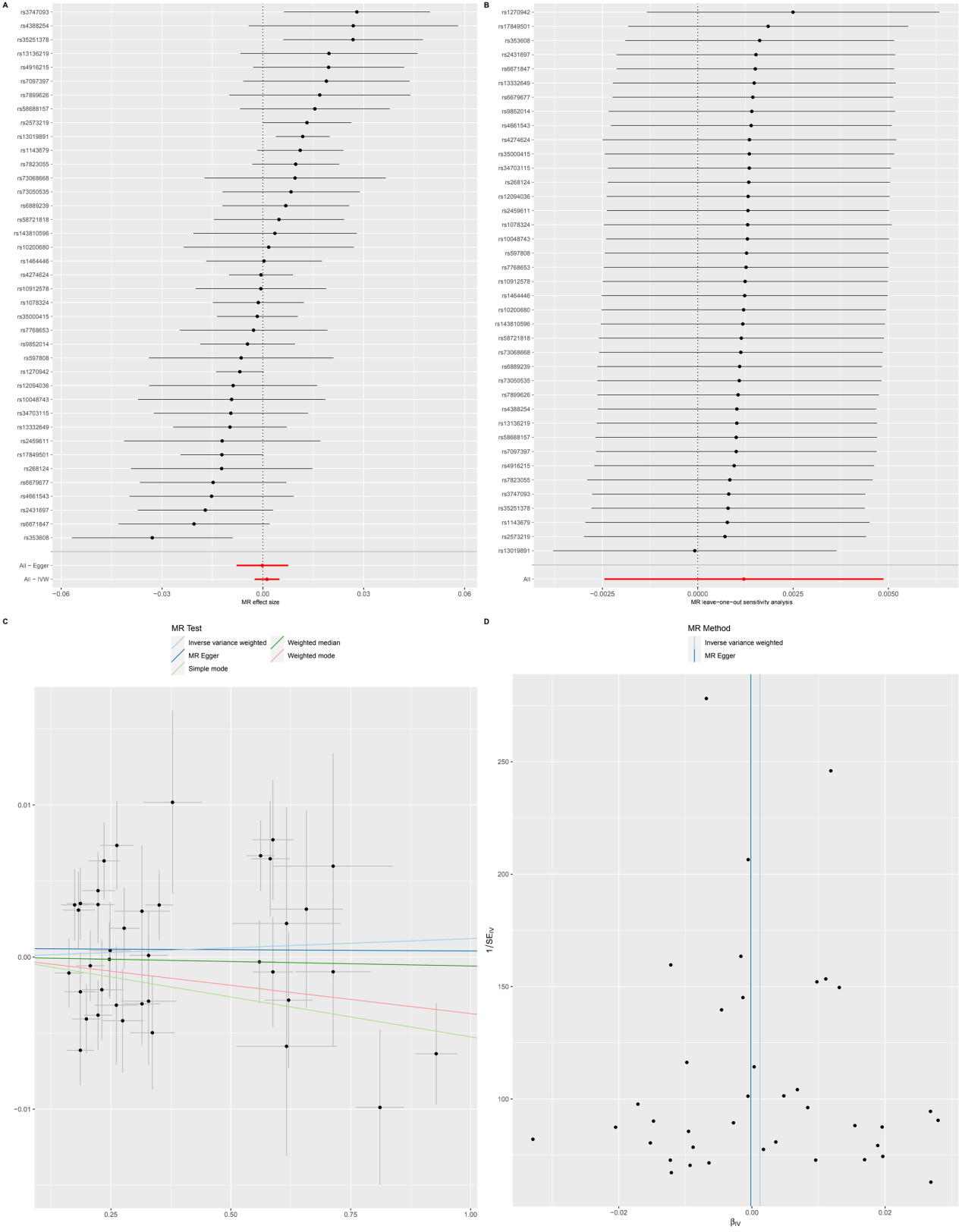
**

**Supplementary Figure S7 Forest plot (A), sensitivity analysis (B), scatter plot (C) and funnel plot (D) of the causal effect of SLE on Short sleep duration risk。**

**
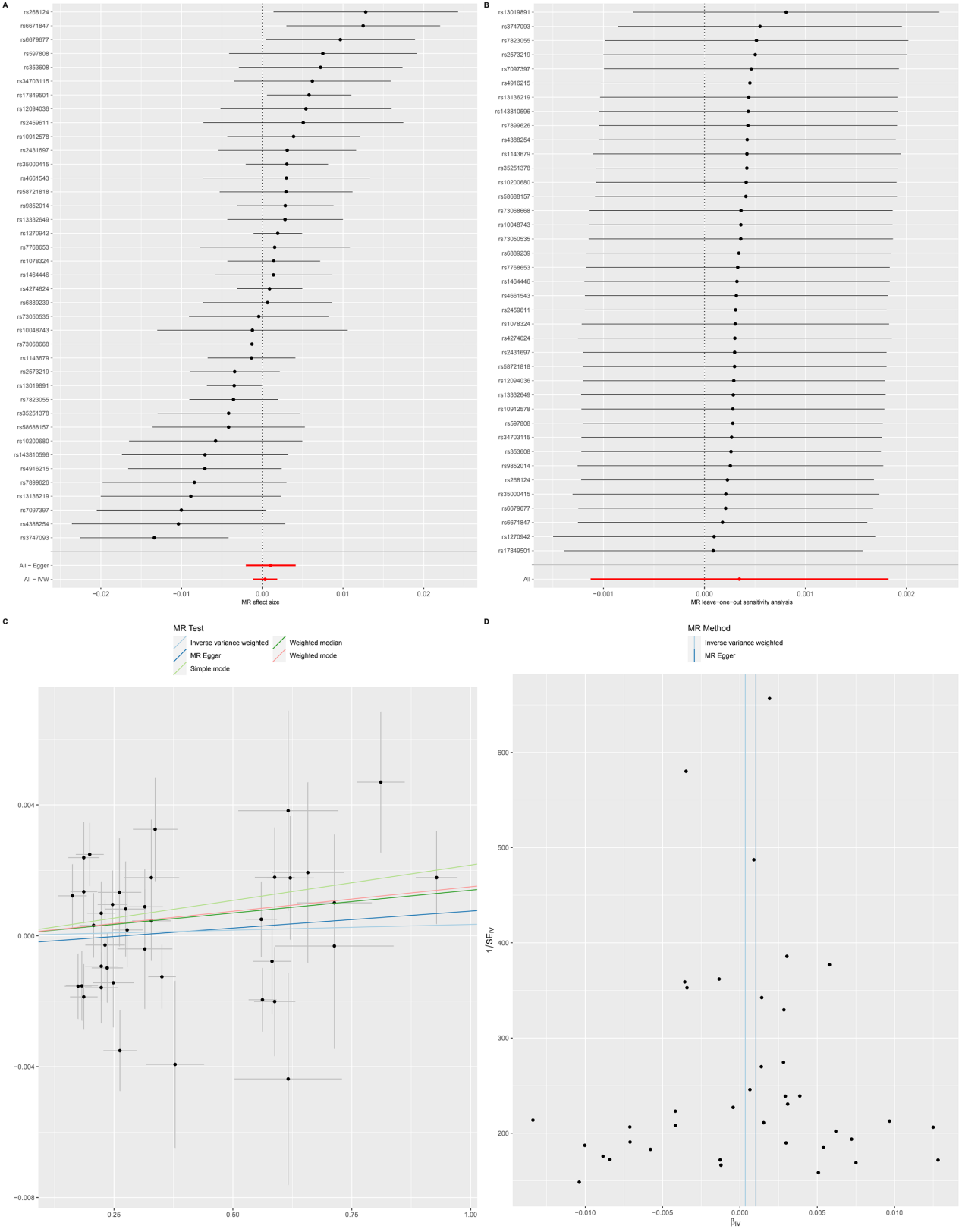
**

**Supplementary Figure S8 Forest plot (A), sensitivity analysis (B), scatter plot (C) and funnel plot (D) of the causal effect of SLE on Long sleep duration risk。**

**
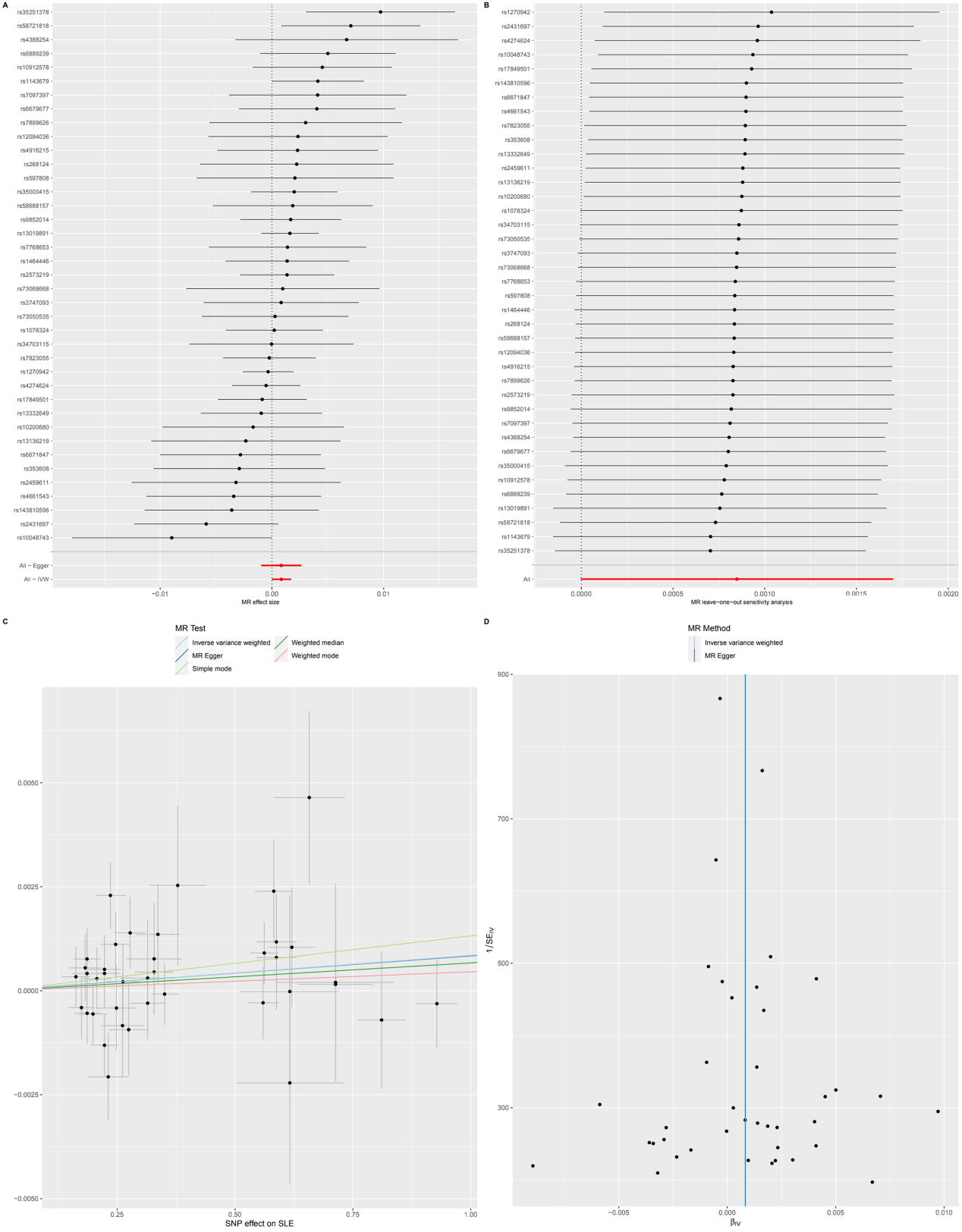
**

**Supplementary Figure S9 Forest plot (A), sensitivity analysis (B), scatter plot (C) and funnel plot (D) of the causal effect of SLE on Insomnia risk。**

**
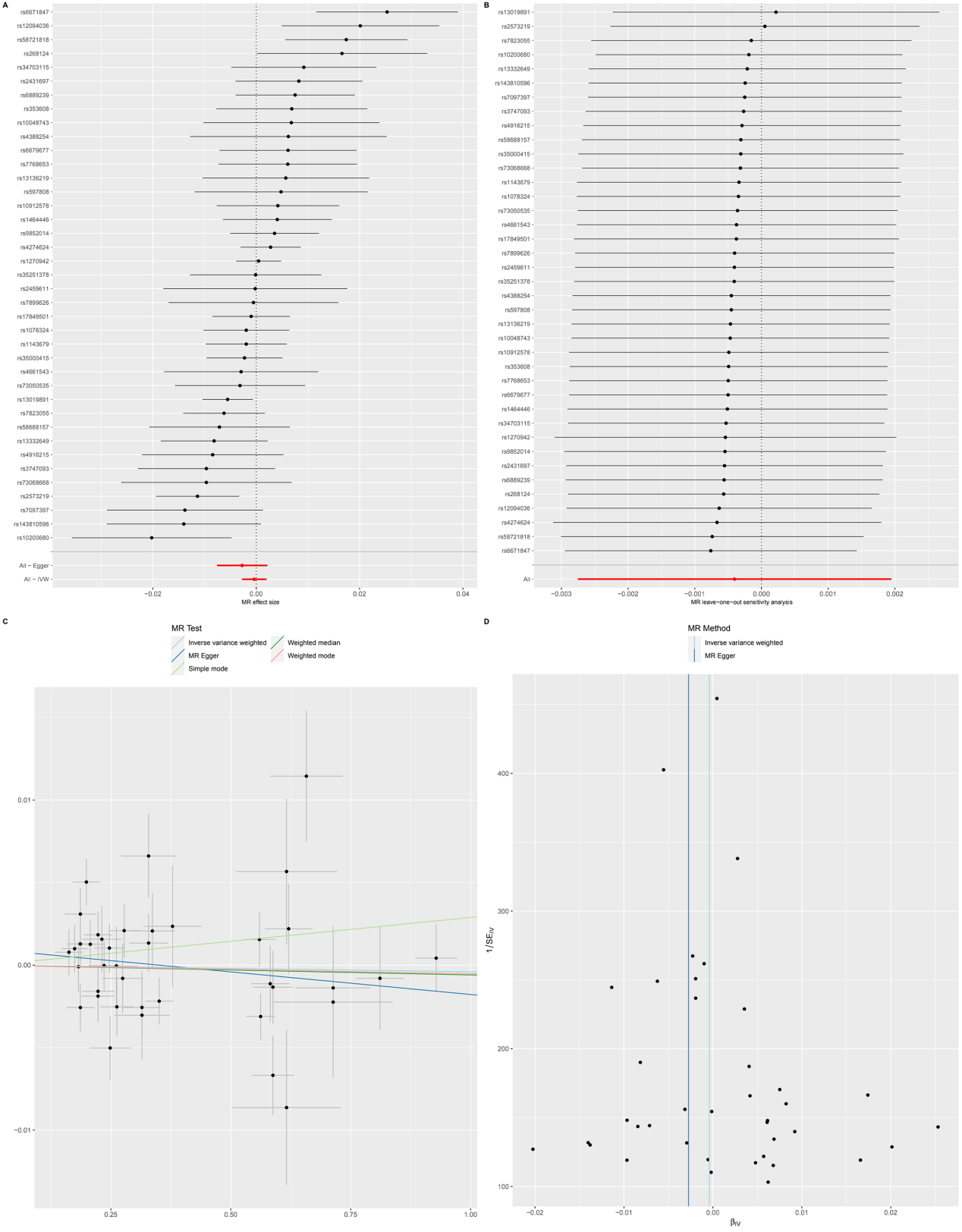
**

**Supplementary Figure S10 Forest plot (A), sensitivity analysis (B), scatter plot (C) and funnel plot (D) of the causal effect of SLE on Daytime sleepiness risk。**

**
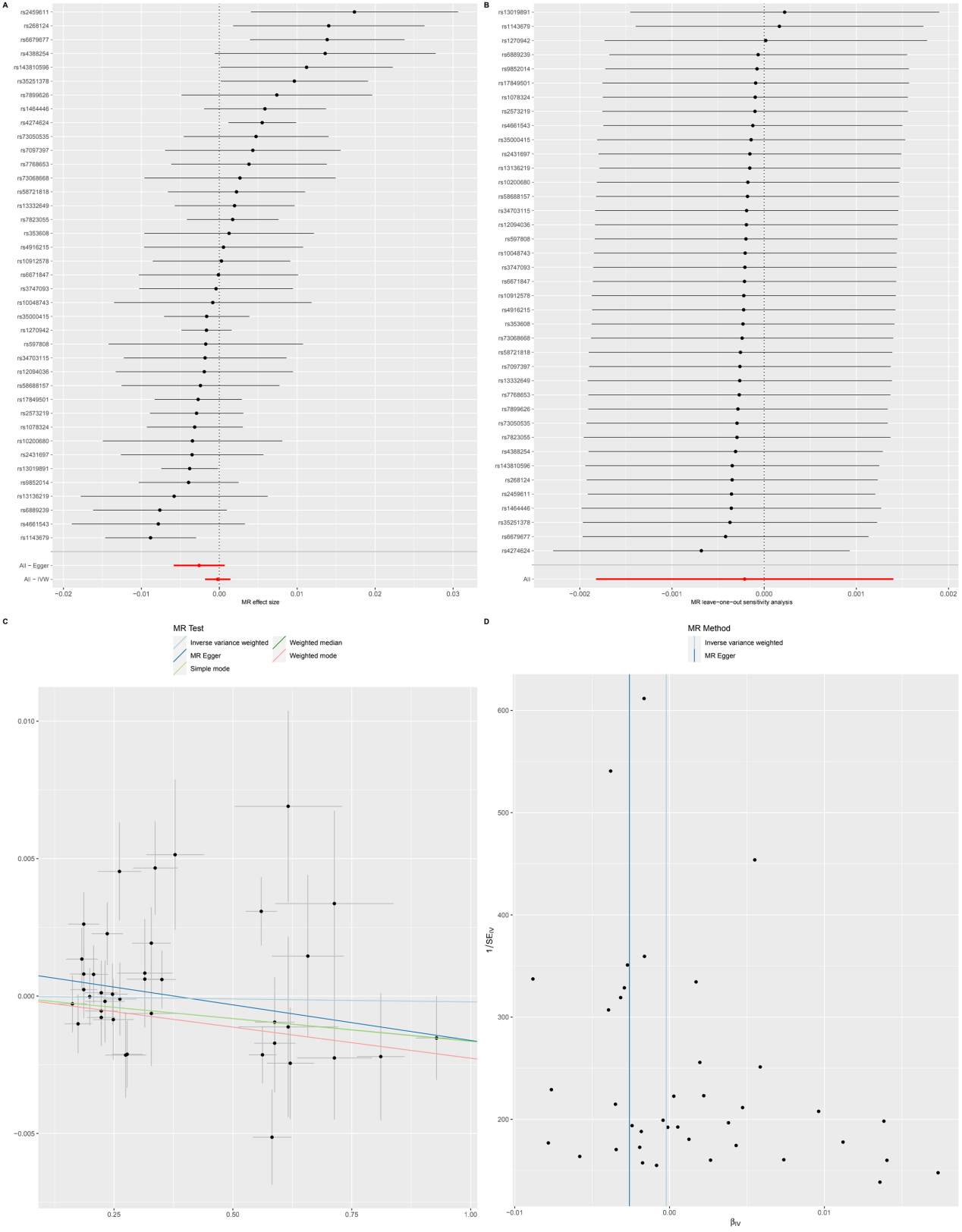
**

**Supplementary Figure S11 Forest plot (A), sensitivity analysis (B), scatter plot (C) and funnel plot (D) of the causal effect of Chronotype on SLE risk after removing SNPs with potential pleiotropy。**

**
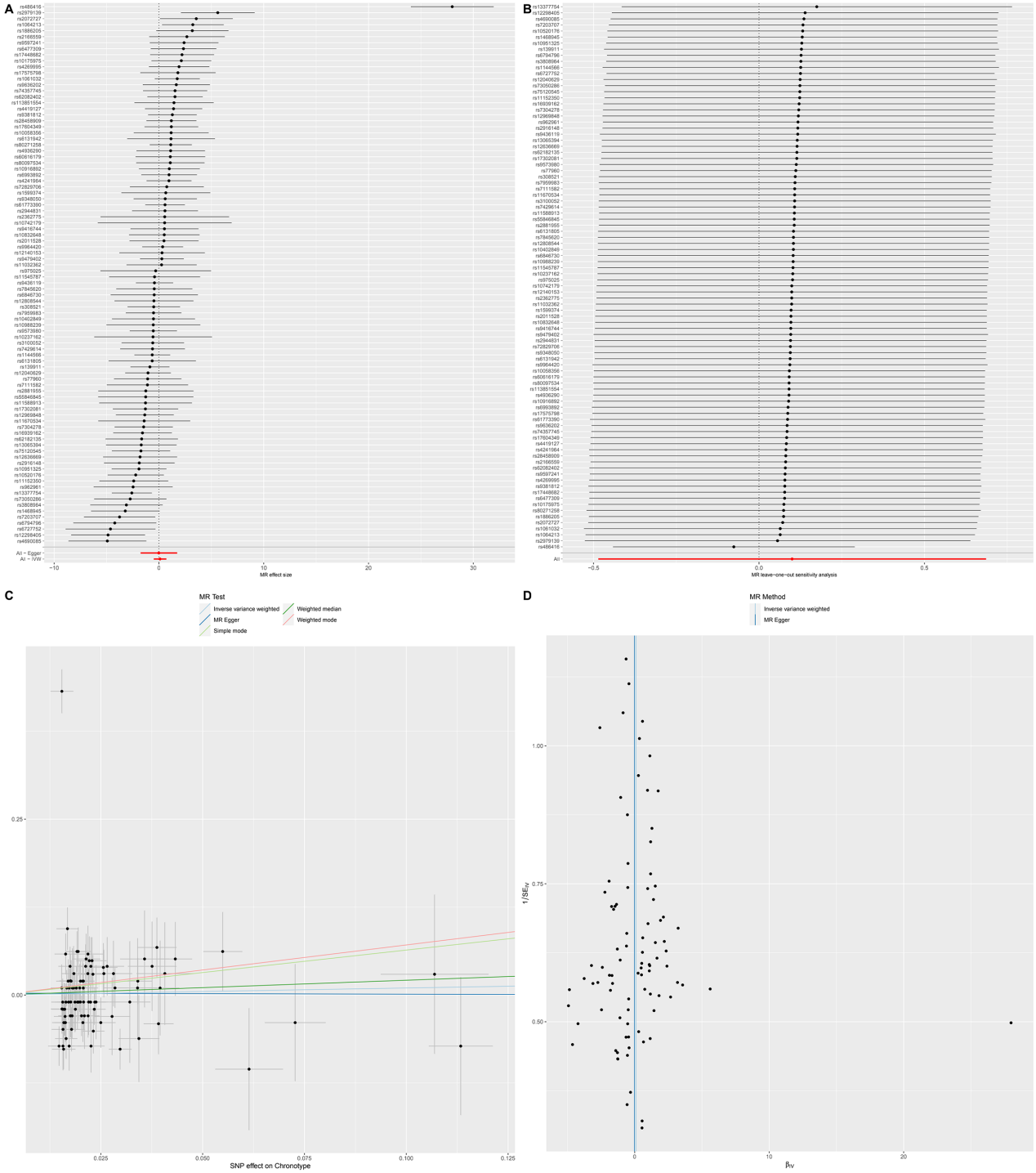
**

**Supplementary Figure S12 Forest plot (A), sensitivity analysis (B), scatter plot (C) and funnel plot (D) of the causal effect of Sleep duration on SLE risk after removing SNPs with potential pleiotropy。**

**
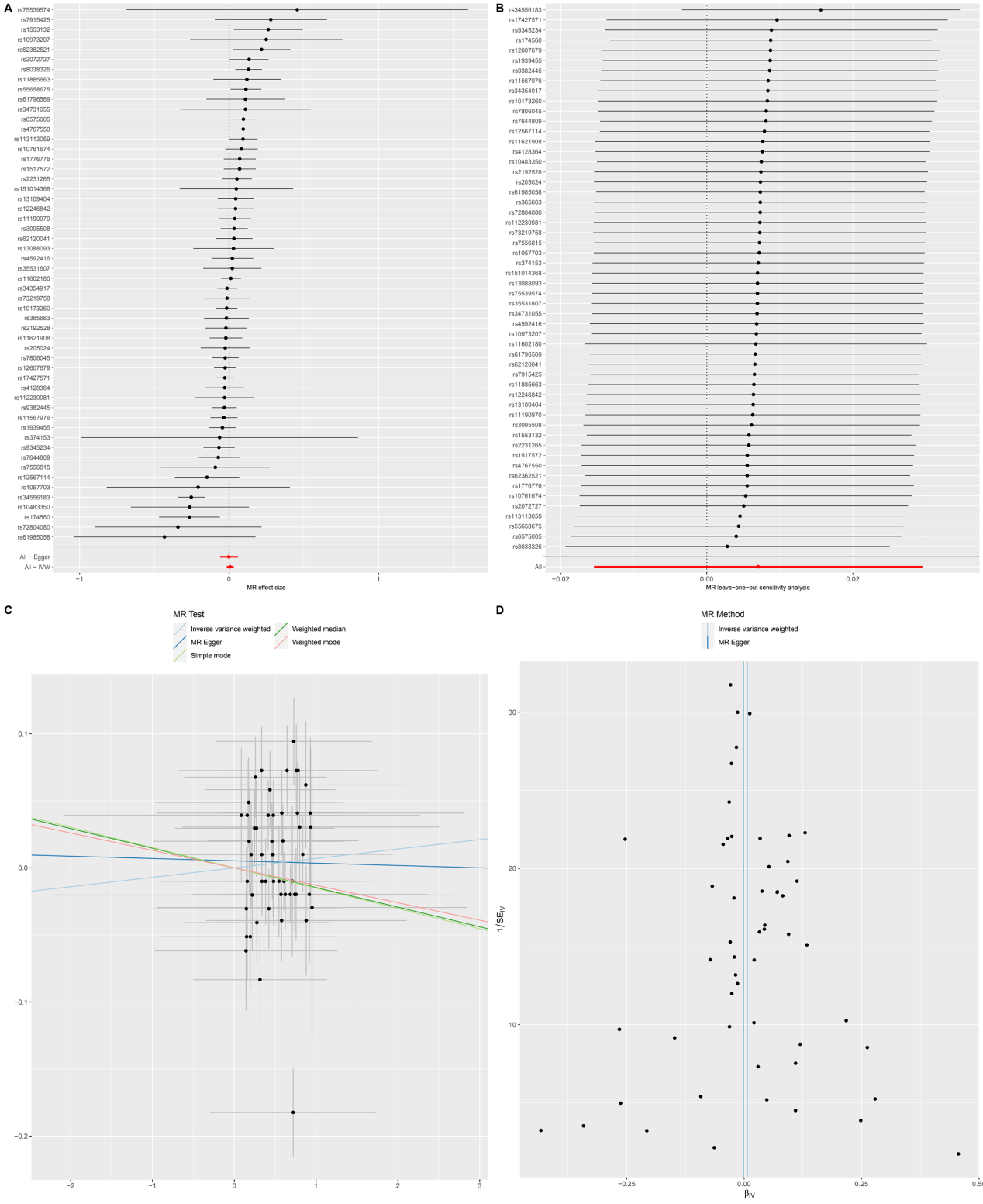
**

**Supplementary Figure S13 Forest plot (A), sensitivity analysis (B), scatter plot (C) and funnel plot (D) of the causal effect of Short sleep duration on SLE risk after removing SNPs with potential pleiotropy。**

**
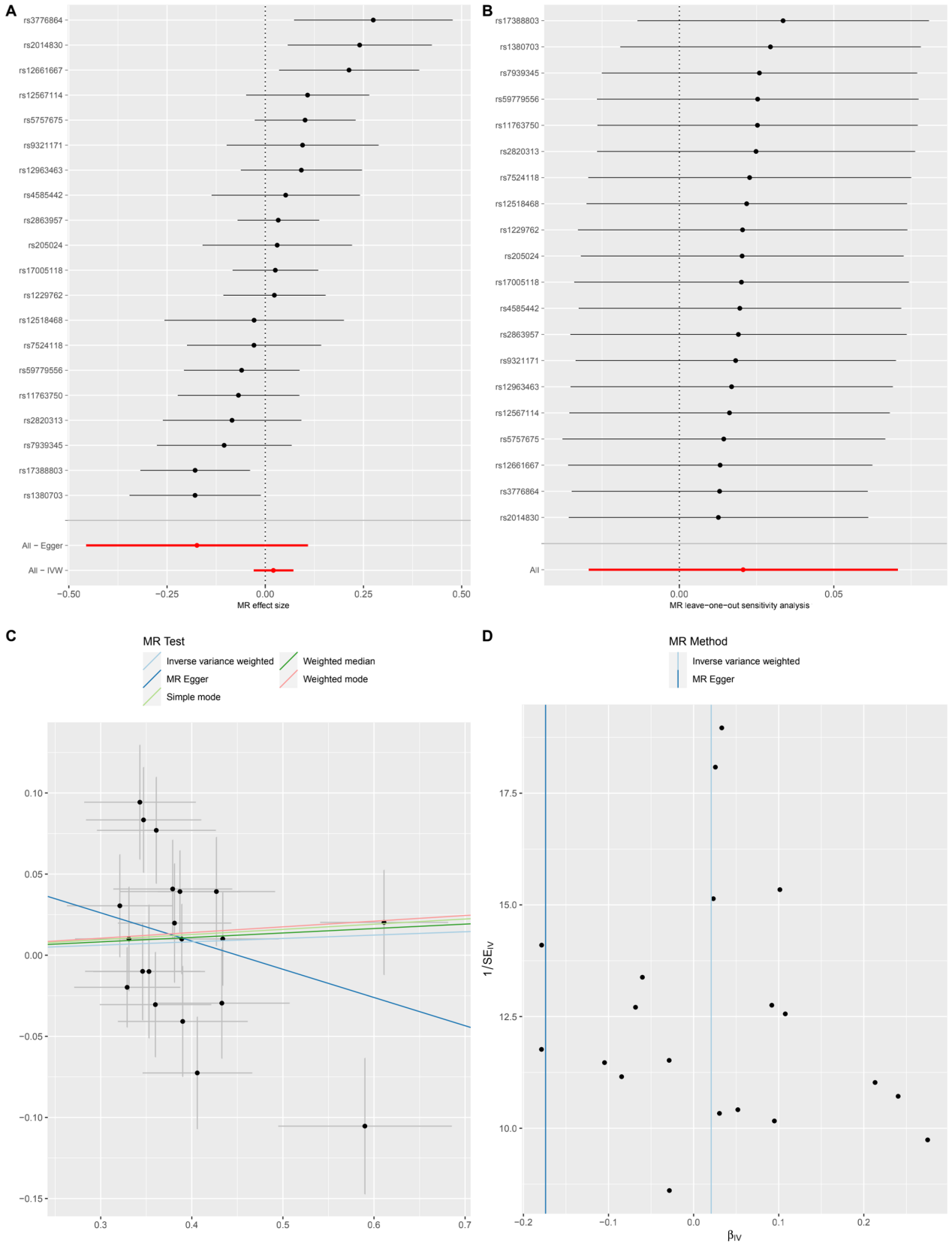
**

**Supplementary Figure S14 Forest plot (A), sensitivity analysis (B), scatter plot (C) and funnel plot (D) of the causal effect of Insomnia on SLE risk after removing SNPs with potential pleiotropy。**

**
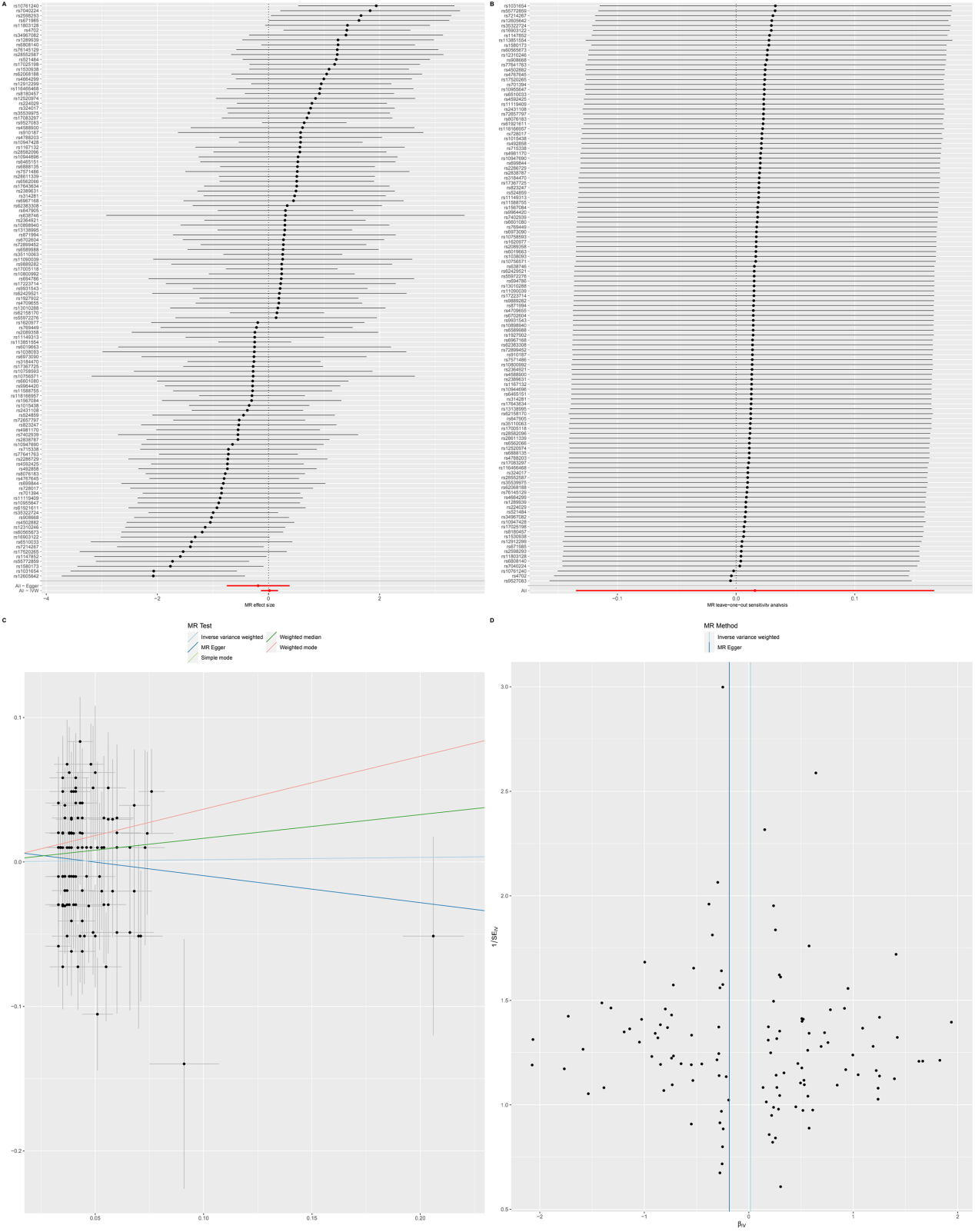
**

**Supplementary Figure S15 Forest plot (A), sensitivity analysis (B), scatter plot (C) and funnel plot (D) of the causal effect of Daytime sleepiness on SLE risk after removing SNPs with potential pleiotropy。**

**
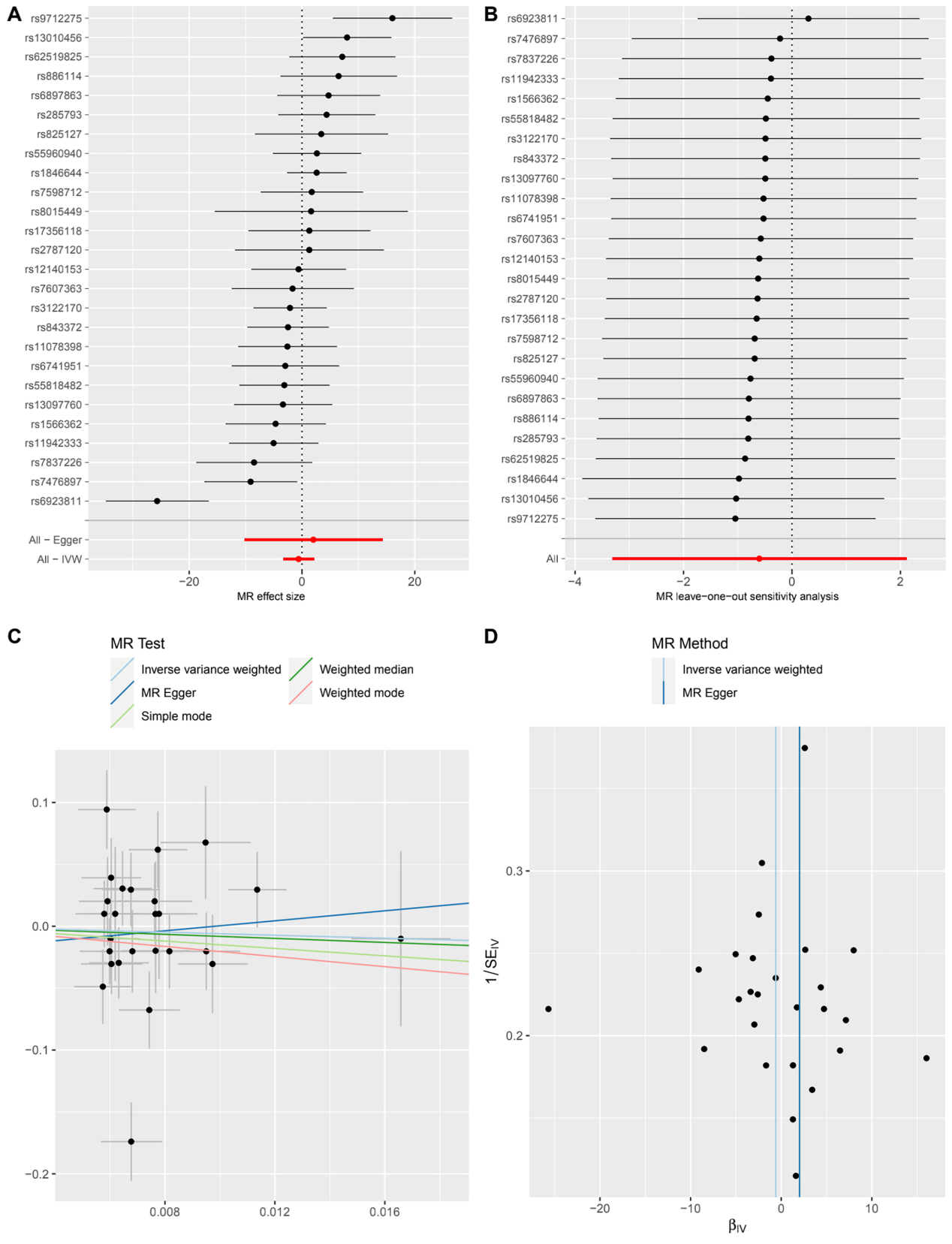
**

**Supplementary Figure S16 Forest plot (A), sensitivity analysis (B), scatter plot (C) and funnel plot (D) of the causal effect of SLE on Chronotype risk after removing SNPs with potential pleiotropy。**

**
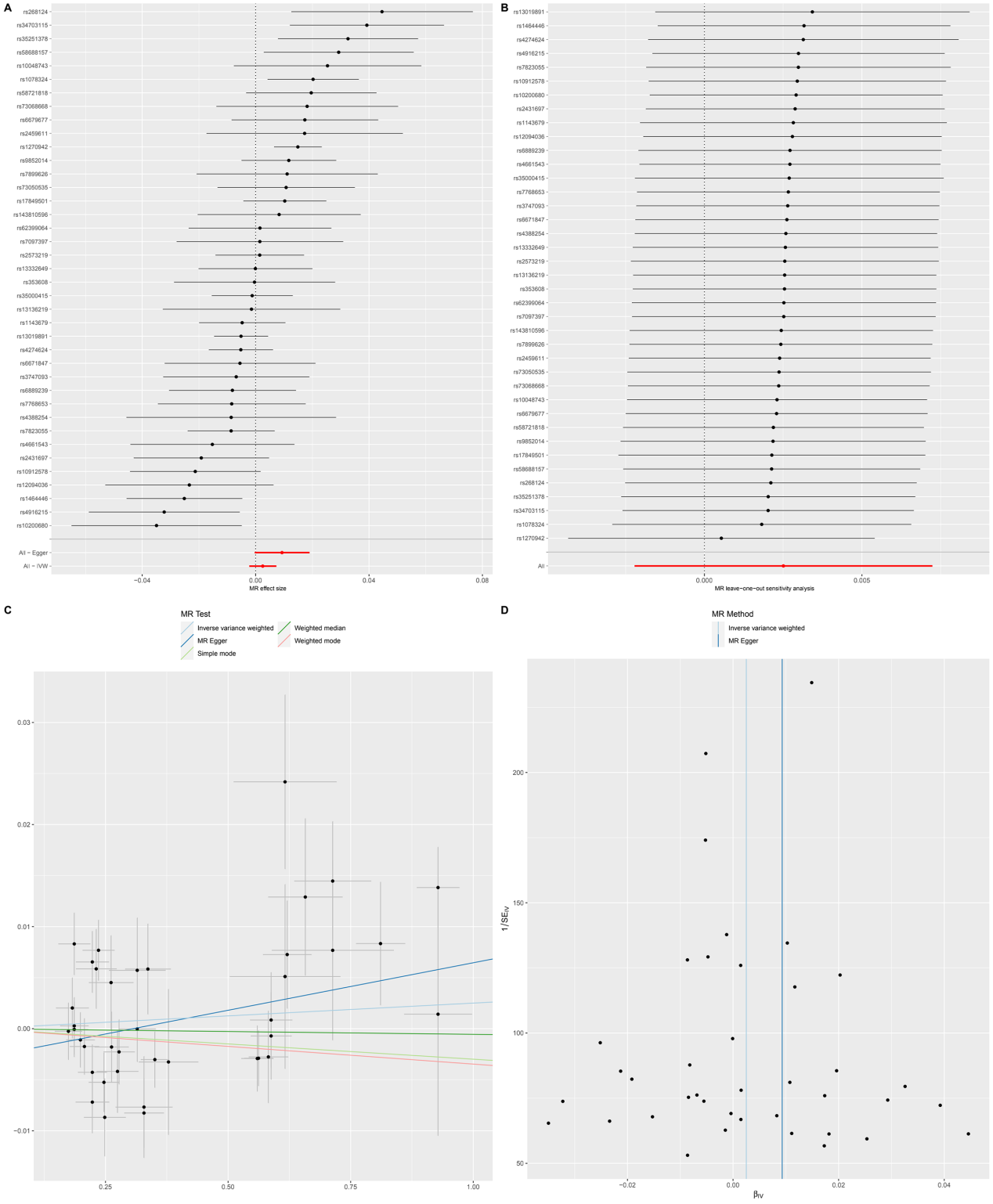
**

**Supplementary Figure S17 Forest plot (A), sensitivity analysis (B), scatter plot (C) and funnel plot (D) of the causal effect of SLE on Sleep duration risk after removing SNPs with potential pleiotropy。**

**
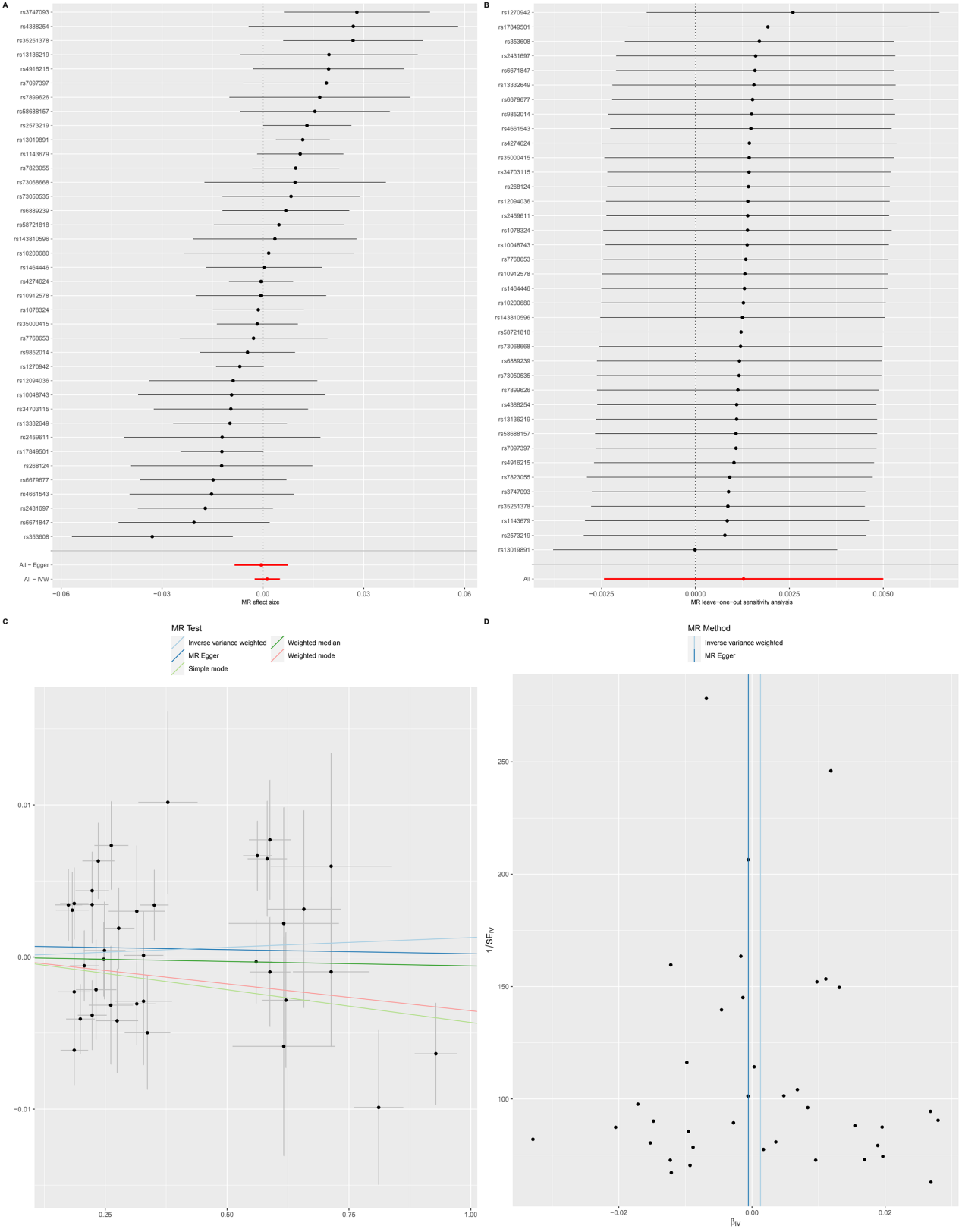
**

**Supplementary Figure S18 Forest plot (A), sensitivity analysis (B), scatter plot (C) and funnel plot (D) of the causal effect of SLE on Short sleep duration risk after removing SNPs with potential pleiotropy。**

**
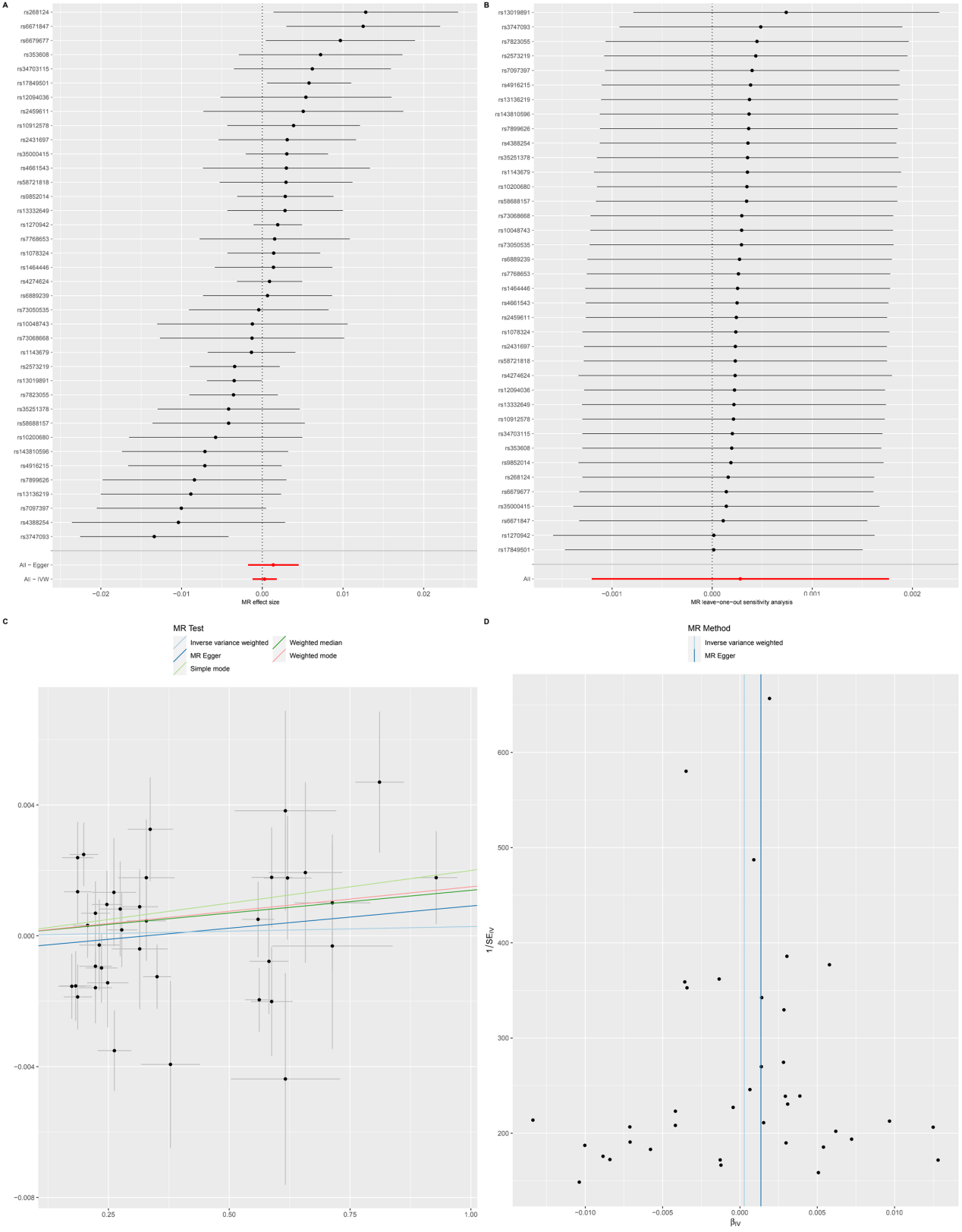
**

**Supplementary Figure S19 Forest plot (A), sensitivity analysis (B), scatter plot (C) and funnel plot (D) of the causal effect of SLE on Long sleep duration risk after removing SNPs with potential pleiotropy。**

**
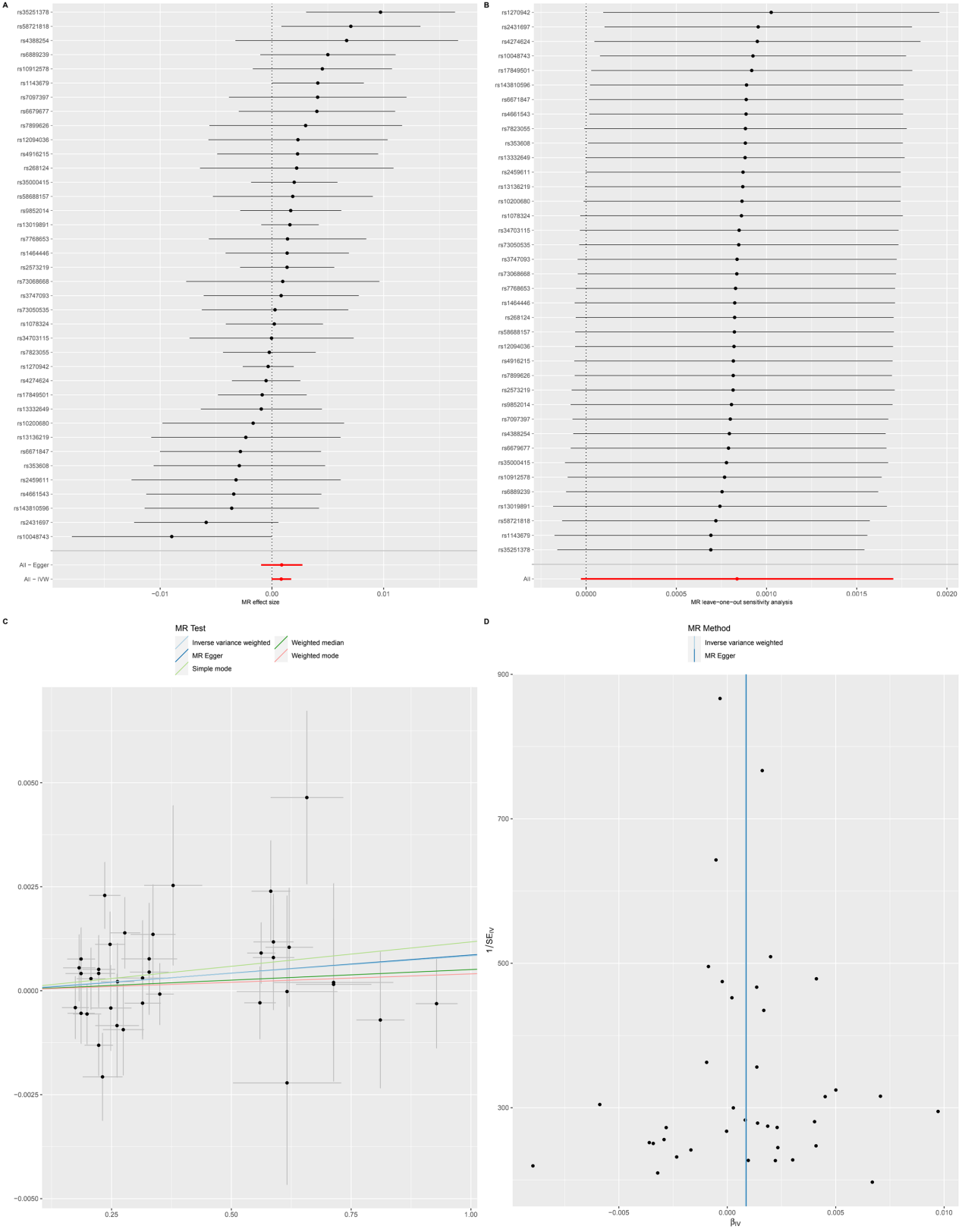
**

**Supplementary Figure S20 Forest plot (A), sensitivity analysis (B), scatter plot (C) and funnel plot (D) of the causal effect of SLE on Insomnia risk after removing SNPs with potential pleiotropy。**

**
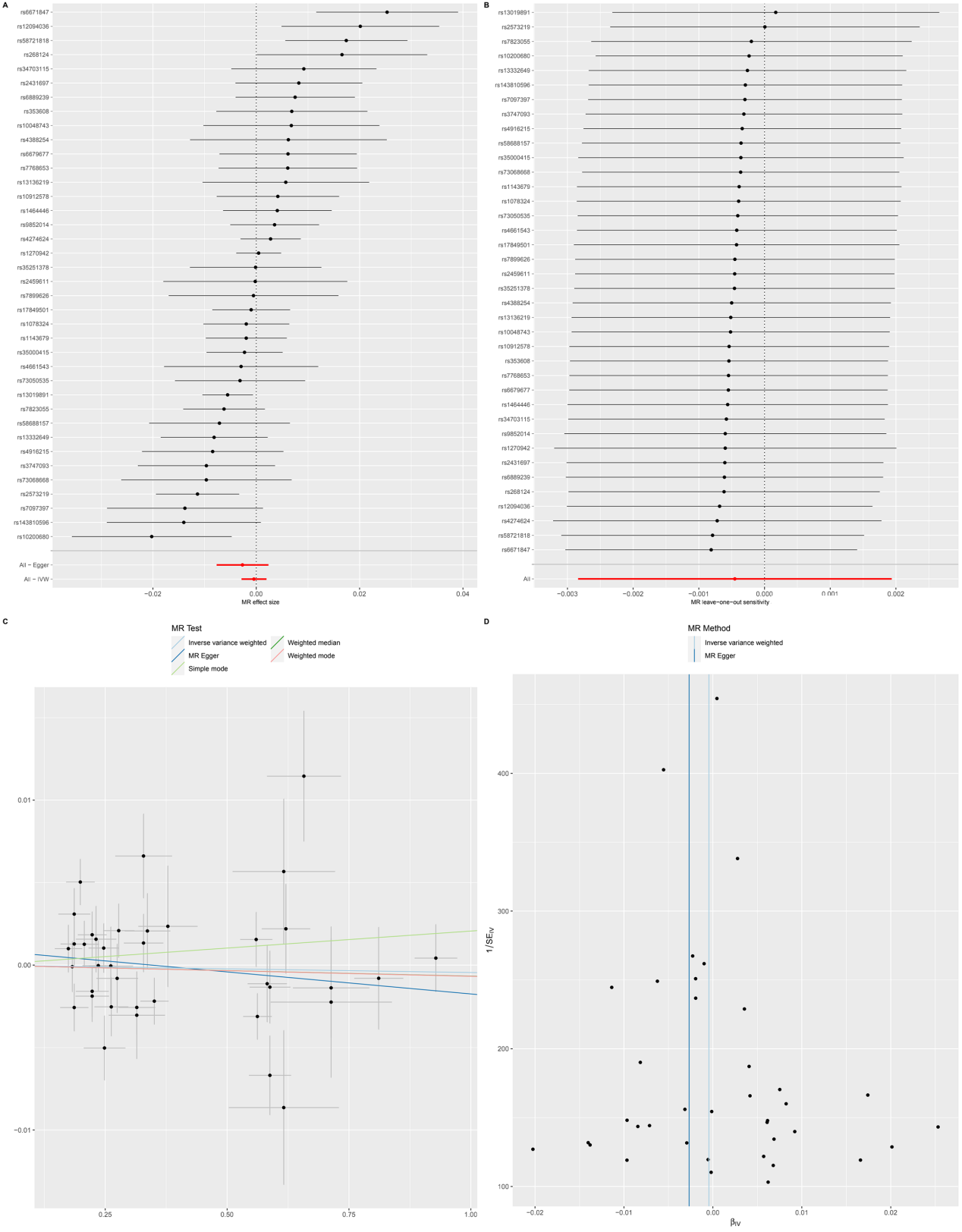
**

**Supplementary Figure S21 Forest plot (A), sensitivity analysis (B), scatter plot (C) and funnel plot (D) of the causal effect of SLE on Daytime sleepiness risk after removing SNPs with potential pleiotropy。**

**
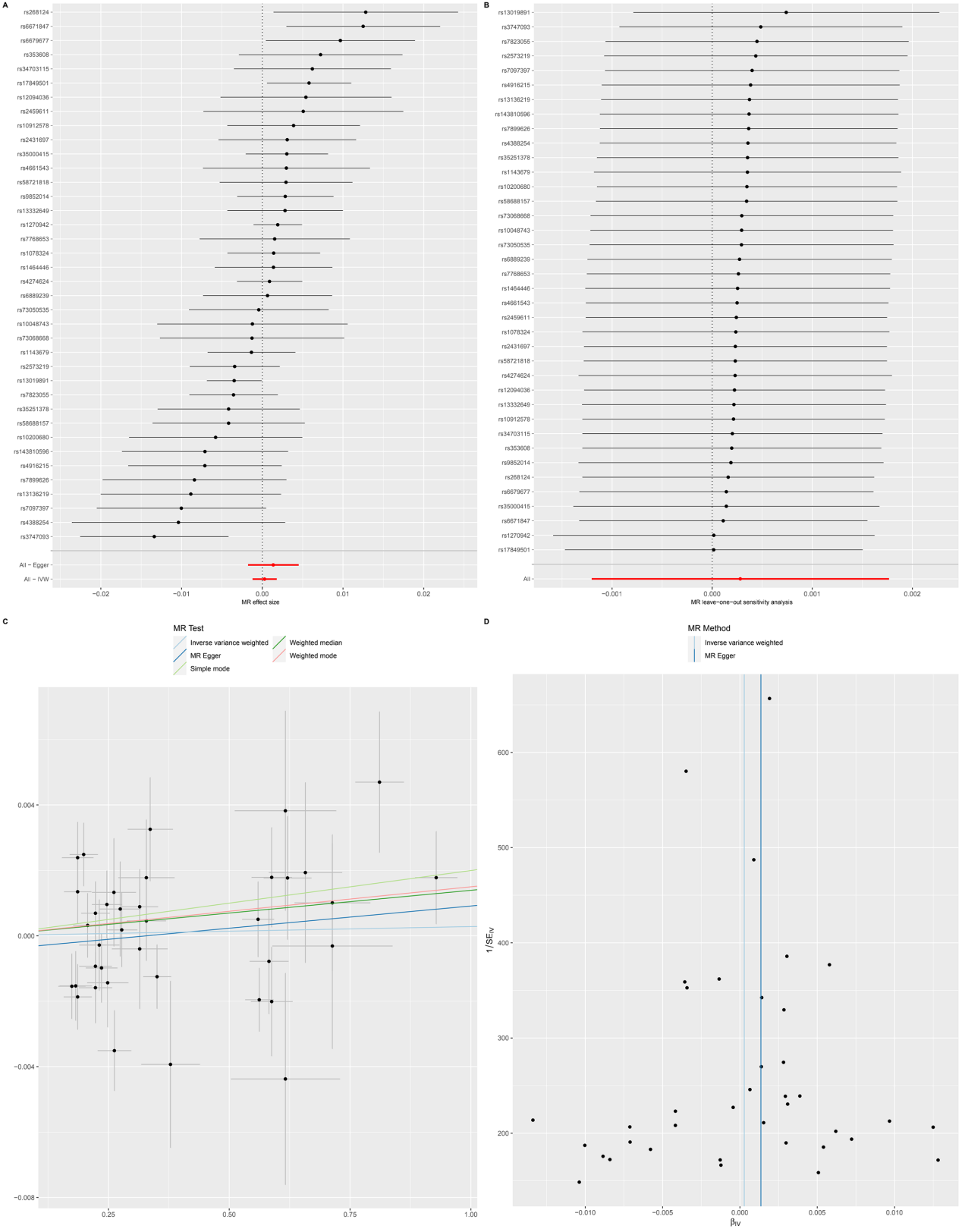
**
